# Correction: Antisense oligonucleotide therapy rescues disturbed brain rhythms and sleep in juvenile and adult mouse models of Angelman syndrome

**DOI:** 10.7554/eLife.104349

**Published:** 2024-10-15

**Authors:** Dongwon Lee, Wu Chen, Heet Naresh Kaku, Xinming Zhuo, Eugene S Chao, Armand Soriano, Allen Kuncheria, Stephanie Flores, Joo Hyun Kim, Armando Rivera, Frank Rigo, Paymaan Jafar-nejad, Arthur L Beaudet, Matthew S Caudill, Mingshan Xue

**Keywords:** Mouse

Lee D, Chen W, Kaku HN, Zhuo X, Chao ES, Soriano A, Kuncheria A, Flores S, Kim JH, Rivera A, Rigo F, Jafar-nejad P, Beaudet AL, Caudill MS, Xue M. 2023. Antisense oligonucleotide therapy rescues disturbed brain rhythms and sleep in juvenile and adult mouse models of Angelman syndrome. *eLife* **12**:e81892. doi: 10.7554/eLife.81892.

Published 3 January 2023

After publication, we found three errors in Figure 3—figure supplement 5 and Figure 8.

1. In Figure 3-figure supplement 5, male and female mice are indicated by filled and open symbols, respectively. Two wildtype male mice injected with 500 μg control ASO were erroneously labelled as female mice. We have now corrected these two data points to filled circles. There are no changes in the text or figure legend.

The corrected Figure 3—figure supplement 5 is shown here:

**Figure 1:**
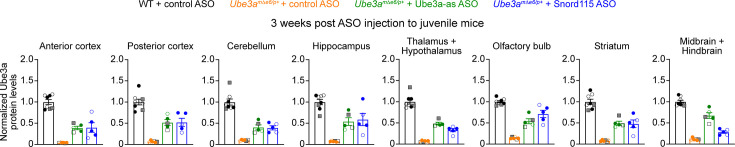


The originally published Figure 3—figure supplement 5 is shown for reference:

**Figure 2:**
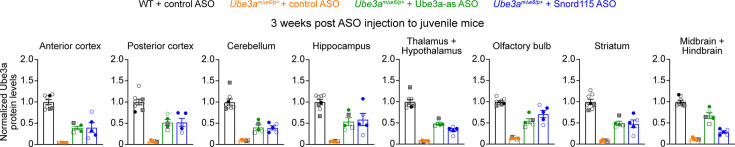

1. In Figure 8A, the plot for the relationship between ‘Ube3a protein levels’ and ‘normalized relative power in the γ2 (50–100 Hz) band’ was duplicated from the adjacent plot for the γ1 (25–50 Hz) band. This error occurred when the plot was copied from the Prism program into the Adobe Illustrator program during the assembly of Figure 8A. The statistical results listed above the plot was correct in the originally published figure, as it was separately and correctly copied and pasted. We have now changed the γ2 plot to the correct plot. There are no changes in the text or figure legend.
2. In Figure 8B, the plot for the relationship between ‘Ube3a protein levels’ and ‘normalized time in REM-Dark’ used the incorrect data for the normalized time in REM-Dark. The analysis of sleep time initially had a small error in the time epochs, which was discovered and corrected. The correct sleep time was used in the Figure 6 and Figure 8B ‘REM-Light’ plot, but unfortunately in Figure 8B ‘REM-Dark’ plot, the REM sleep time in the dark phase was not updated from the previous incorrect data. We have now re-plotted the relationship between Ube3a protein levels and the correct REM sleep time in the dark phase to generate the correct plot and statistics. Note, this correction does not change the conclusion of this result because the changes in sleep time were small. There are no changes in the text or figure legend.

The corrected Figure 8 is shown here:

**Figure 3:**
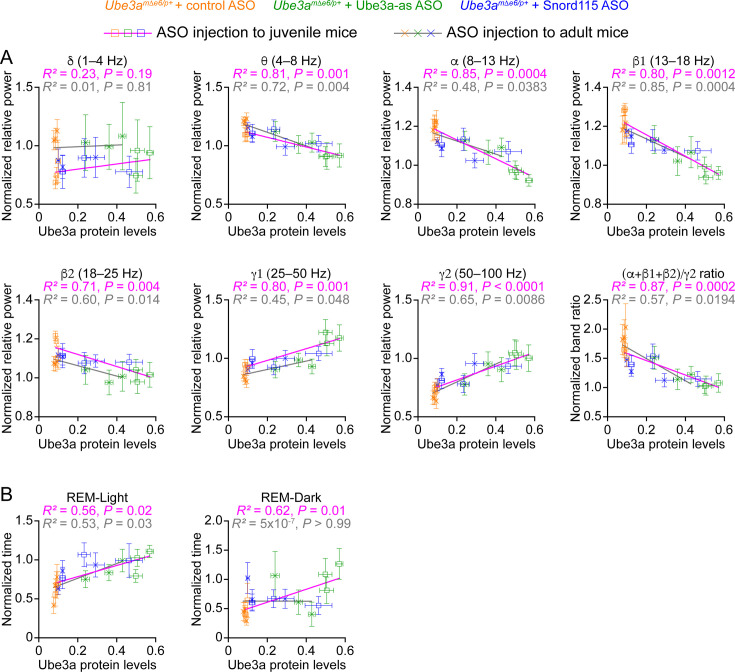


The originally published Figure 8 is shown for reference:

**Figure 4:**
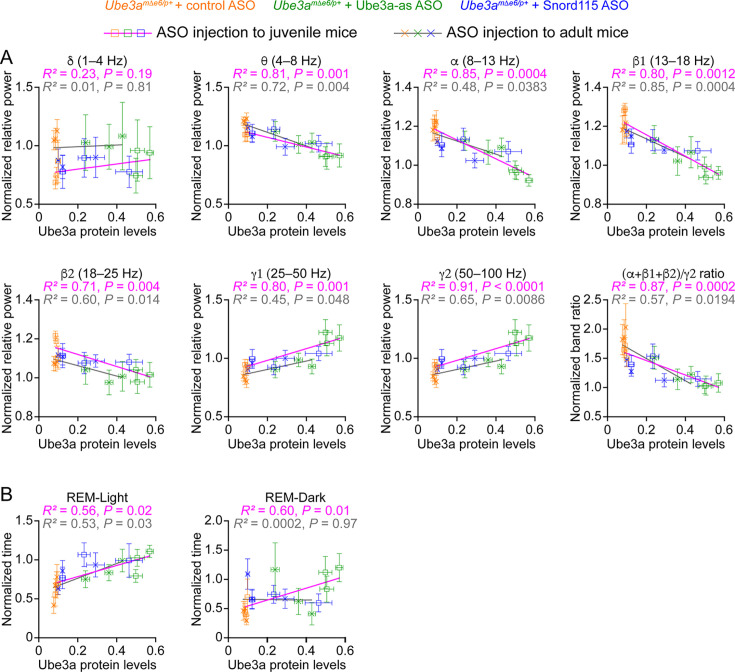


The article has been corrected accordingly.

