# Supplementary figures and images for: Antisense oligonucleotide therapy rescues disturbed brain rhythms and sleep in juvenile and adult mouse models of Angelman syndrome
Source: eLife. 2023 Jan 3;12:e81892. doi: 10.7554/eLife.81892 (PMC9904759; doi:10.7554/eLife.81892)

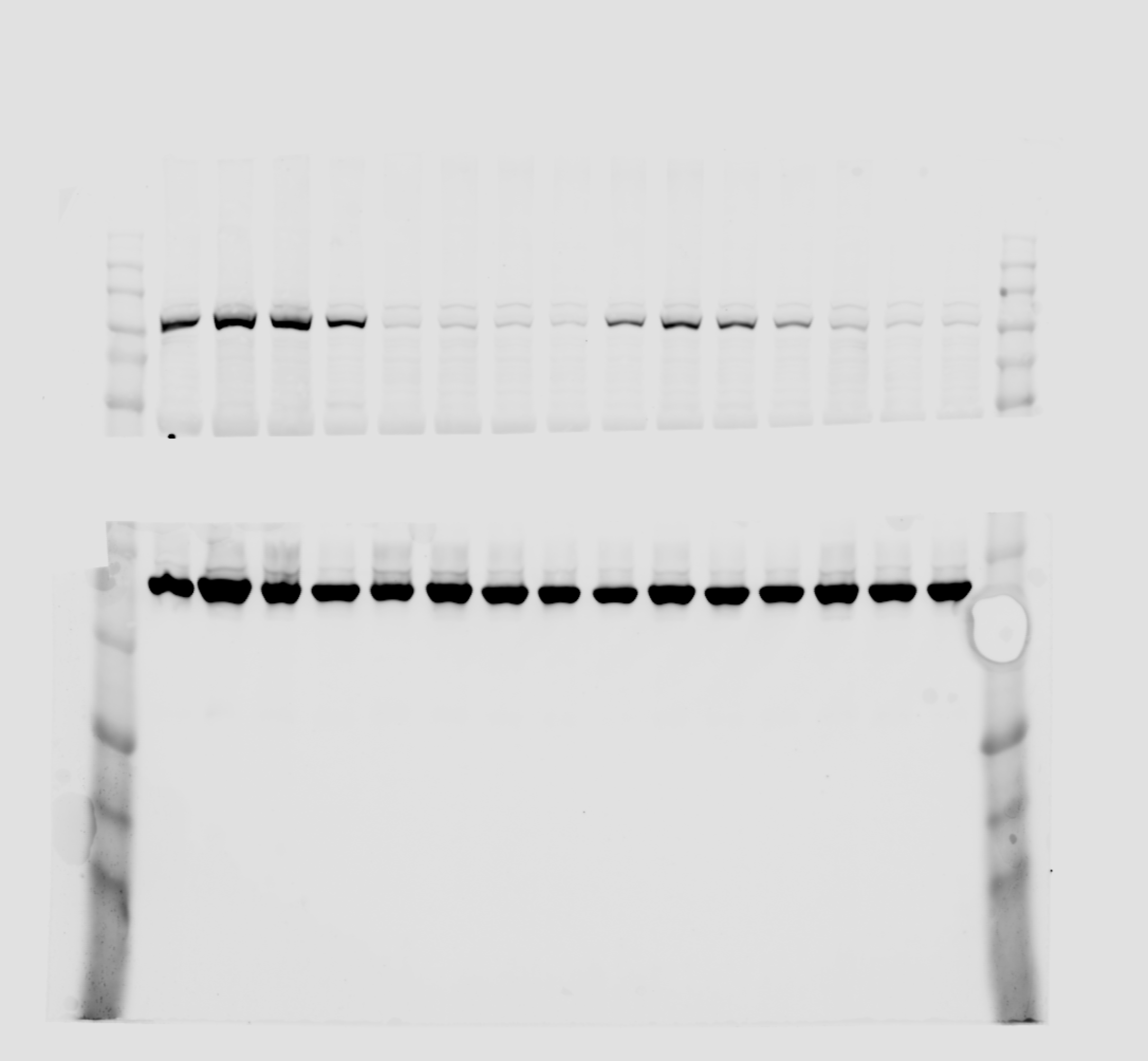

Supplement: Figure 3—source data 1. — The dashed boxes indicate the areas of blots presented in the figure. [file elife-81892-fig3-data1.zip › Figure 3-source data 1/113-1.tif]

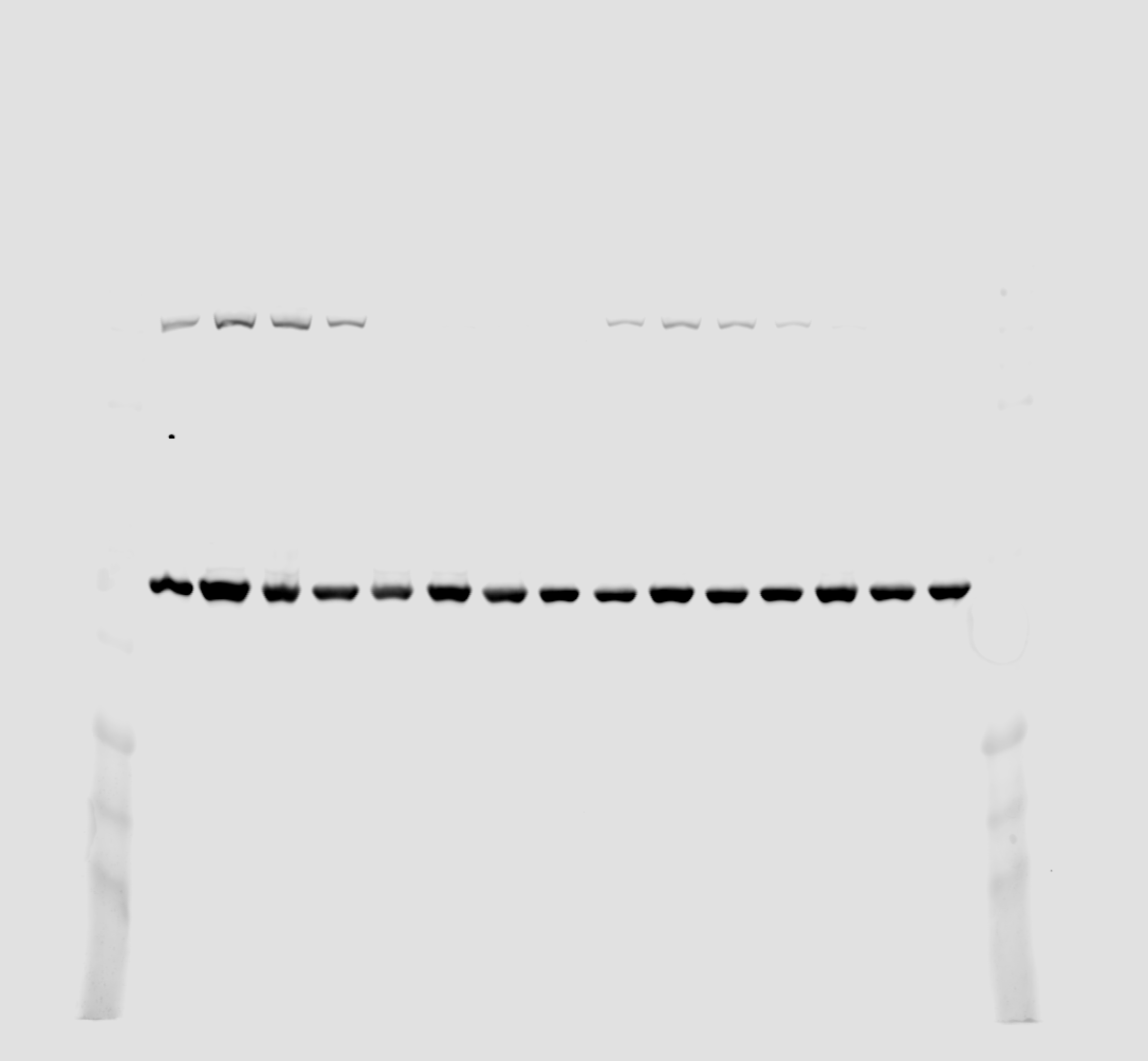

Supplement: Figure 3—source data 1. — The dashed boxes indicate the areas of blots presented in the figure. [file elife-81892-fig3-data1.zip › Figure 3-source data 1/113-2.tif]

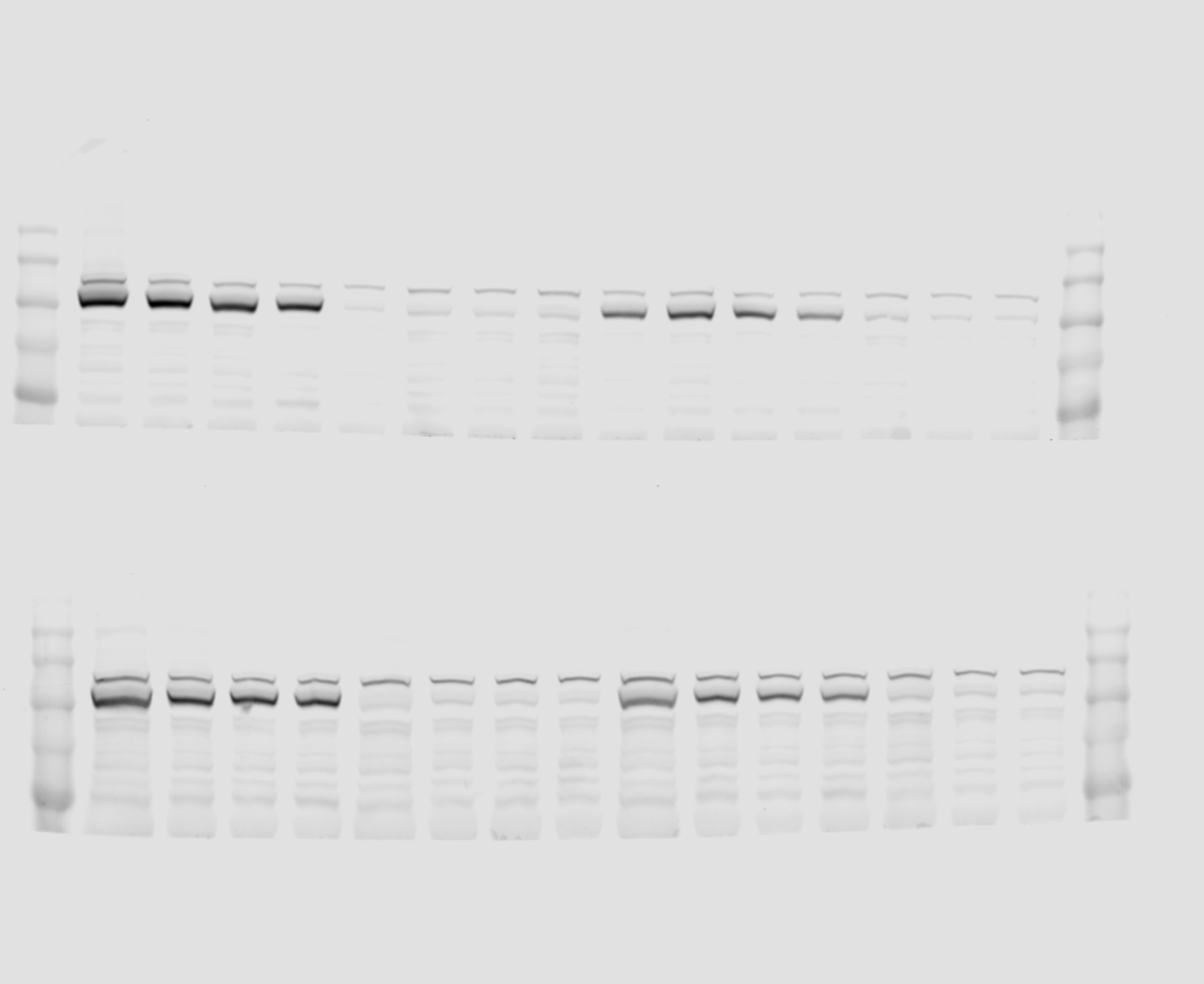

Supplement: Figure 3—source data 1. — The dashed boxes indicate the areas of blots presented in the figure. [file elife-81892-fig3-data1.zip › Figure 3-source data 1/121-1.tif]

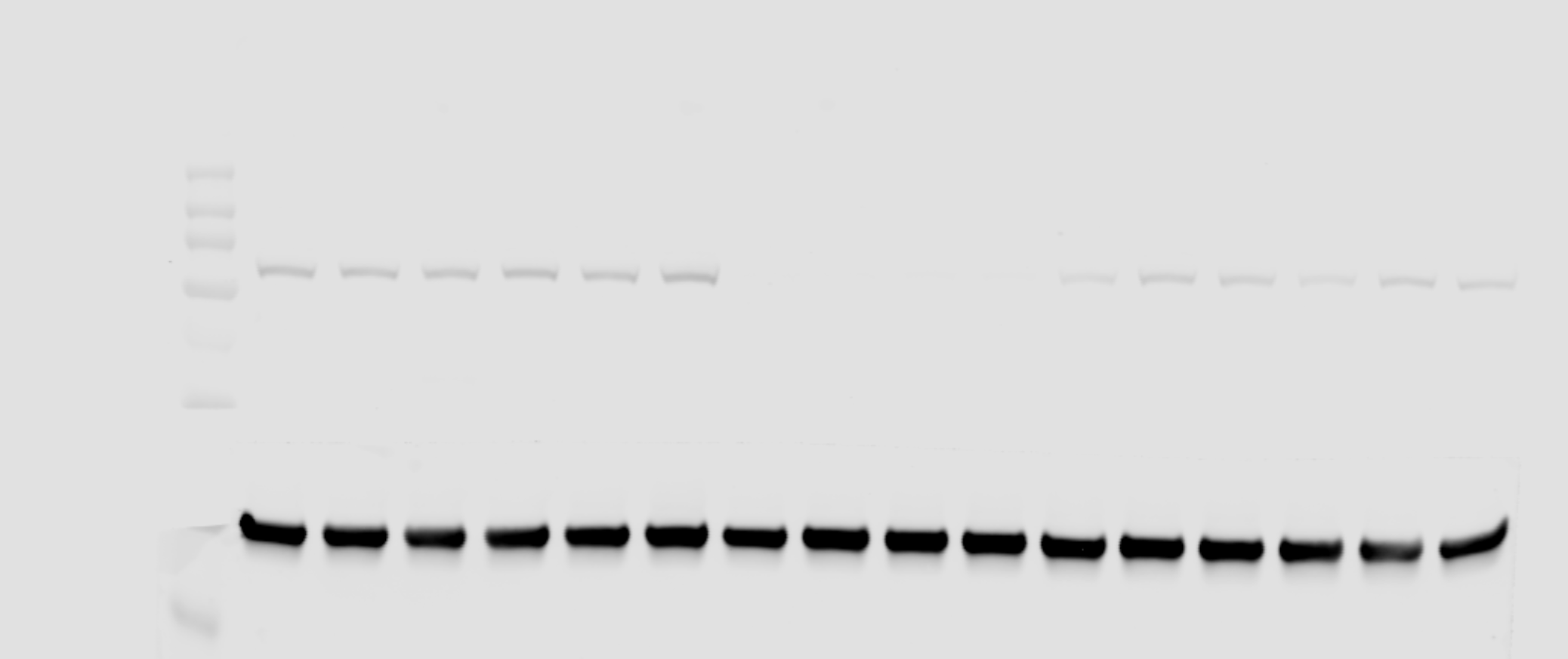

Supplement: Figure 3—source data 1. — The dashed boxes indicate the areas of blots presented in the figure. [file elife-81892-fig3-data1.zip › Figure 3-source data 1/166-1.tif]

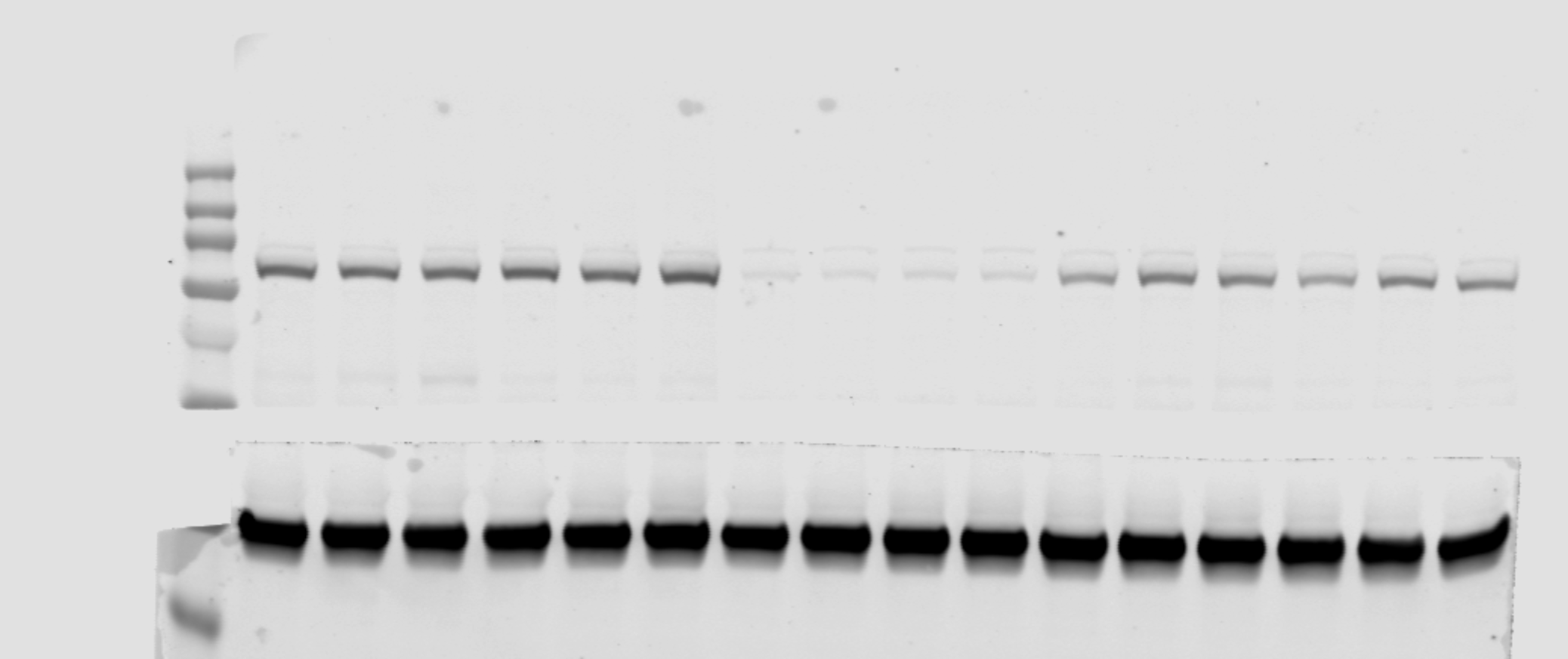

Supplement: Figure 3—source data 1. — The dashed boxes indicate the areas of blots presented in the figure. [file elife-81892-fig3-data1.zip › Figure 3-source data 1/166-2.tif]

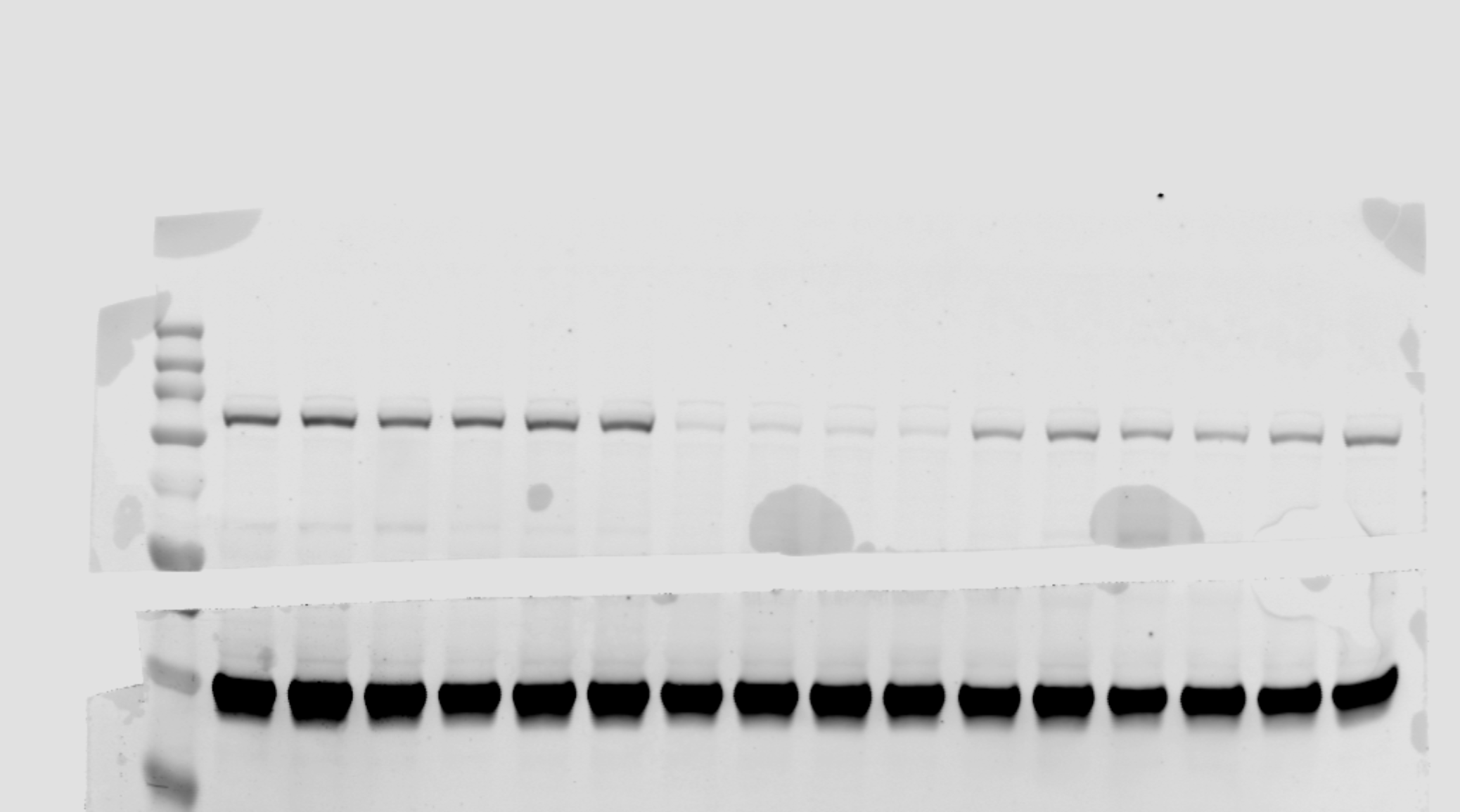

Supplement: Figure 3—source data 1. — The dashed boxes indicate the areas of blots presented in the figure. [file elife-81892-fig3-data1.zip › Figure 3-source data 1/170-1.tif]

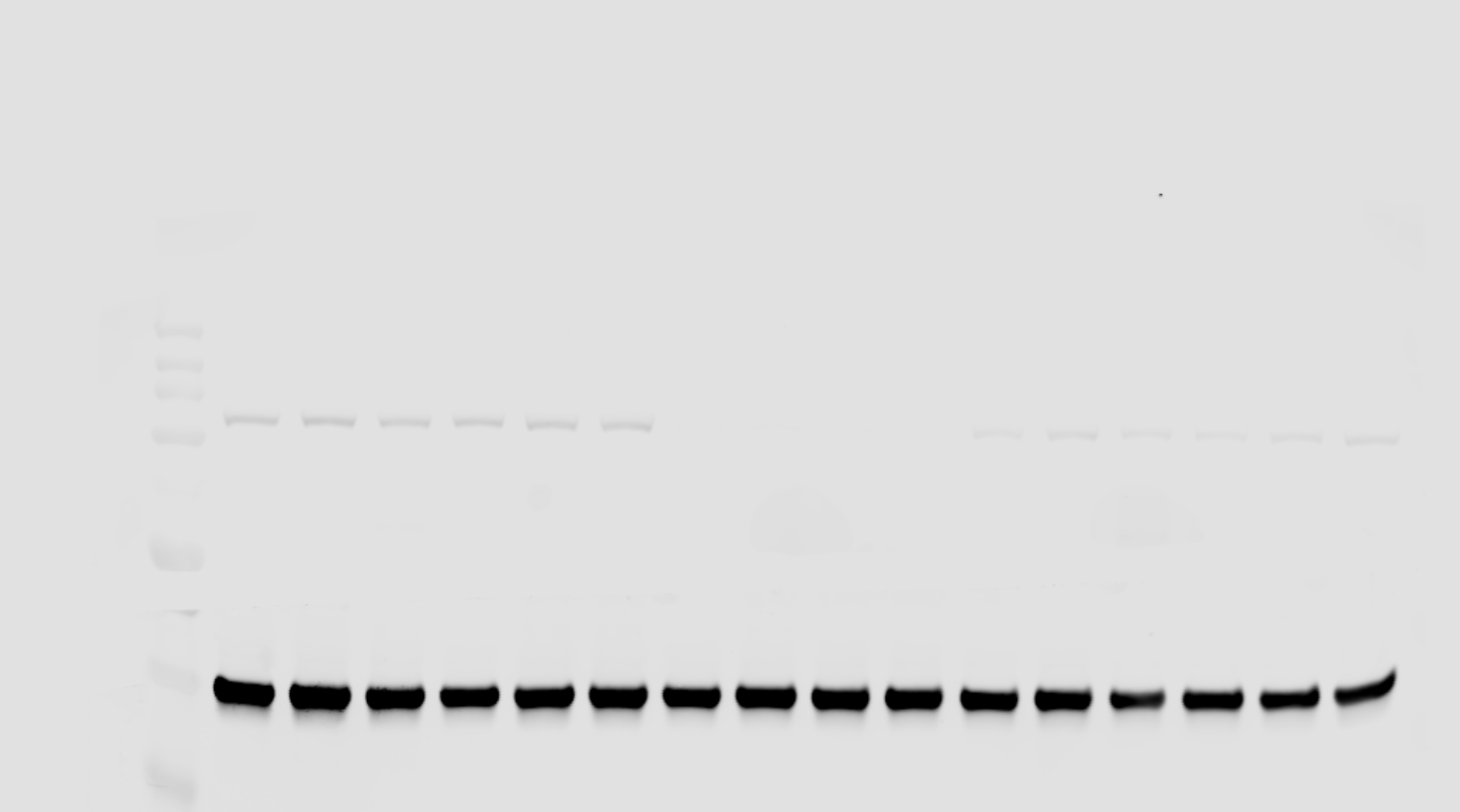

Supplement: Figure 3—source data 1. — The dashed boxes indicate the areas of blots presented in the figure. [file elife-81892-fig3-data1.zip › Figure 3-source data 1/170-2.tif]

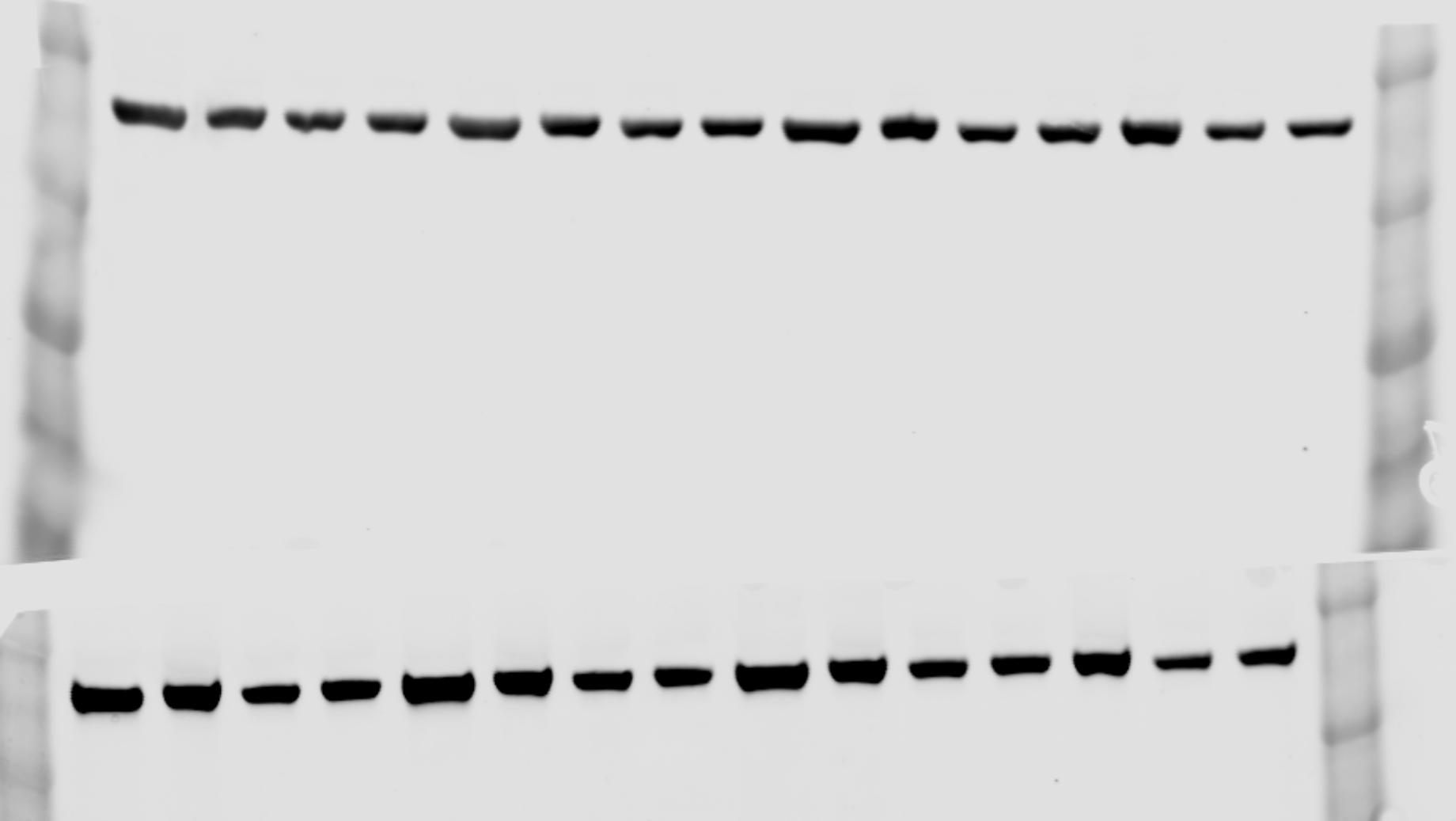

Supplement: Figure 3—source data 1. — The dashed boxes indicate the areas of blots presented in the figure. [file elife-81892-fig3-data1.zip › Figure 3-source data 1/211-1.tif]

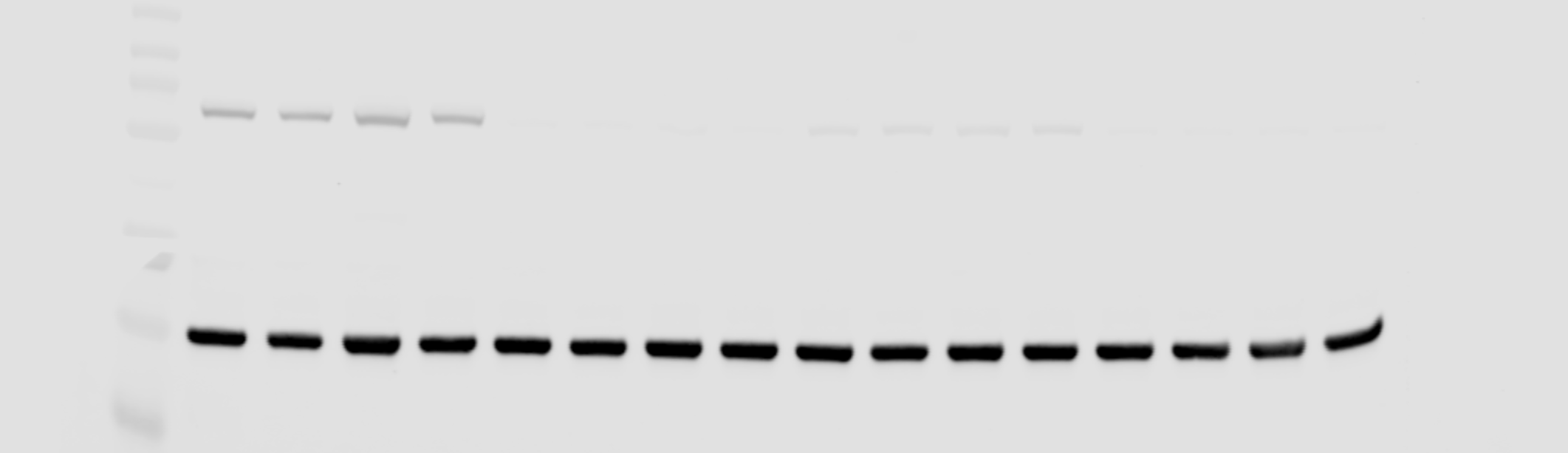

Supplement: Figure 3—source data 2. — The dashed boxes indicate the areas of blots presented in the figure. [file elife-81892-fig3-data2.zip › Figure 3-source data 2/215-1.tif]

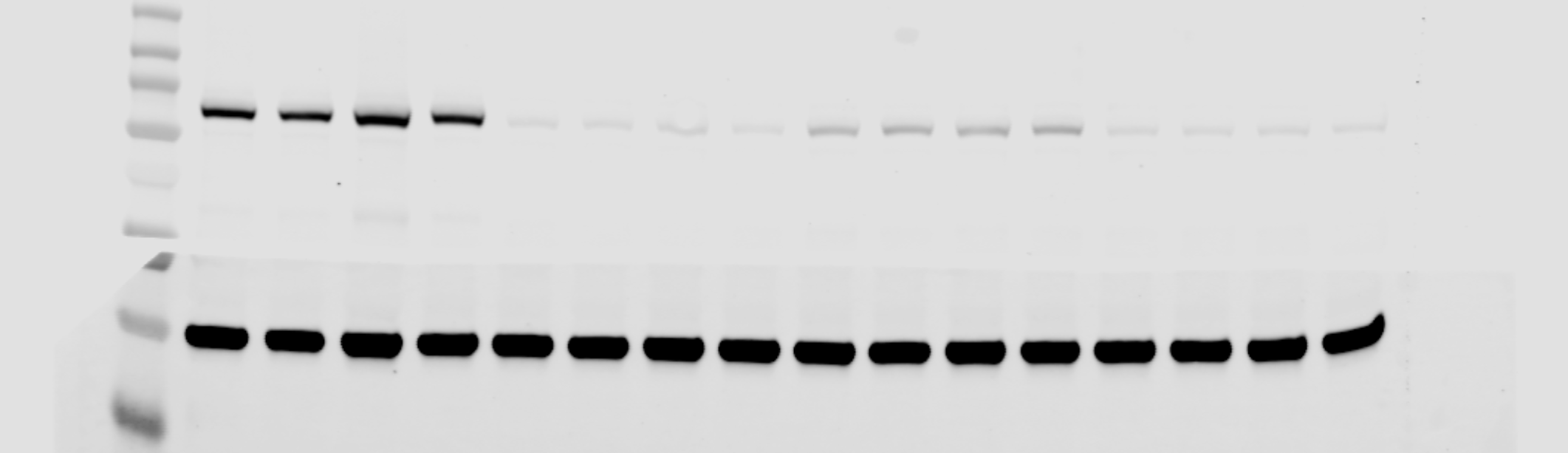

Supplement: Figure 3—source data 2. — The dashed boxes indicate the areas of blots presented in the figure. [file elife-81892-fig3-data2.zip › Figure 3-source data 2/215-2.tif]

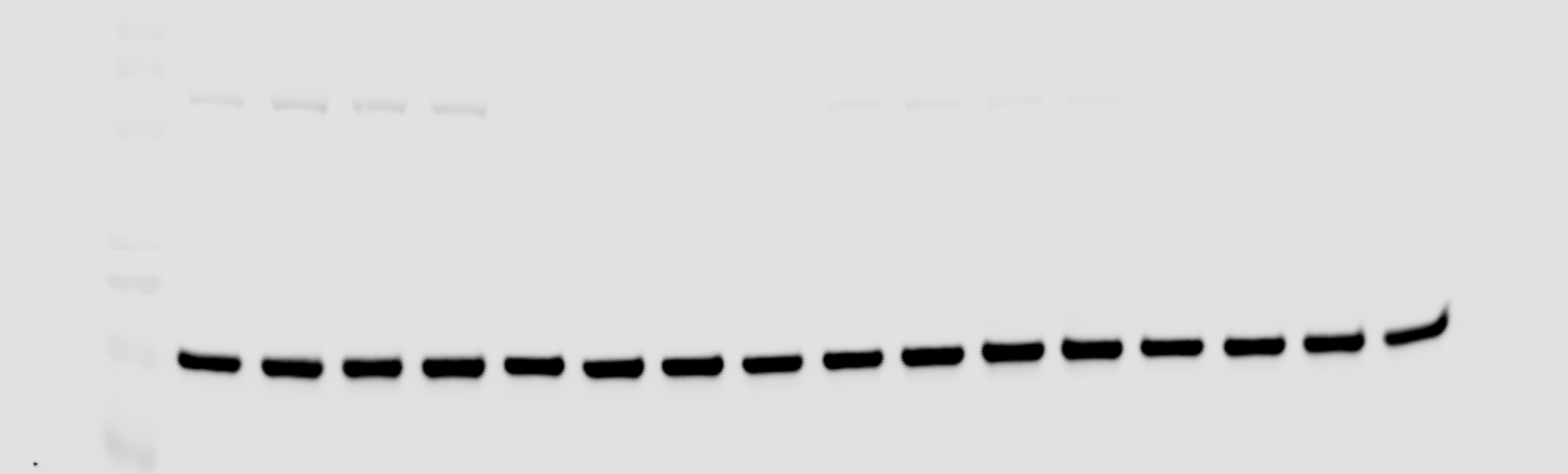

Supplement: Figure 3—source data 2. — The dashed boxes indicate the areas of blots presented in the figure. [file elife-81892-fig3-data2.zip › Figure 3-source data 2/224-1.tif]

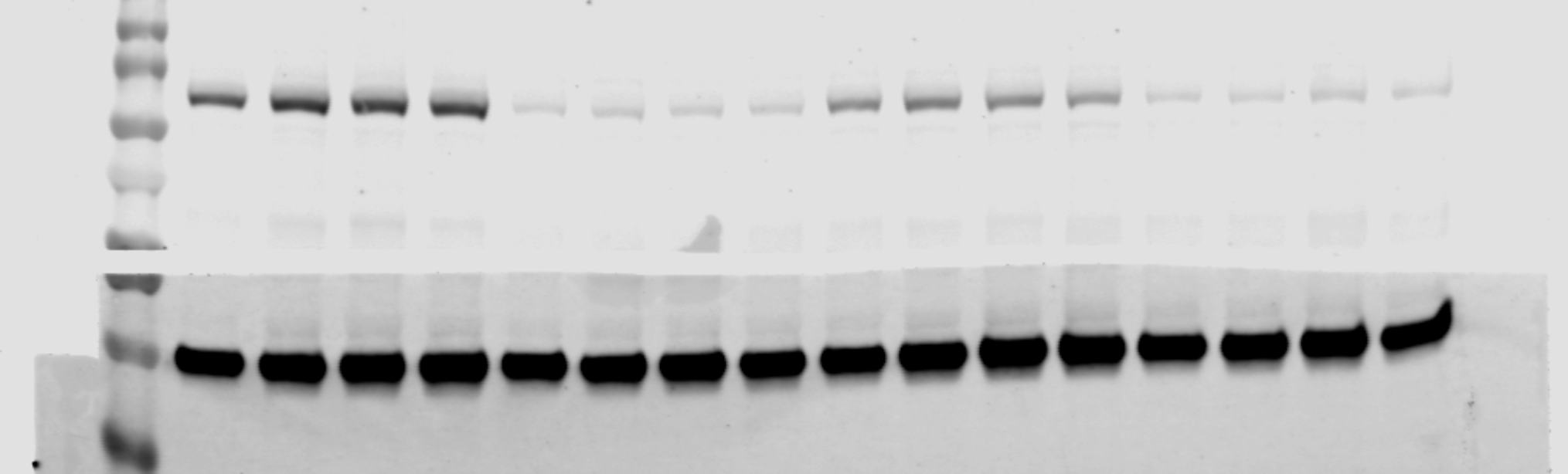

Supplement: Figure 3—source data 2. — The dashed boxes indicate the areas of blots presented in the figure. [file elife-81892-fig3-data2.zip › Figure 3-source data 2/224-2.tif]

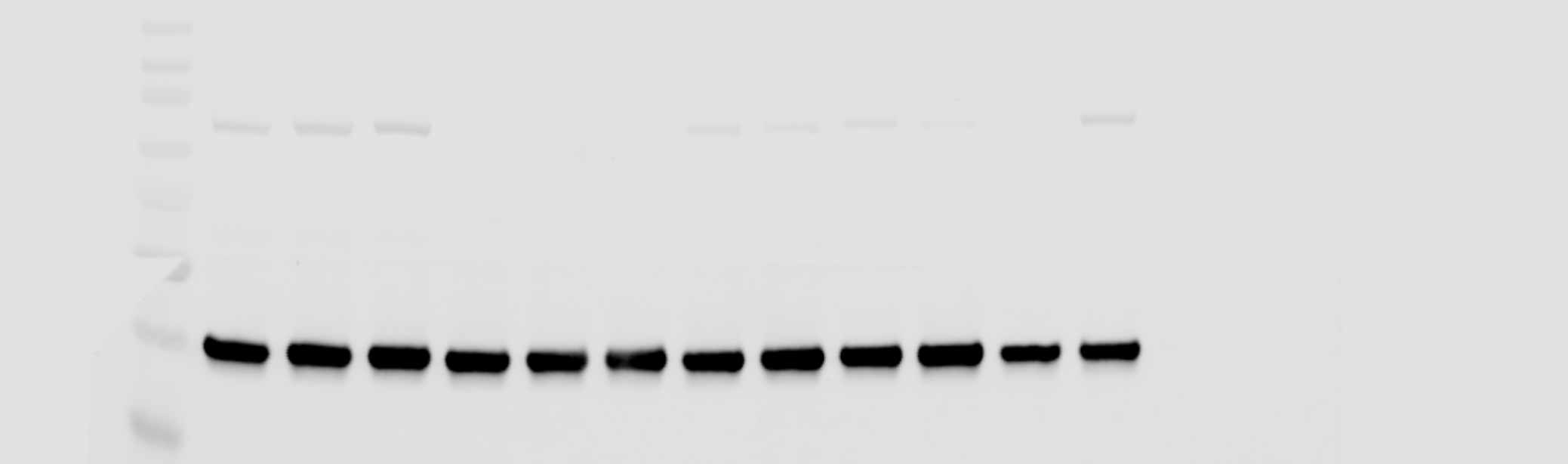

Supplement: Figure 3—source data 2. — The dashed boxes indicate the areas of blots presented in the figure. [file elife-81892-fig3-data2.zip › Figure 3-source data 2/238-1.tif]

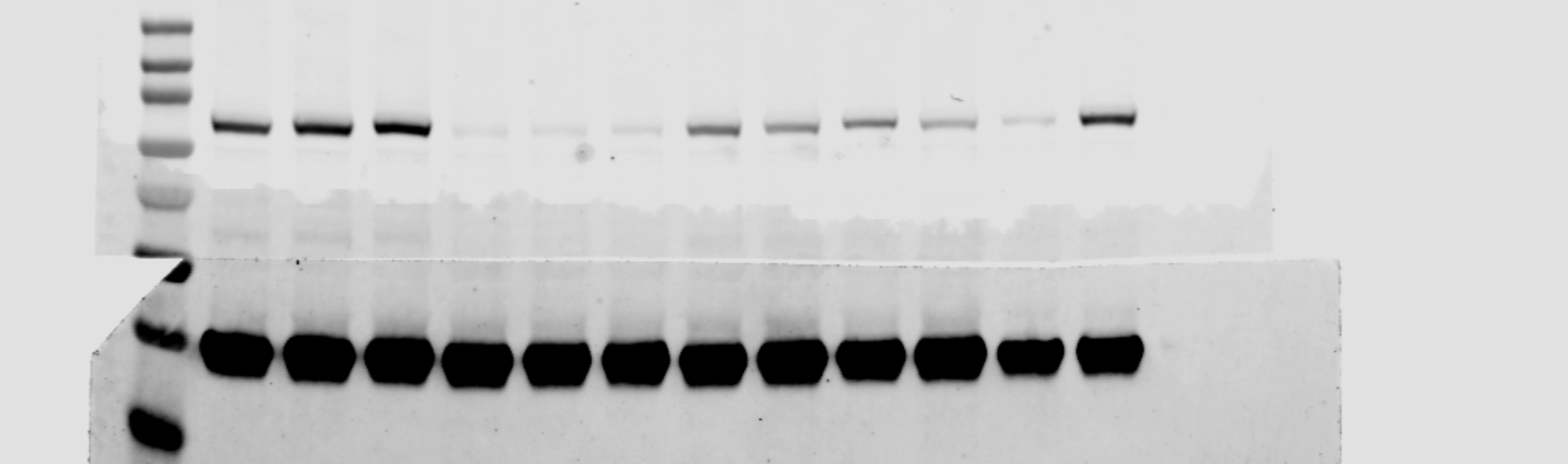

Supplement: Figure 3—source data 2. — The dashed boxes indicate the areas of blots presented in the figure. [file elife-81892-fig3-data2.zip › Figure 3-source data 2/238-2.tif]

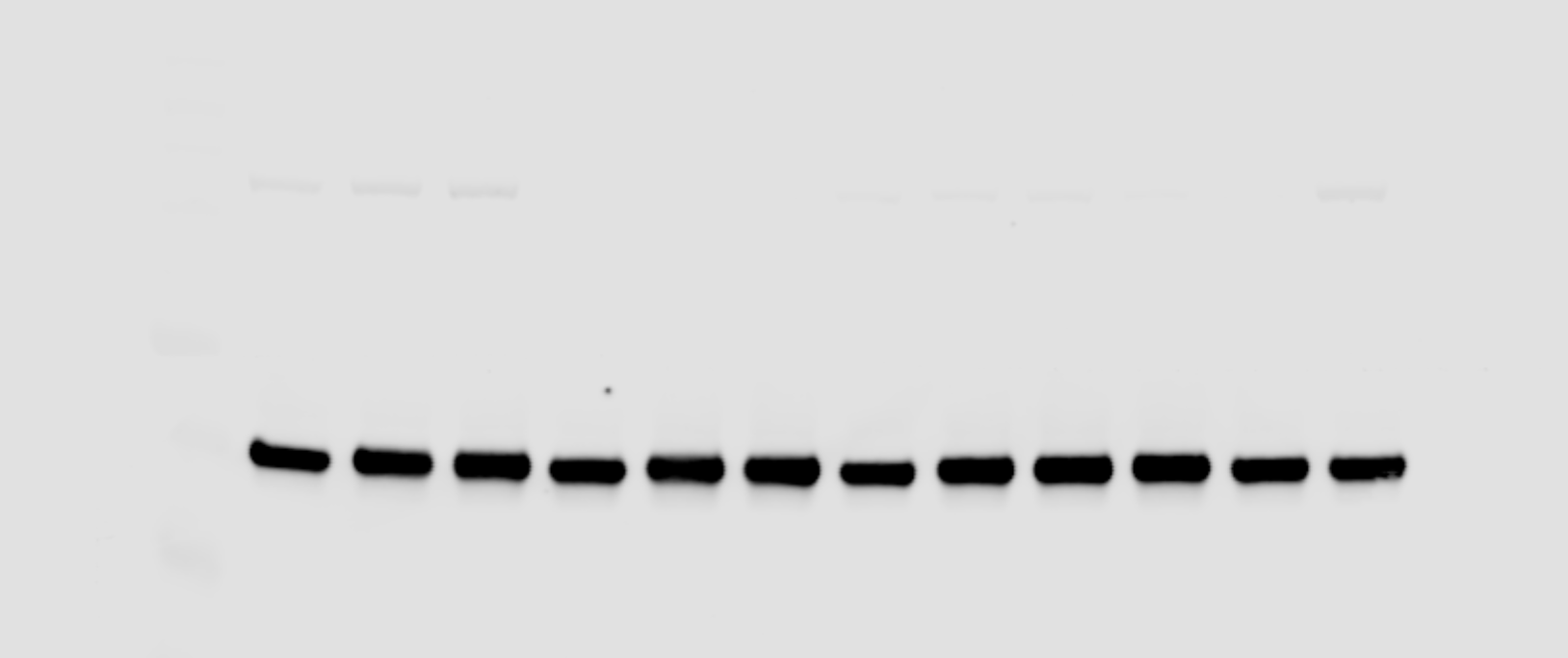

Supplement: Figure 3—source data 2. — The dashed boxes indicate the areas of blots presented in the figure. [file elife-81892-fig3-data2.zip › Figure 3-source data 2/242-1.tif]

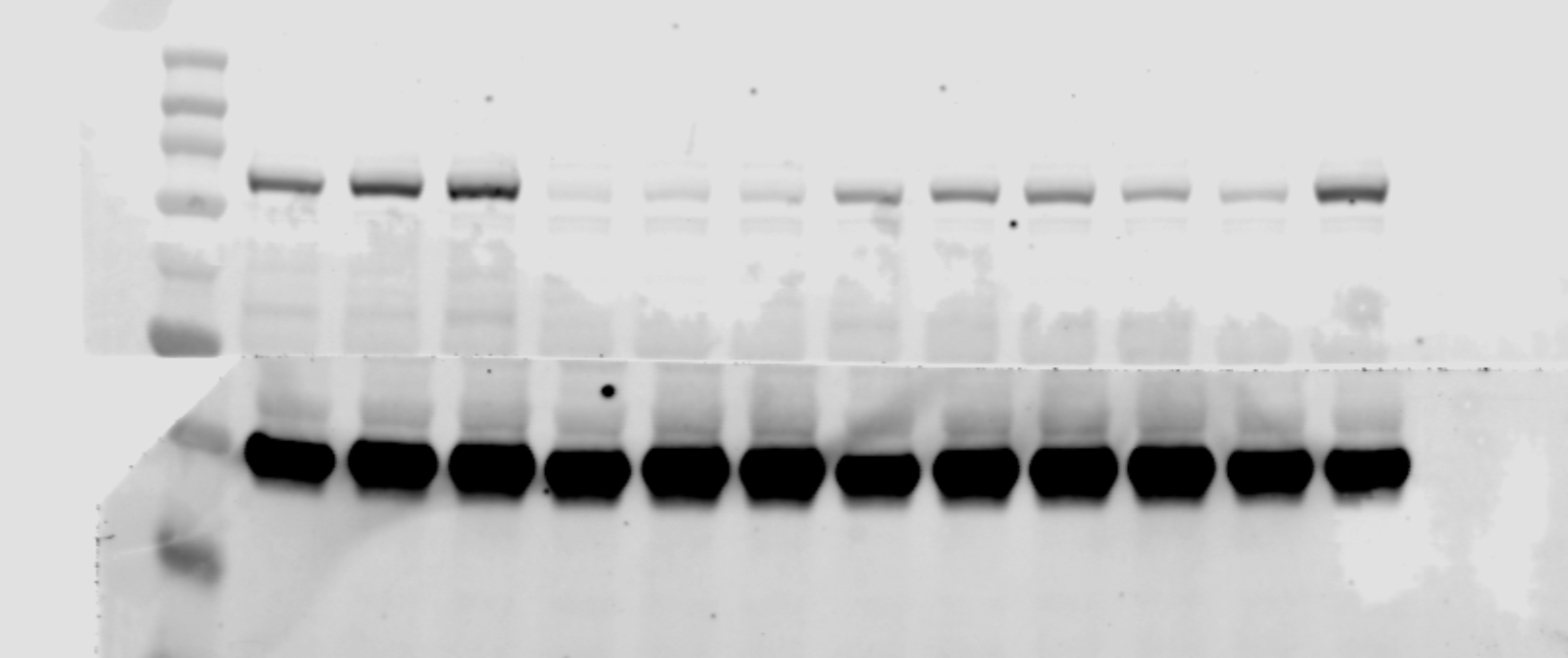

Supplement: Figure 3—source data 2. — The dashed boxes indicate the areas of blots presented in the figure. [file elife-81892-fig3-data2.zip › Figure 3-source data 2/242-2.tif]

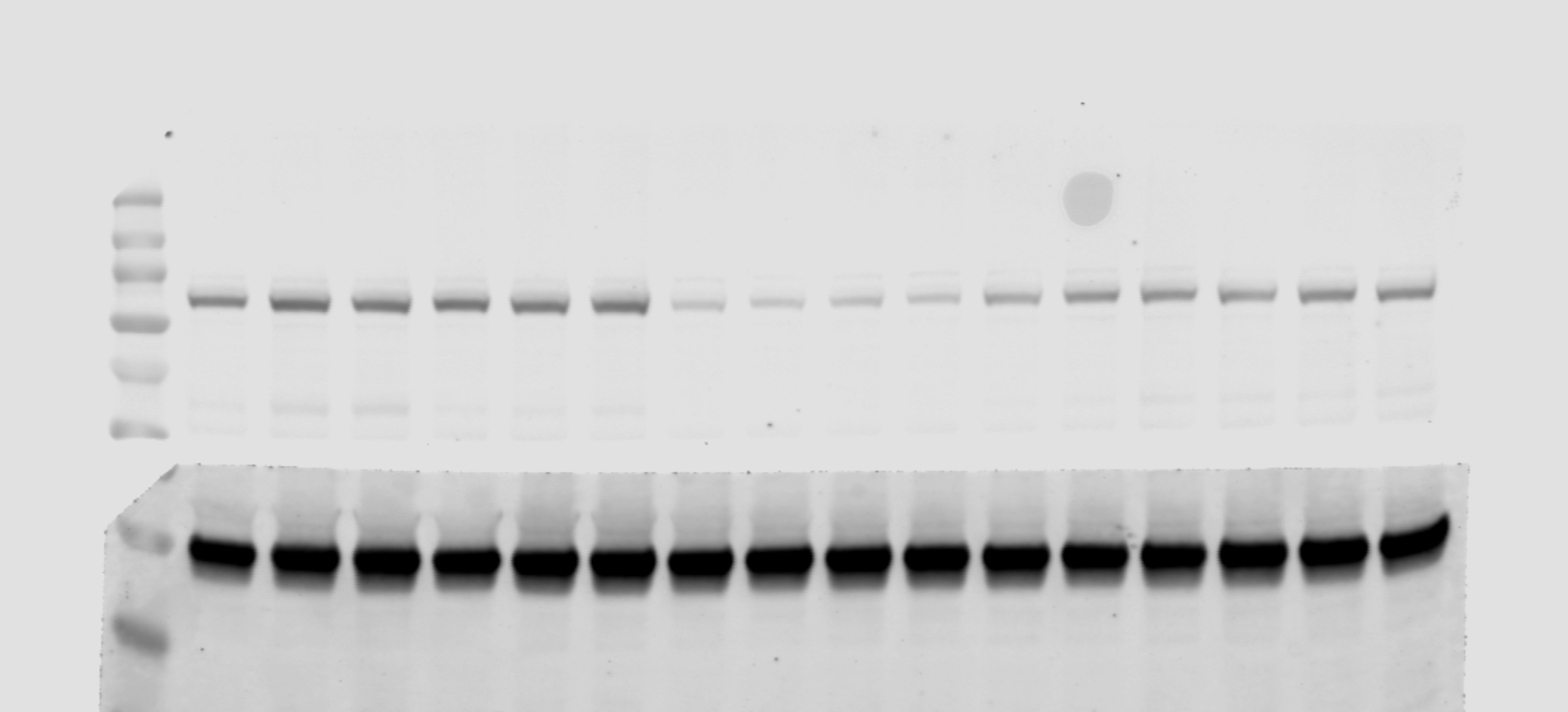

Supplement: Figure 3—figure supplement 1—source data 1. — The dashed boxes indicate the areas of blots presented in the figure. [file elife-81892-fig3-figsupp1-data1.zip › Figure 3-figure supplement 1-source data 1/162-1.tif]

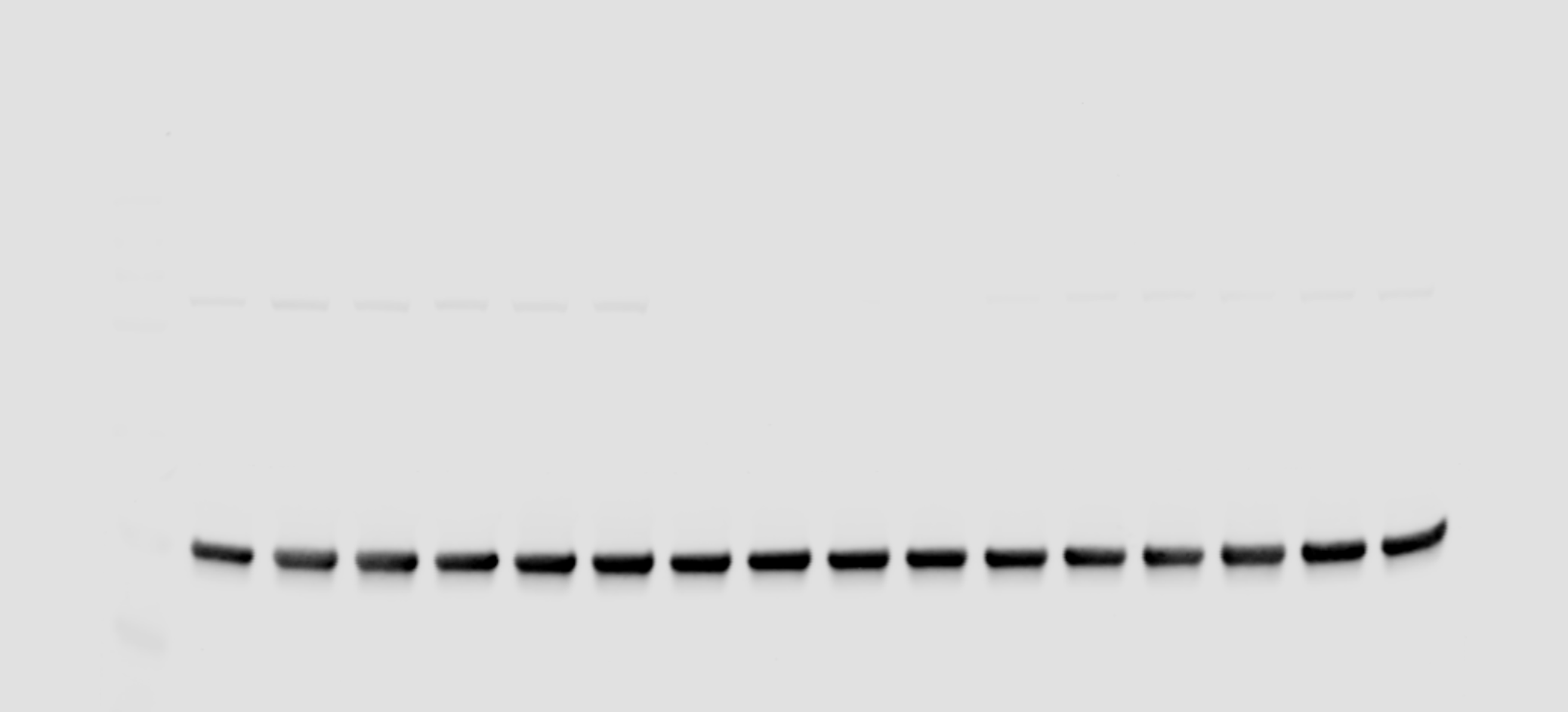

Supplement: Figure 3—figure supplement 1—source data 1. — The dashed boxes indicate the areas of blots presented in the figure. [file elife-81892-fig3-figsupp1-data1.zip › Figure 3-figure supplement 1-source data 1/162-2.tif]

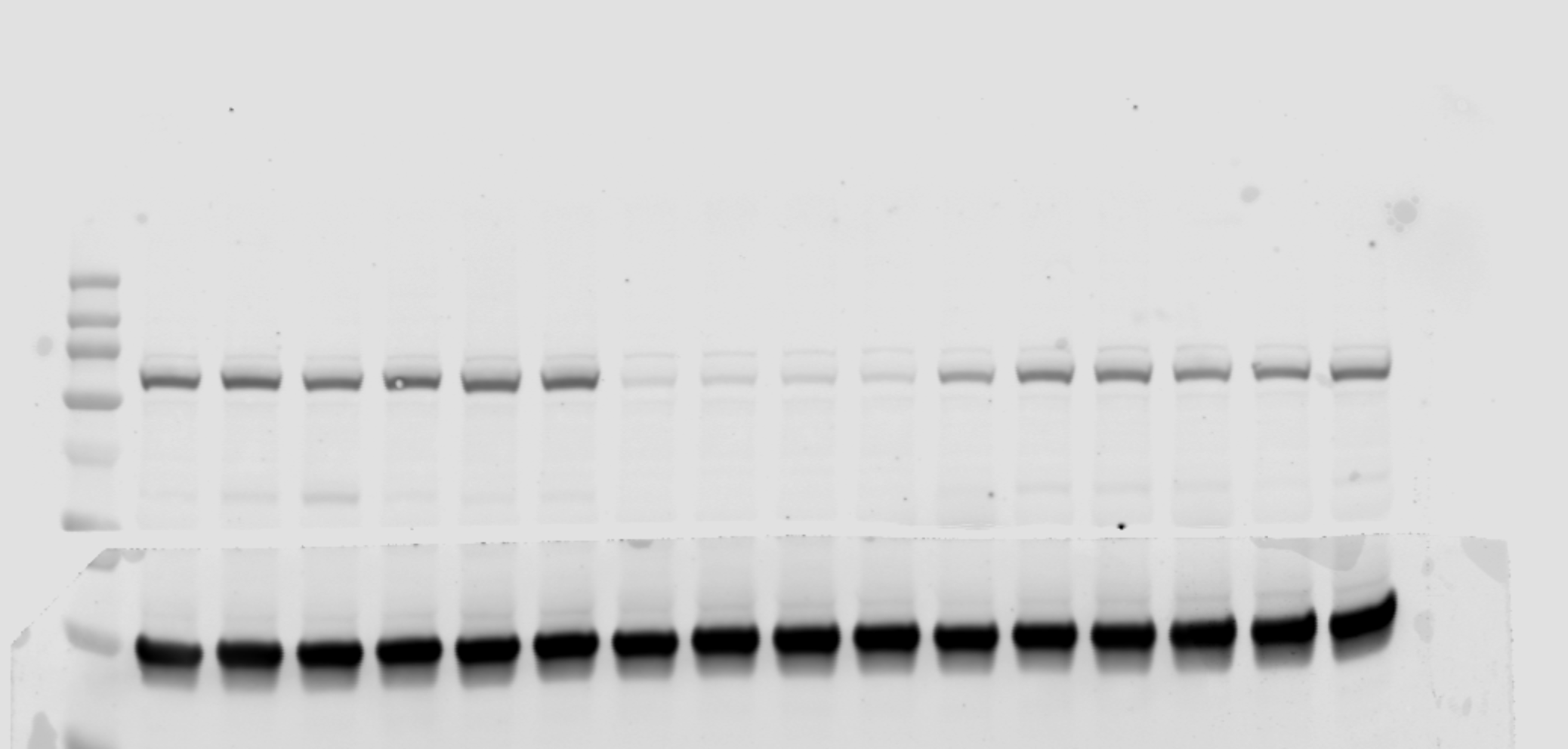

Supplement: Figure 3—figure supplement 1—source data 1. — The dashed boxes indicate the areas of blots presented in the figure. [file elife-81892-fig3-figsupp1-data1.zip › Figure 3-figure supplement 1-source data 1/168-1.tif]

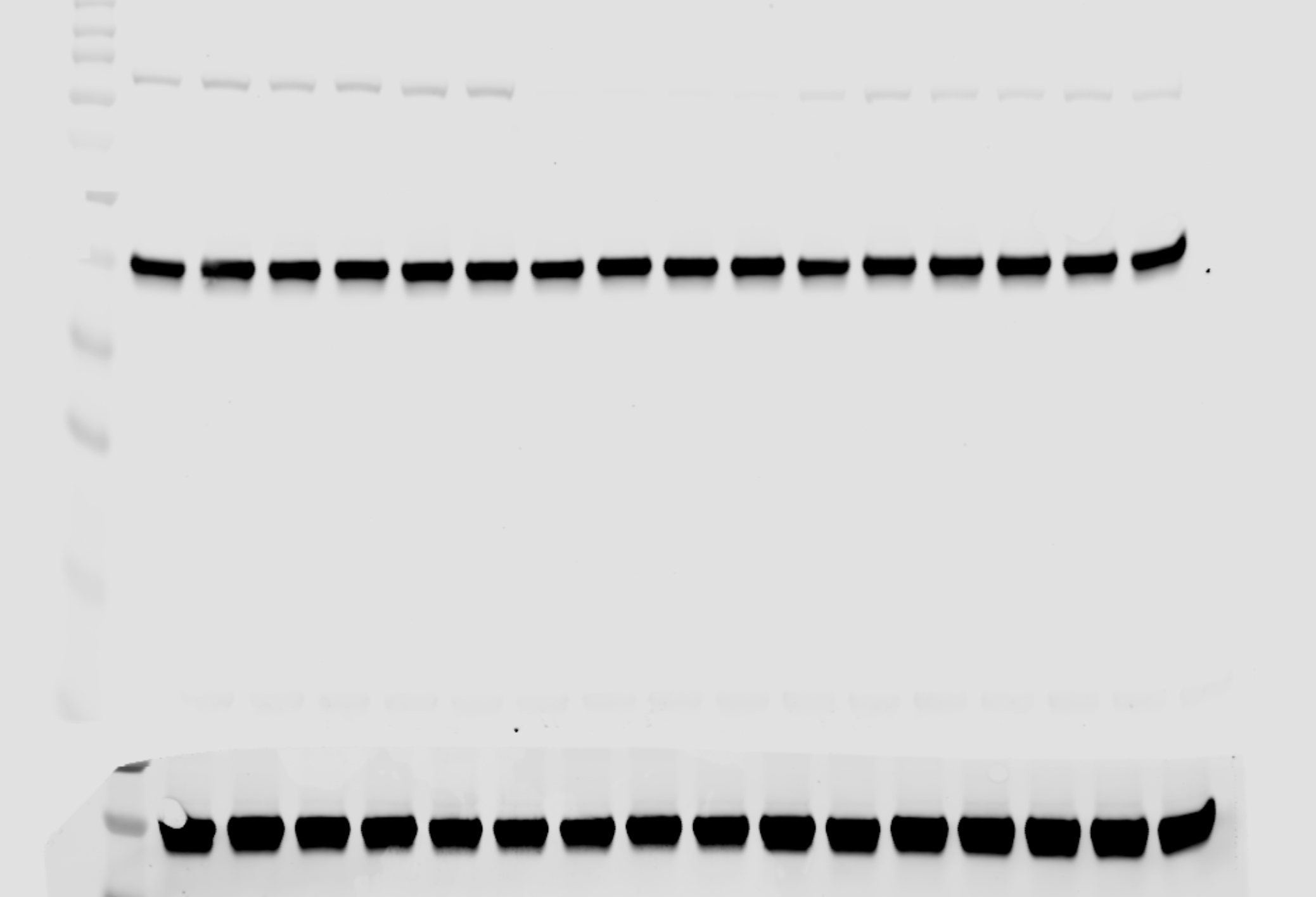

Supplement: Figure 3—figure supplement 1—source data 1. — The dashed boxes indicate the areas of blots presented in the figure. [file elife-81892-fig3-figsupp1-data1.zip › Figure 3-figure supplement 1-source data 1/174-1.tif]

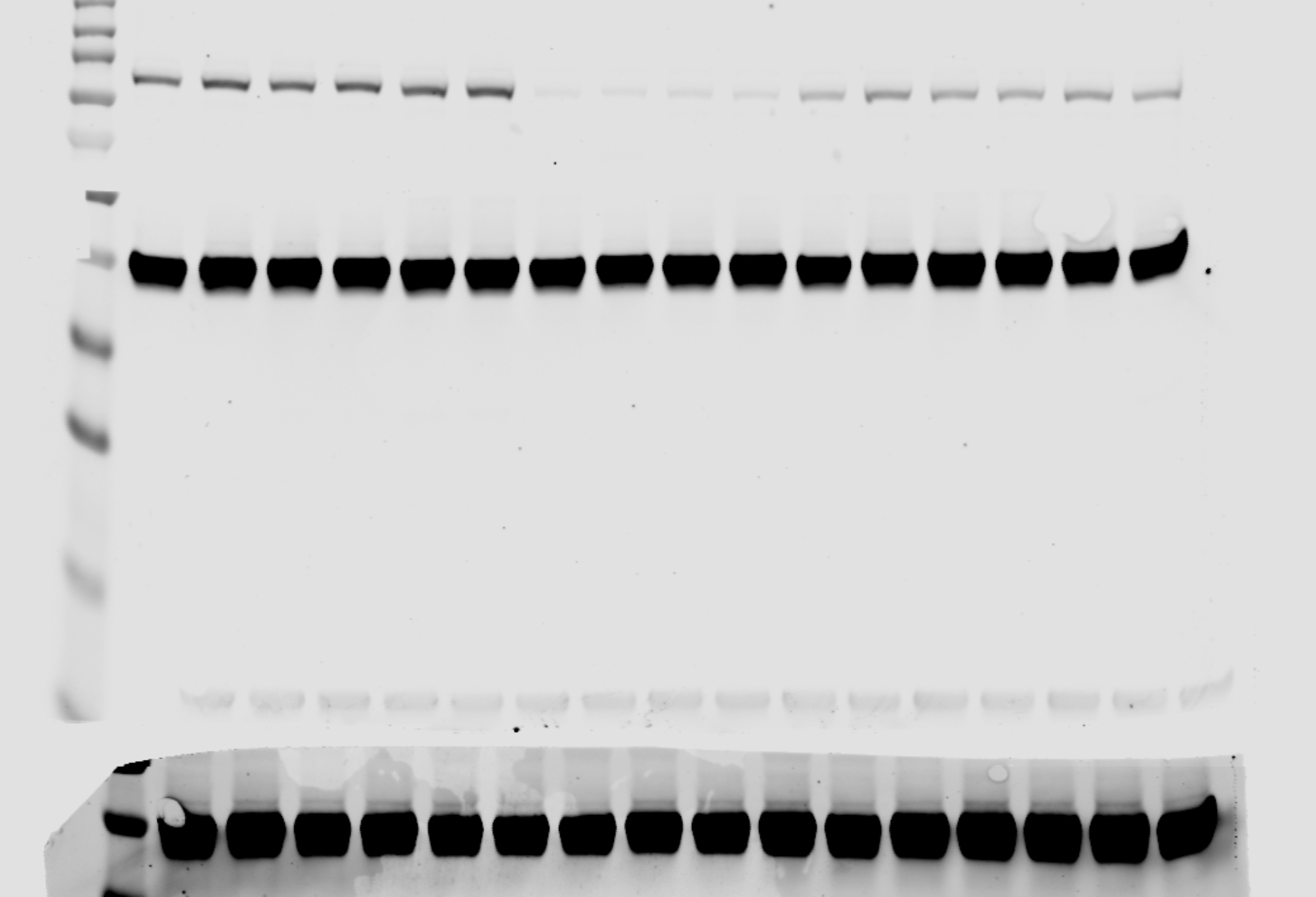

Supplement: Figure 3—figure supplement 1—source data 1. — The dashed boxes indicate the areas of blots presented in the figure. [file elife-81892-fig3-figsupp1-data1.zip › Figure 3-figure supplement 1-source data 1/174-2.tif]

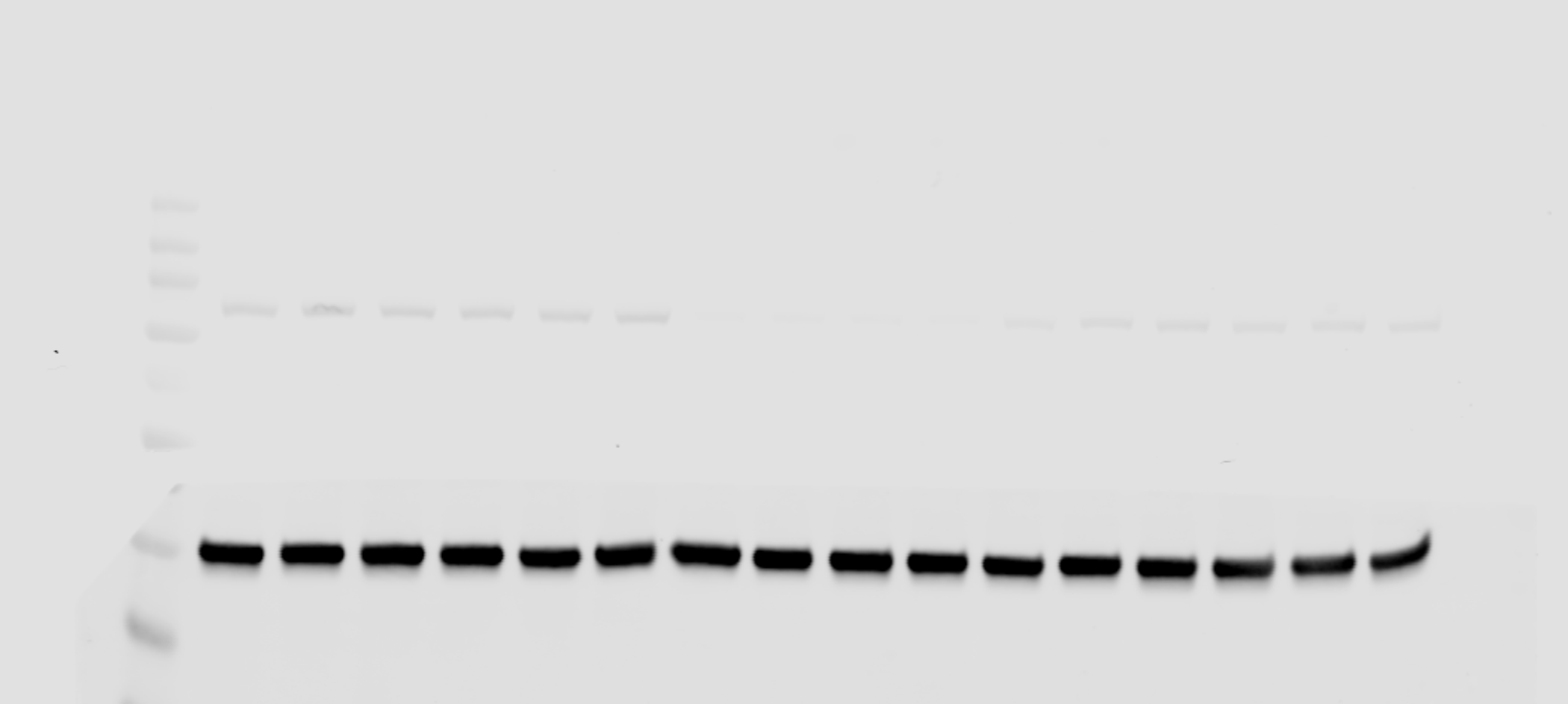

Supplement: Figure 3—figure supplement 1—source data 1. — The dashed boxes indicate the areas of blots presented in the figure. [file elife-81892-fig3-figsupp1-data1.zip › Figure 3-figure supplement 1-source data 1/179-1.tif]

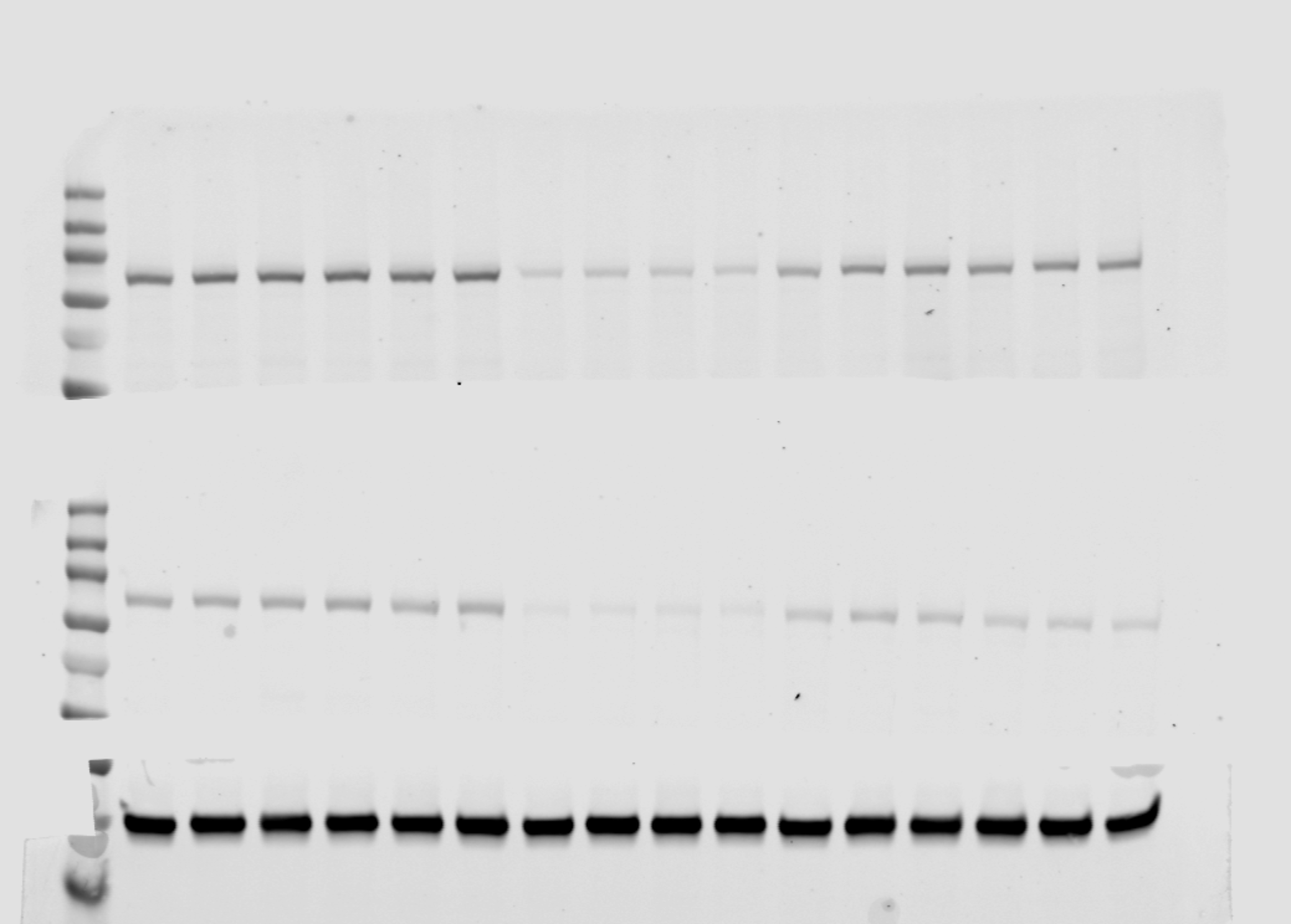

Supplement: Figure 3—figure supplement 1—source data 1. — The dashed boxes indicate the areas of blots presented in the figure. [file elife-81892-fig3-figsupp1-data1.zip › Figure 3-figure supplement 1-source data 1/182-1.tif]

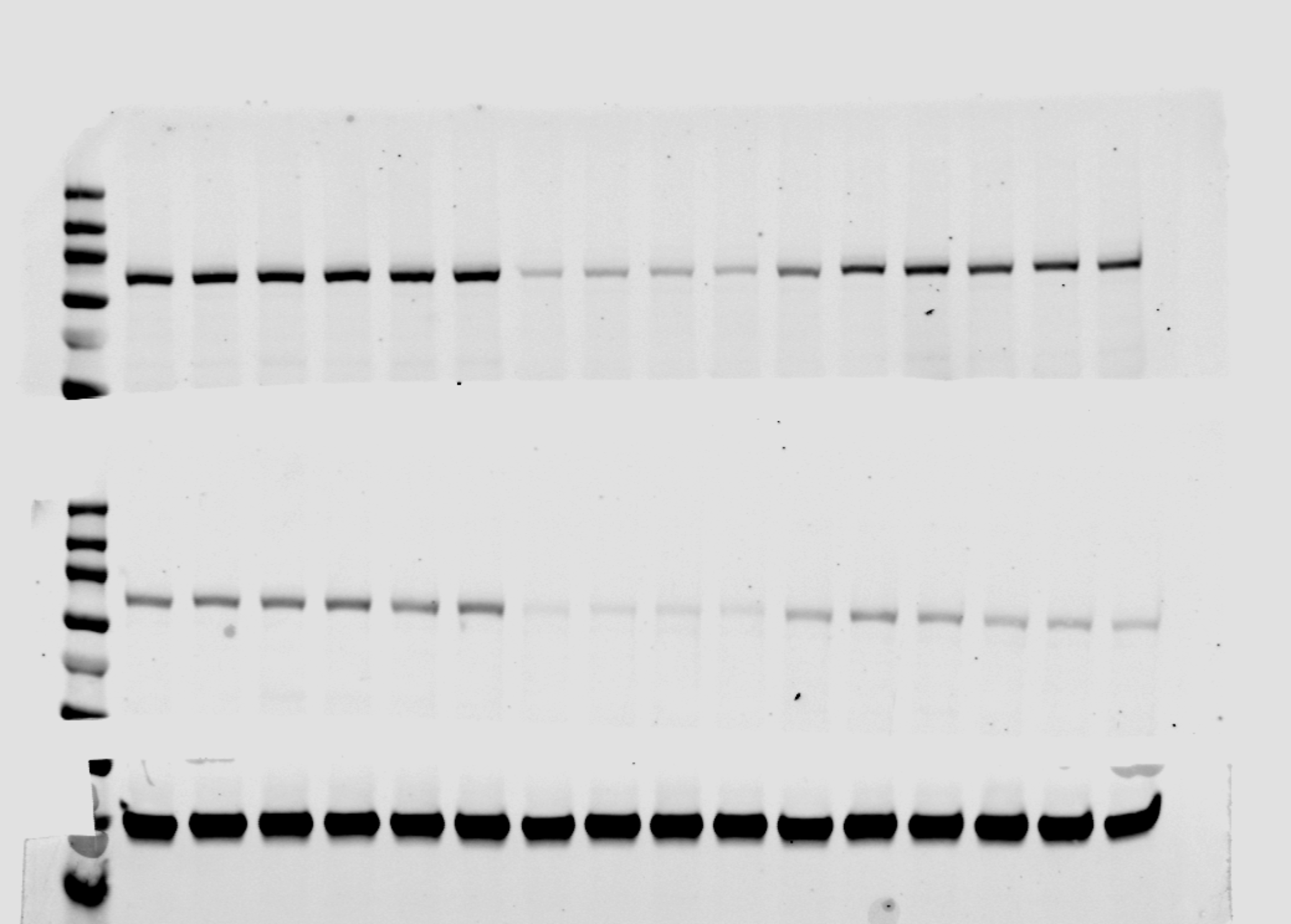

Supplement: Figure 3—figure supplement 1—source data 1. — The dashed boxes indicate the areas of blots presented in the figure. [file elife-81892-fig3-figsupp1-data1.zip › Figure 3-figure supplement 1-source data 1/182-2.tif]

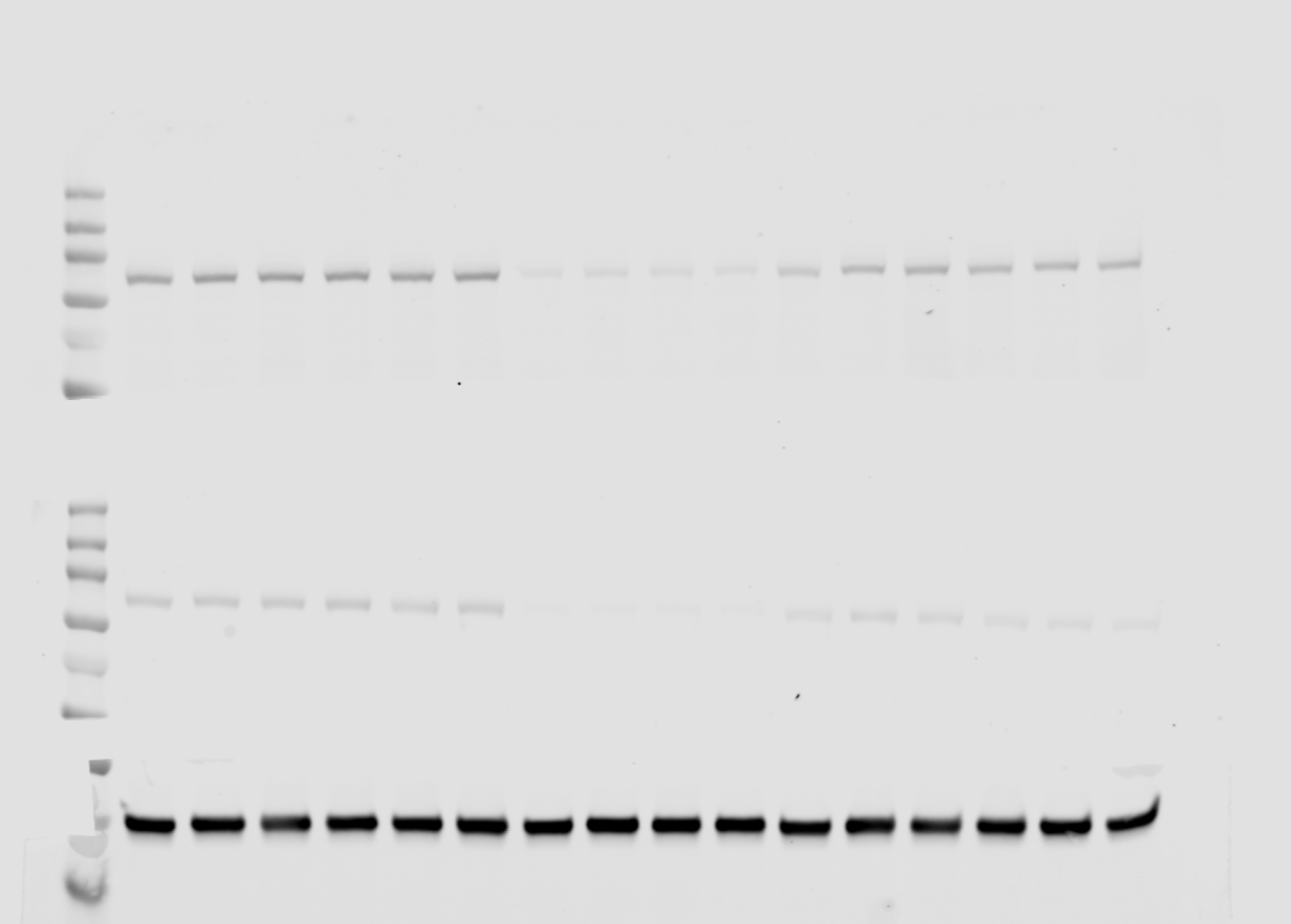

Supplement: Figure 3—figure supplement 1—source data 1. — The dashed boxes indicate the areas of blots presented in the figure. [file elife-81892-fig3-figsupp1-data1.zip › Figure 3-figure supplement 1-source data 1/182-3.tif]

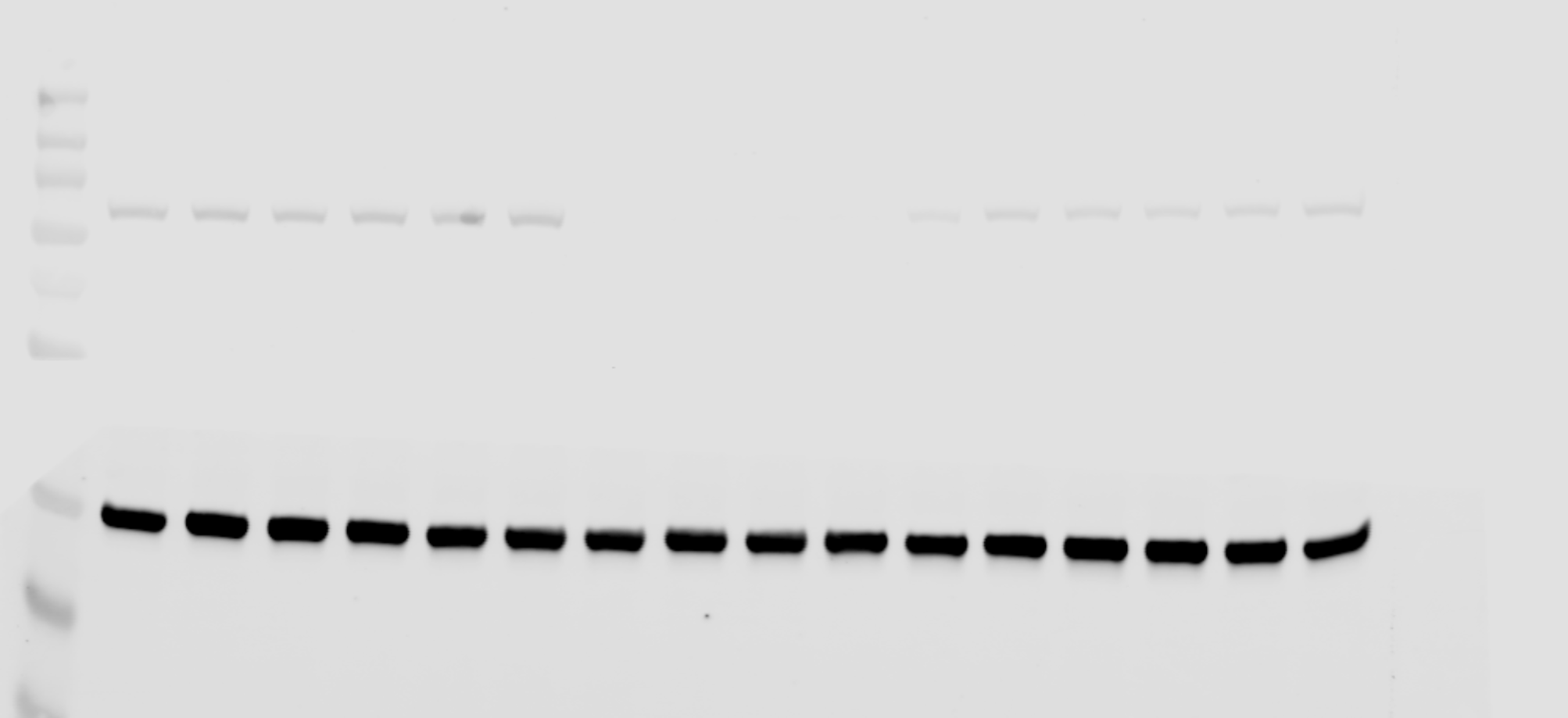

Supplement: Figure 3—figure supplement 1—source data 1. — The dashed boxes indicate the areas of blots presented in the figure. [file elife-81892-fig3-figsupp1-data1.zip › Figure 3-figure supplement 1-source data 1/204-1.tif]

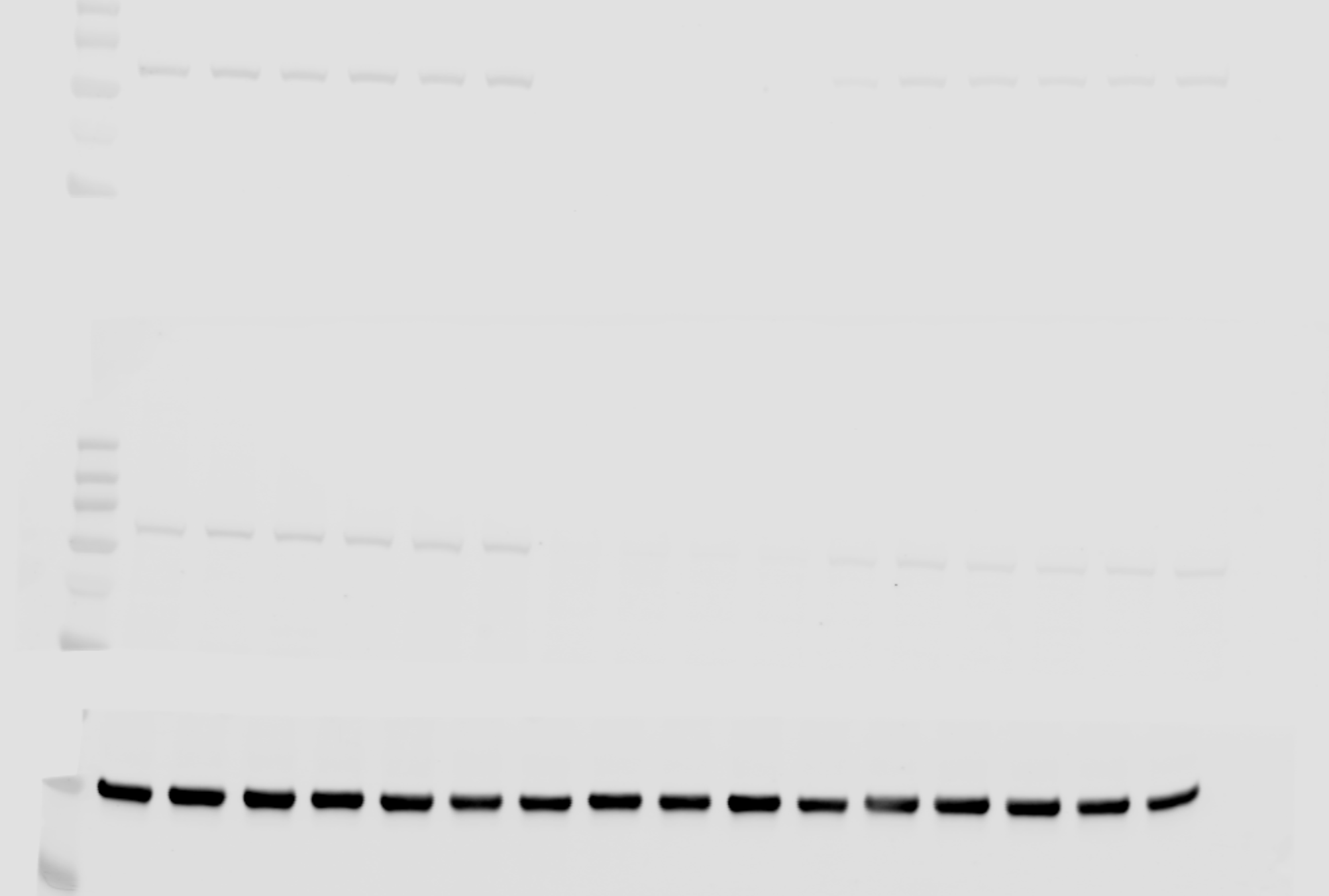

Supplement: Figure 3—figure supplement 1—source data 1. — The dashed boxes indicate the areas of blots presented in the figure. [file elife-81892-fig3-figsupp1-data1.zip › Figure 3-figure supplement 1-source data 1/206-1.tif]

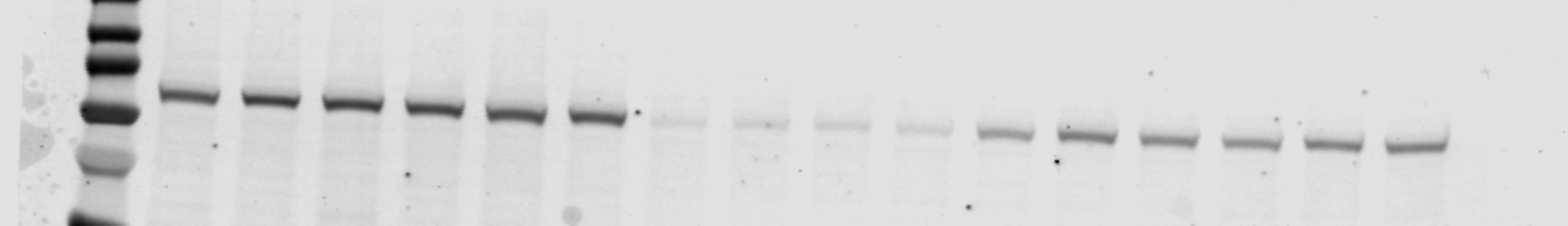

Supplement: Figure 3—figure supplement 1—source data 1. — The dashed boxes indicate the areas of blots presented in the figure. [file elife-81892-fig3-figsupp1-data1.zip › Figure 3-figure supplement 1-source data 1/208-1.tif]

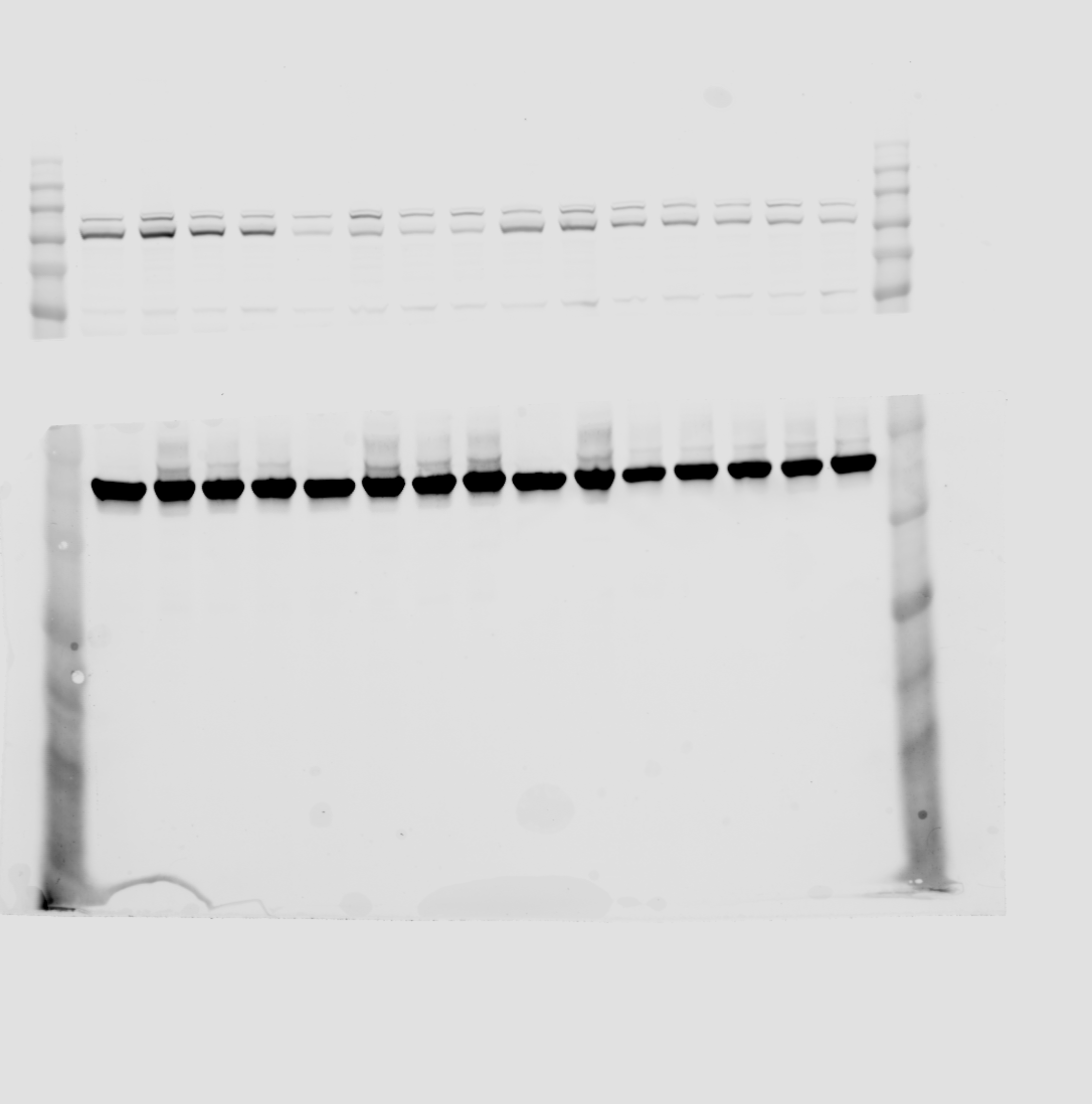

Supplement: Figure 3—figure supplement 1—source data 2. — The dashed boxes indicate the areas of blots presented in the figure. [file elife-81892-fig3-figsupp1-data2.zip › Figure 3-figure supplement 1-source data 2/115-1.tif]

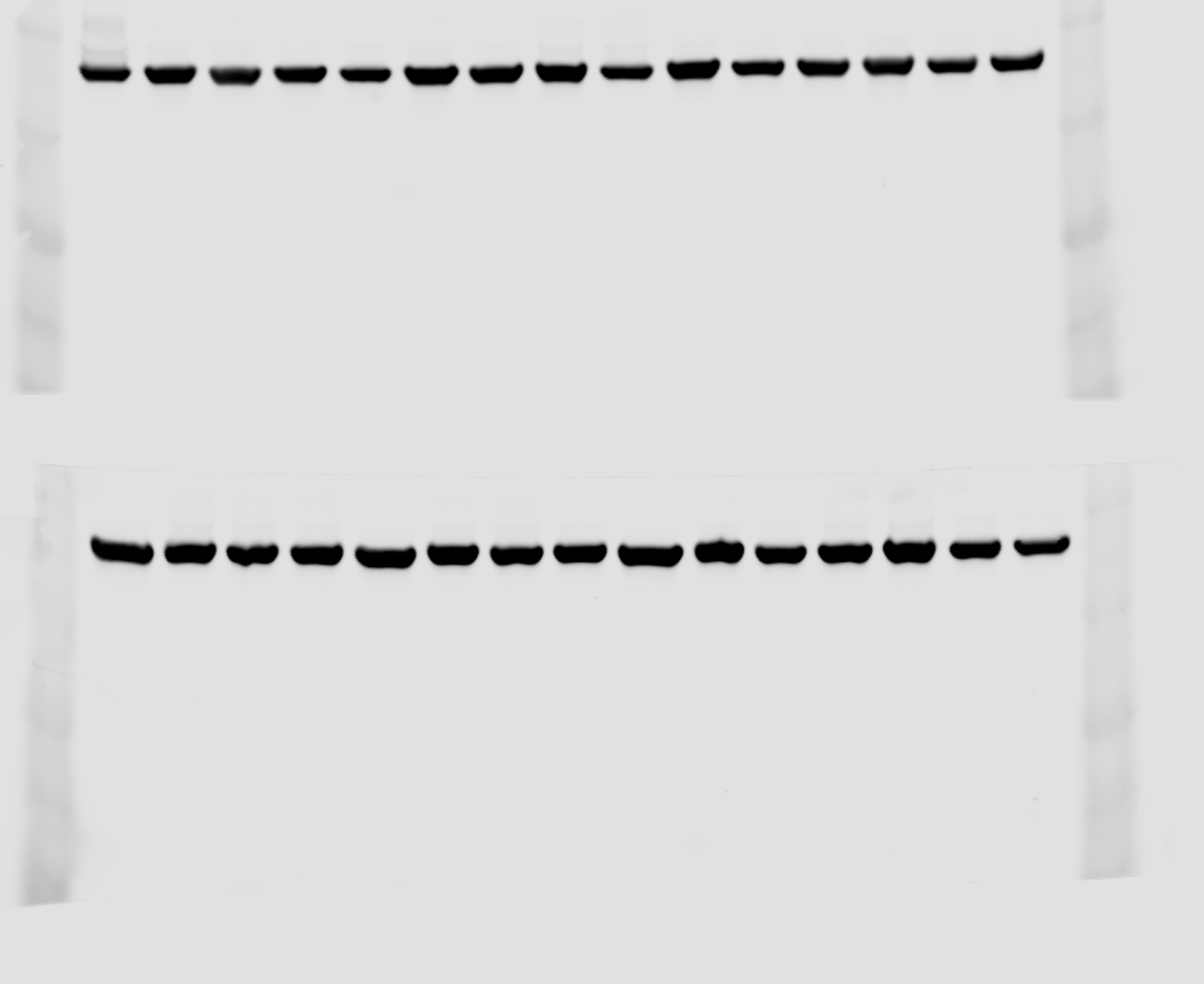

Supplement: Figure 3—figure supplement 1—source data 2. — The dashed boxes indicate the areas of blots presented in the figure. [file elife-81892-fig3-figsupp1-data2.zip › Figure 3-figure supplement 1-source data 2/119-1.tif]

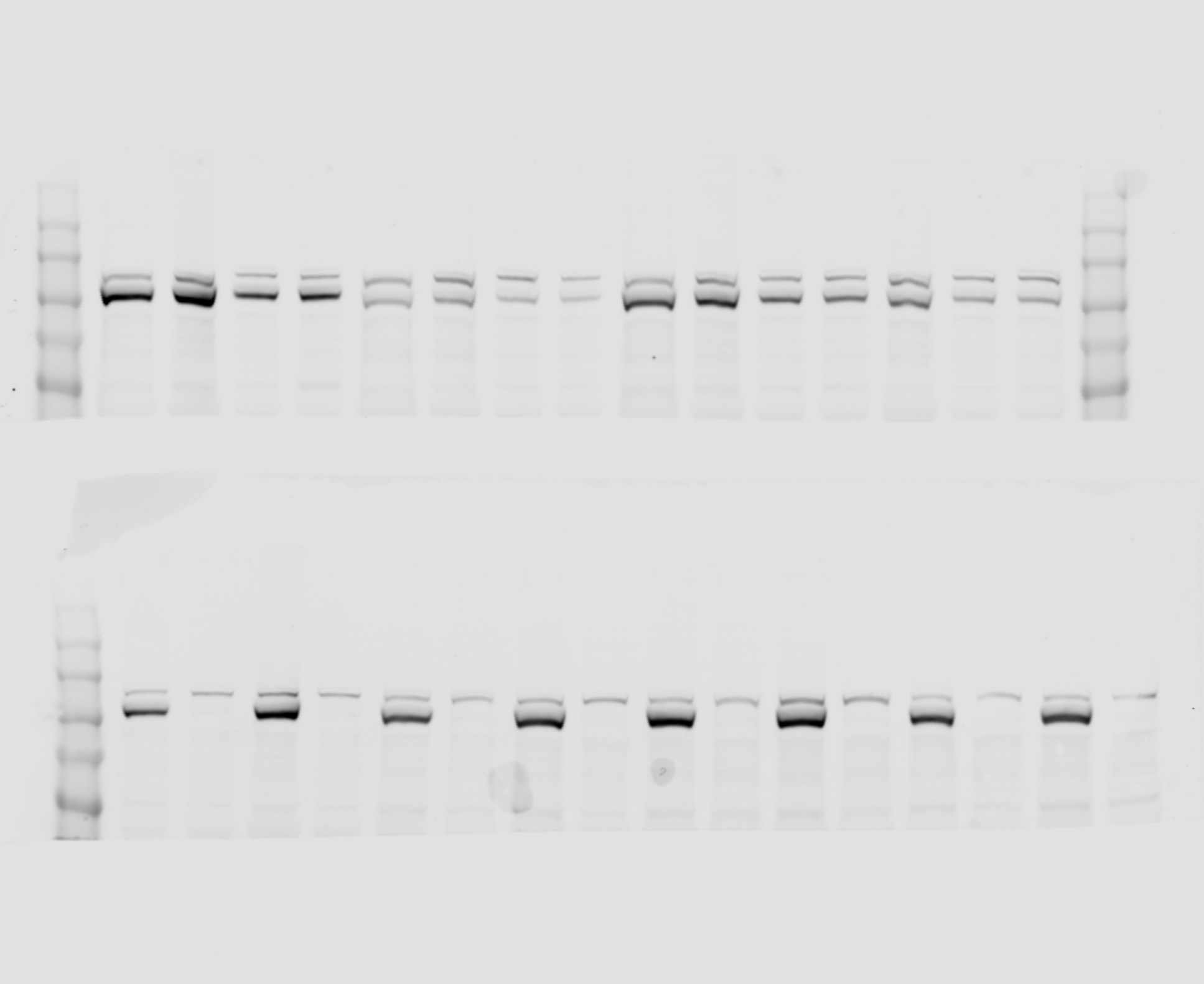

Supplement: Figure 3—figure supplement 1—source data 2. — The dashed boxes indicate the areas of blots presented in the figure. [file elife-81892-fig3-figsupp1-data2.zip › Figure 3-figure supplement 1-source data 2/130-1.tif]

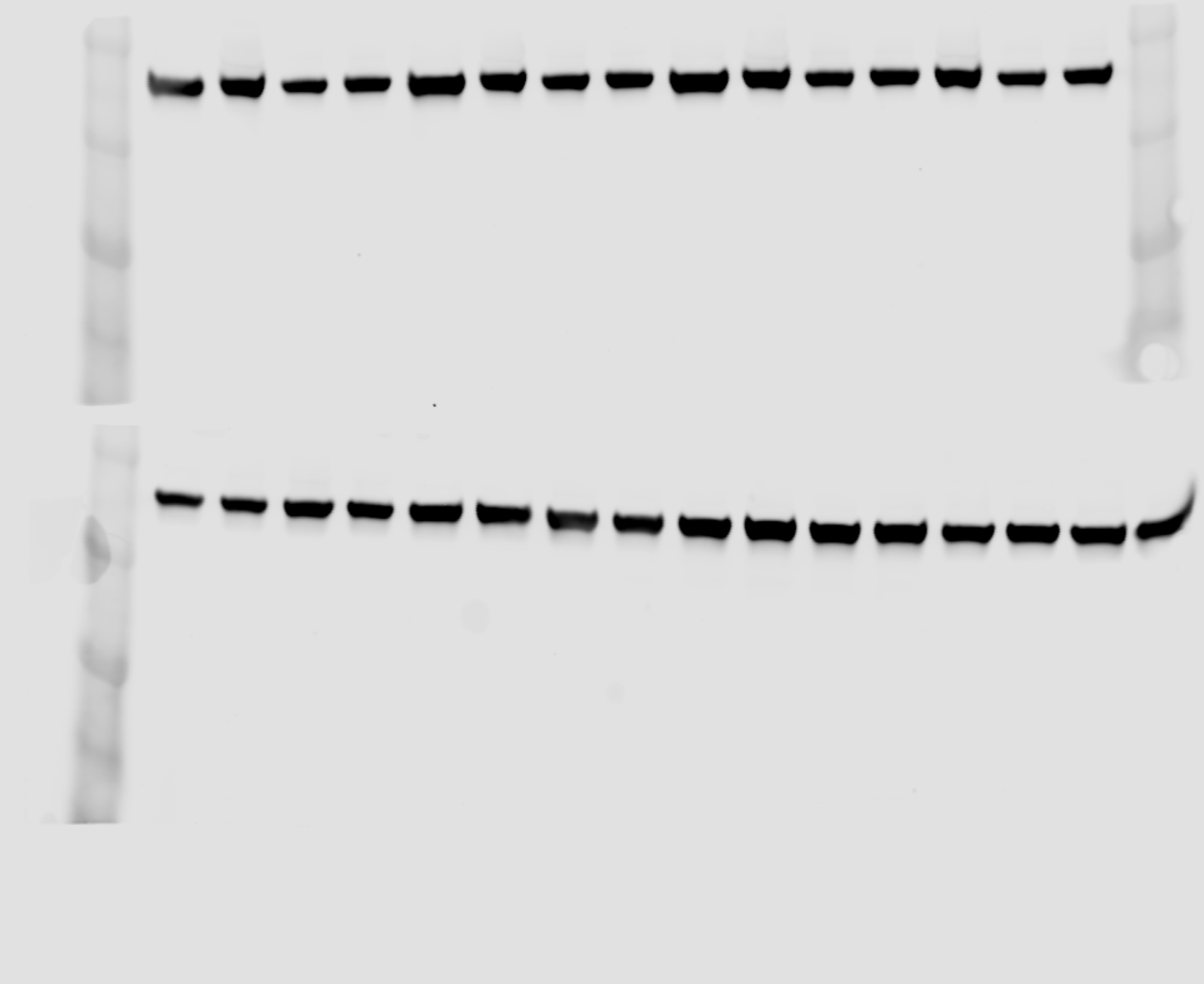

Supplement: Figure 3—figure supplement 1—source data 2. — The dashed boxes indicate the areas of blots presented in the figure. [file elife-81892-fig3-figsupp1-data2.zip › Figure 3-figure supplement 1-source data 2/132-1.tif]

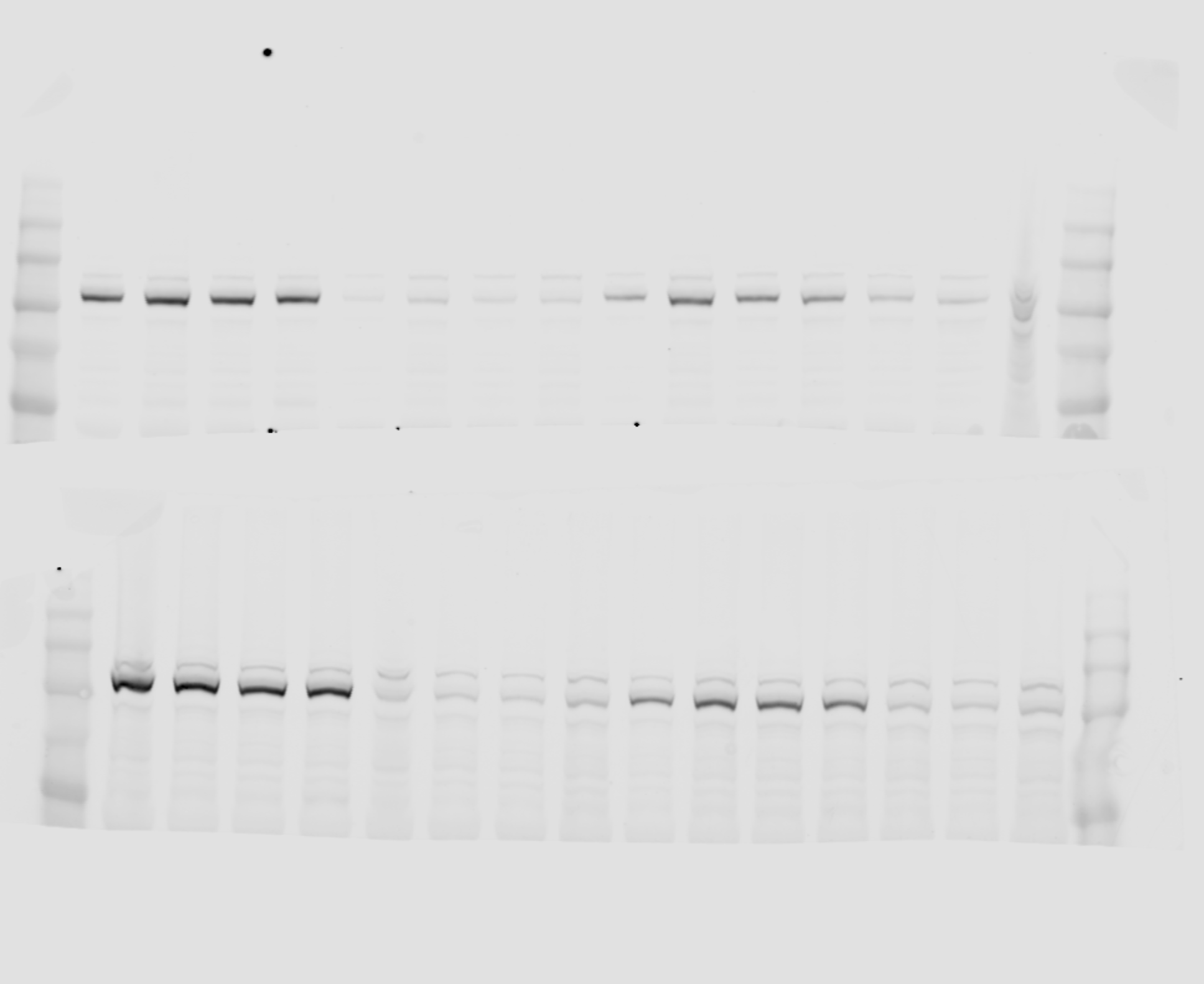

Supplement: Figure 3—figure supplement 1—source data 2. — The dashed boxes indicate the areas of blots presented in the figure. [file elife-81892-fig3-figsupp1-data2.zip › Figure 3-figure supplement 1-source data 2/134-1.tif]

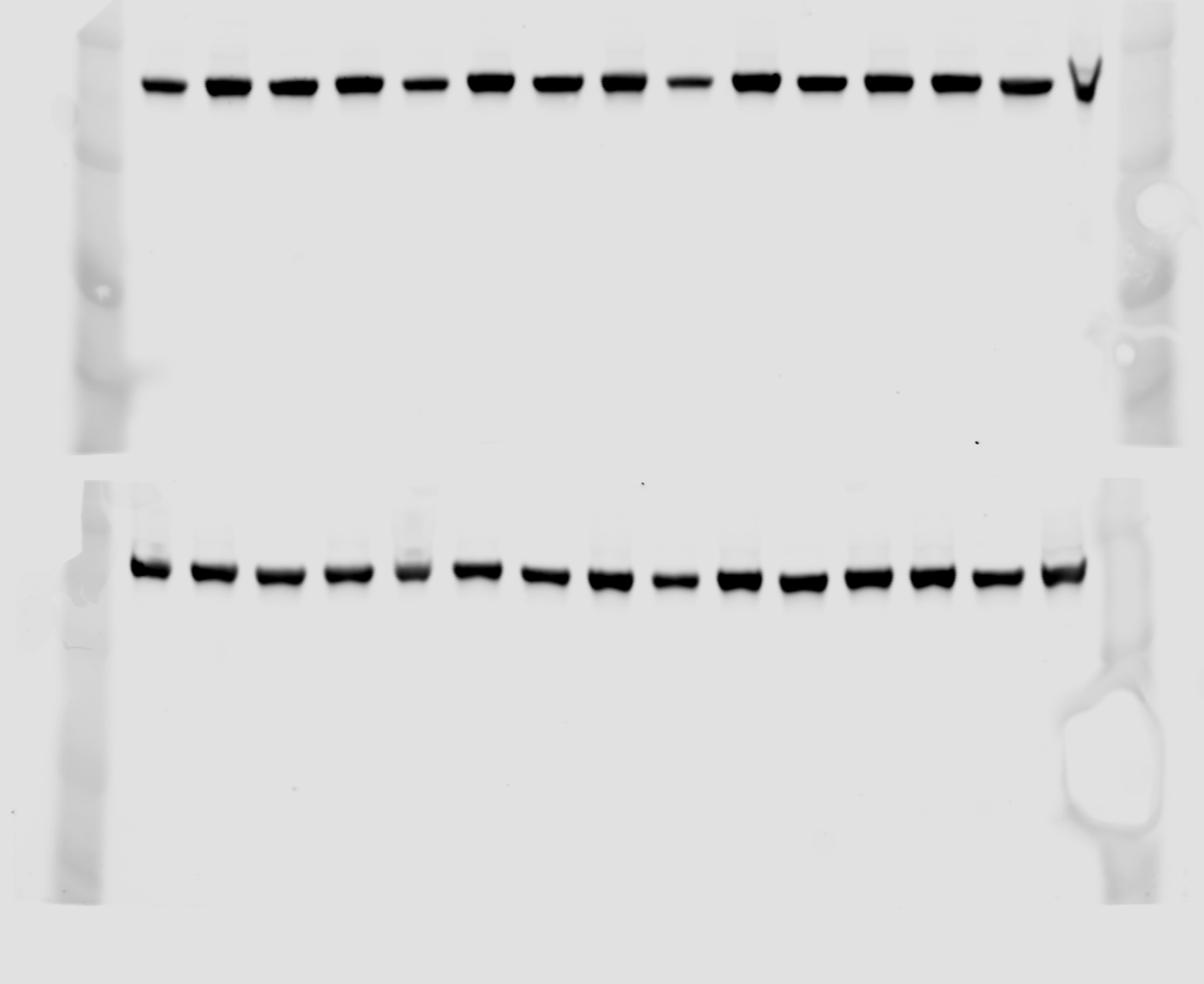

Supplement: Figure 3—figure supplement 1—source data 2. — The dashed boxes indicate the areas of blots presented in the figure. [file elife-81892-fig3-figsupp1-data2.zip › Figure 3-figure supplement 1-source data 2/136-1.tif]

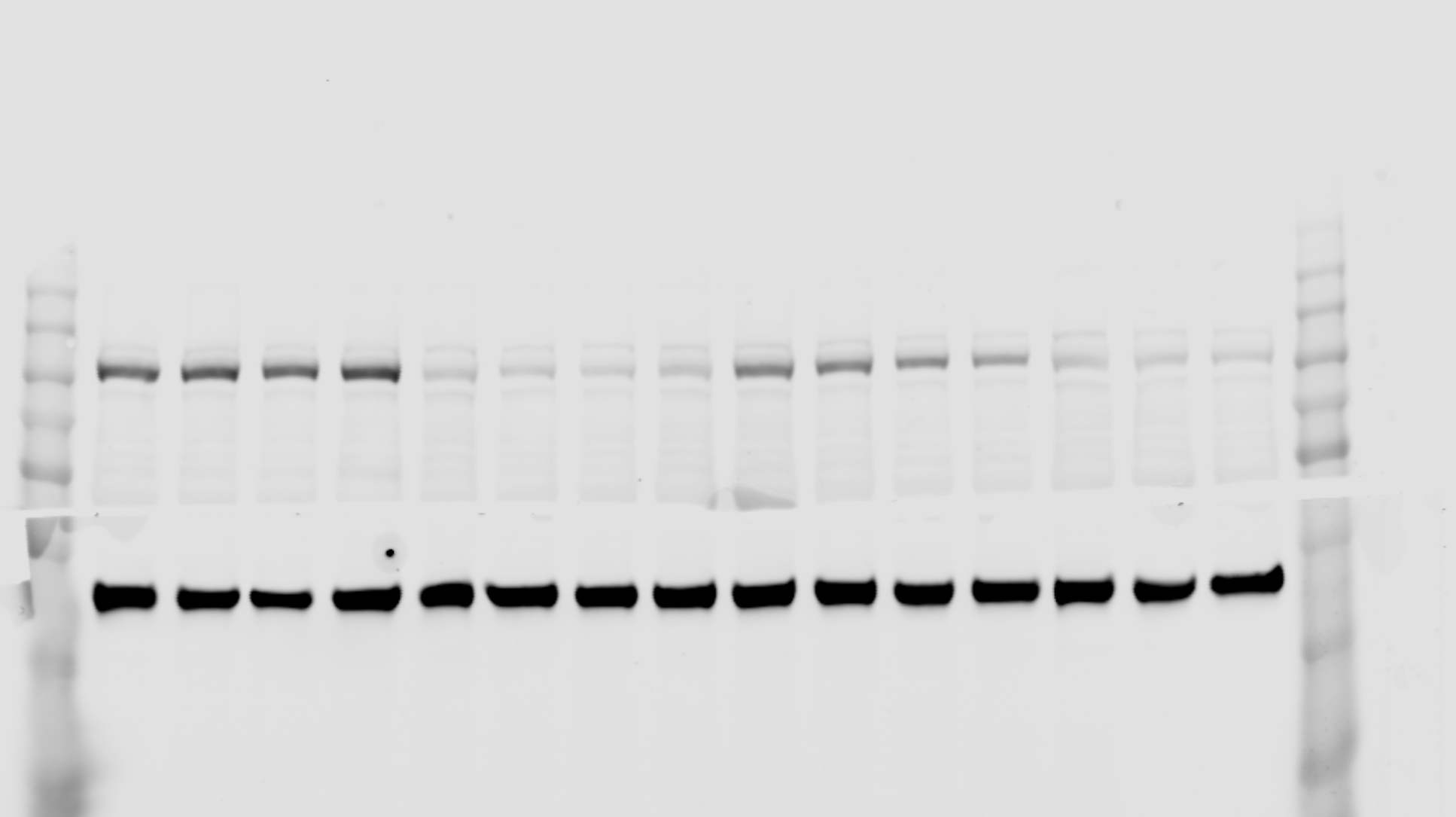

Supplement: Figure 3—figure supplement 1—source data 2. — The dashed boxes indicate the areas of blots presented in the figure. [file elife-81892-fig3-figsupp1-data2.zip › Figure 3-figure supplement 1-source data 2/140-2.tif]

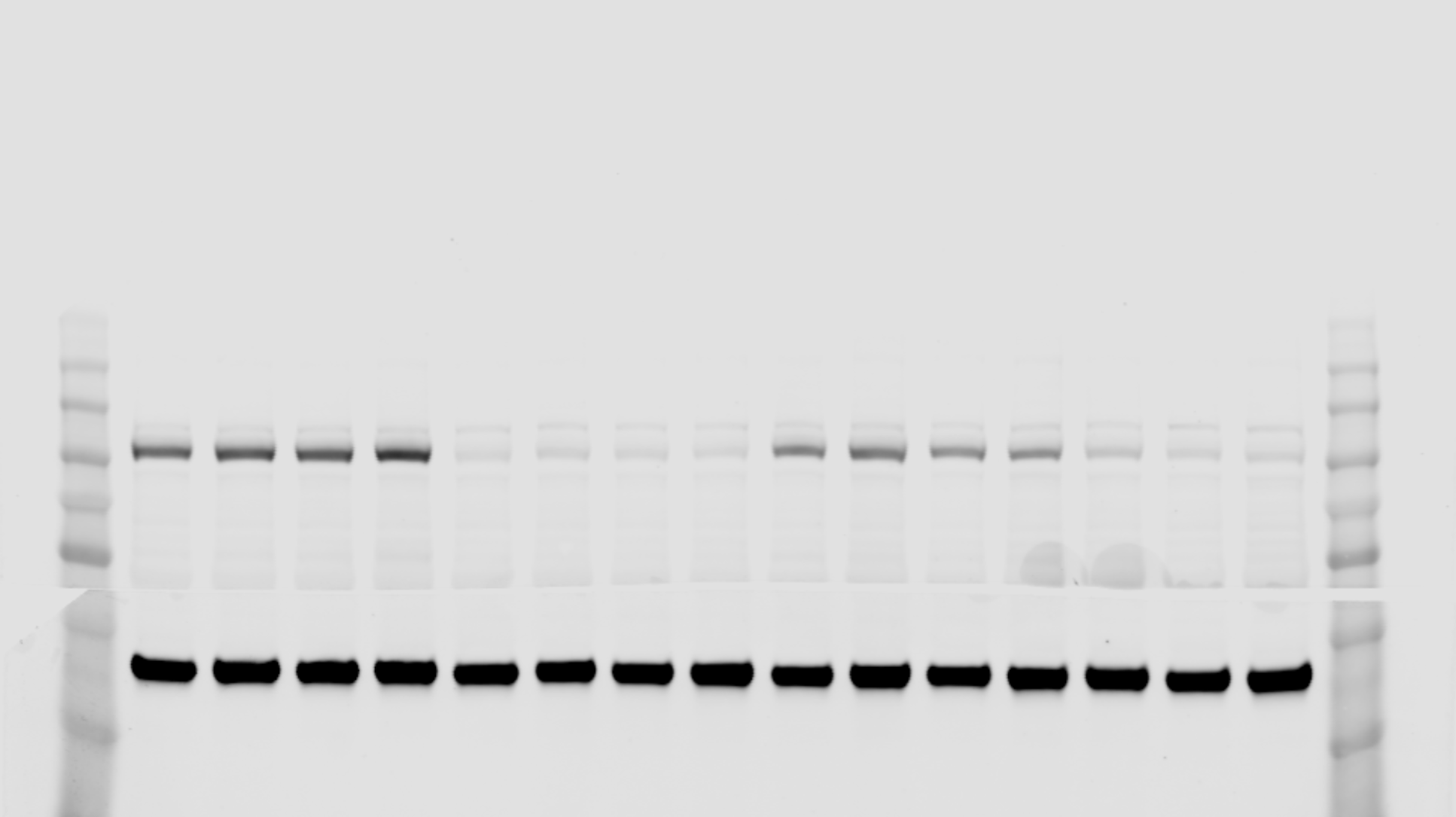

Supplement: Figure 3—figure supplement 1—source data 2. — The dashed boxes indicate the areas of blots presented in the figure. [file elife-81892-fig3-figsupp1-data2.zip › Figure 3-figure supplement 1-source data 2/142-2.tif]

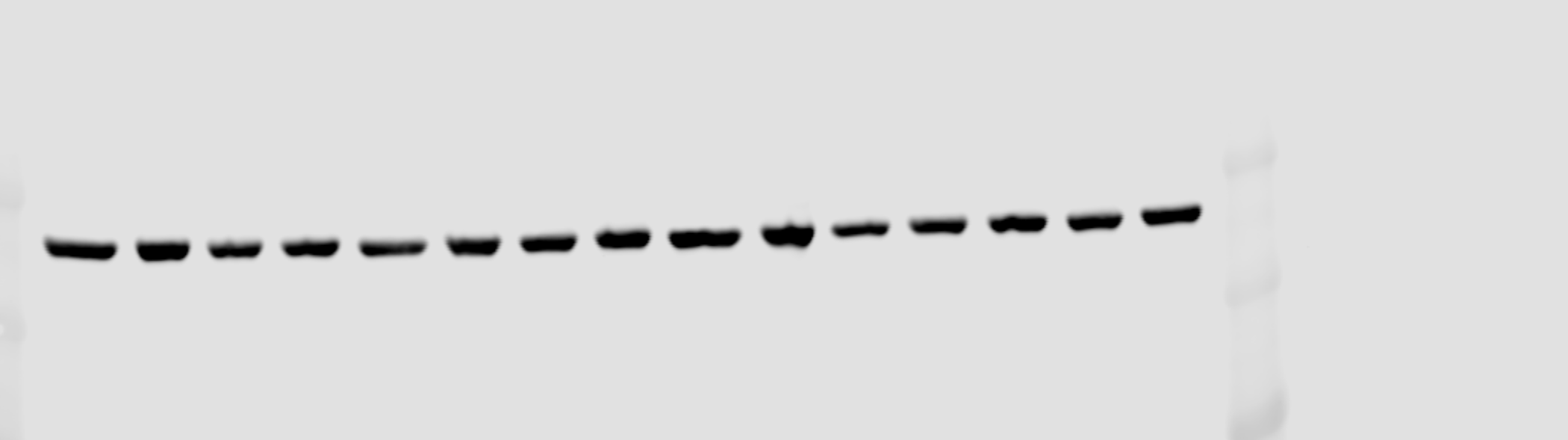

Supplement: Figure 3—figure supplement 1—source data 2. — The dashed boxes indicate the areas of blots presented in the figure. [file elife-81892-fig3-figsupp1-data2.zip › Figure 3-figure supplement 1-source data 2/192-1.tif]

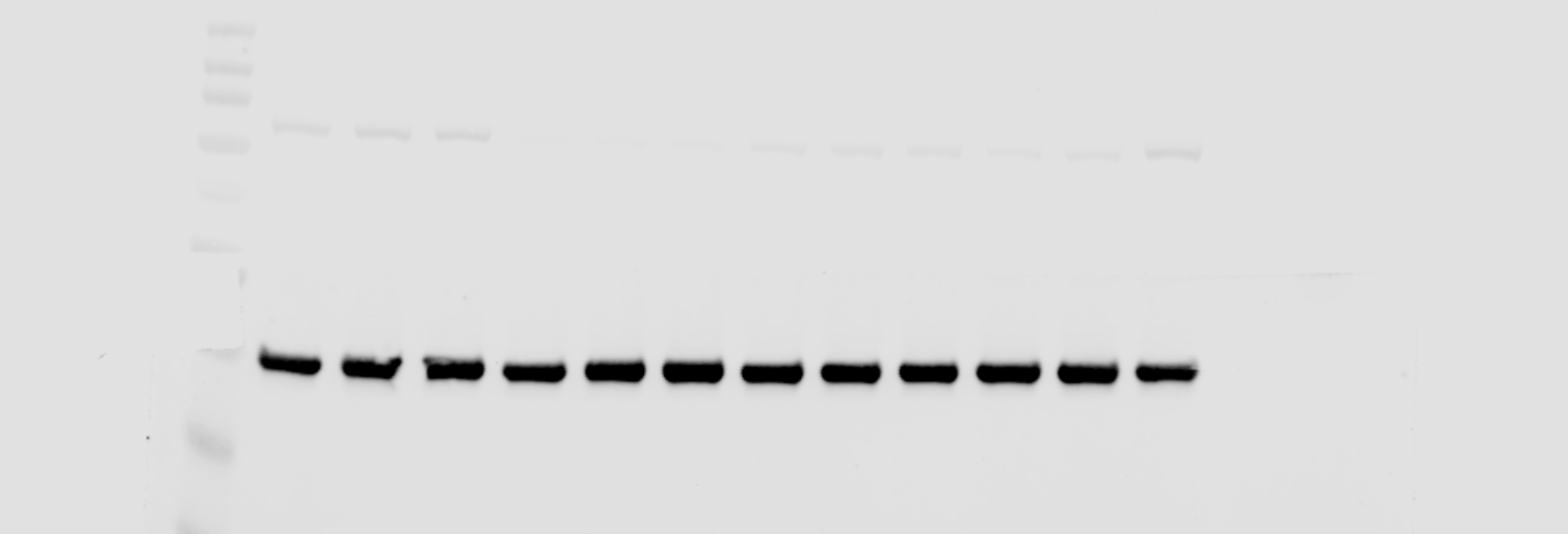

Supplement: Figure 3—figure supplement 1—source data 3. — The dashed boxes indicate the areas of blots presented in the figure. [file elife-81892-fig3-figsupp1-data3.zip › Figure 3-figure supplement 1-source data 3/240-1.tif]

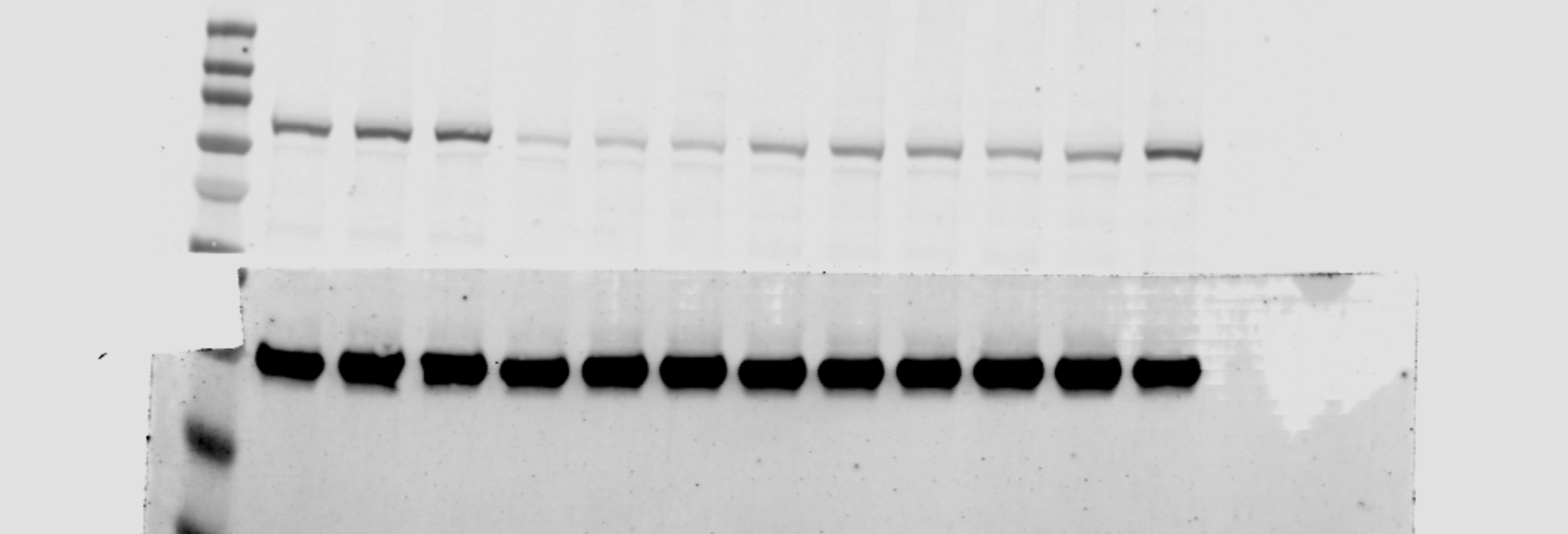

Supplement: Figure 3—figure supplement 1—source data 3. — The dashed boxes indicate the areas of blots presented in the figure. [file elife-81892-fig3-figsupp1-data3.zip › Figure 3-figure supplement 1-source data 3/240-2.tif]

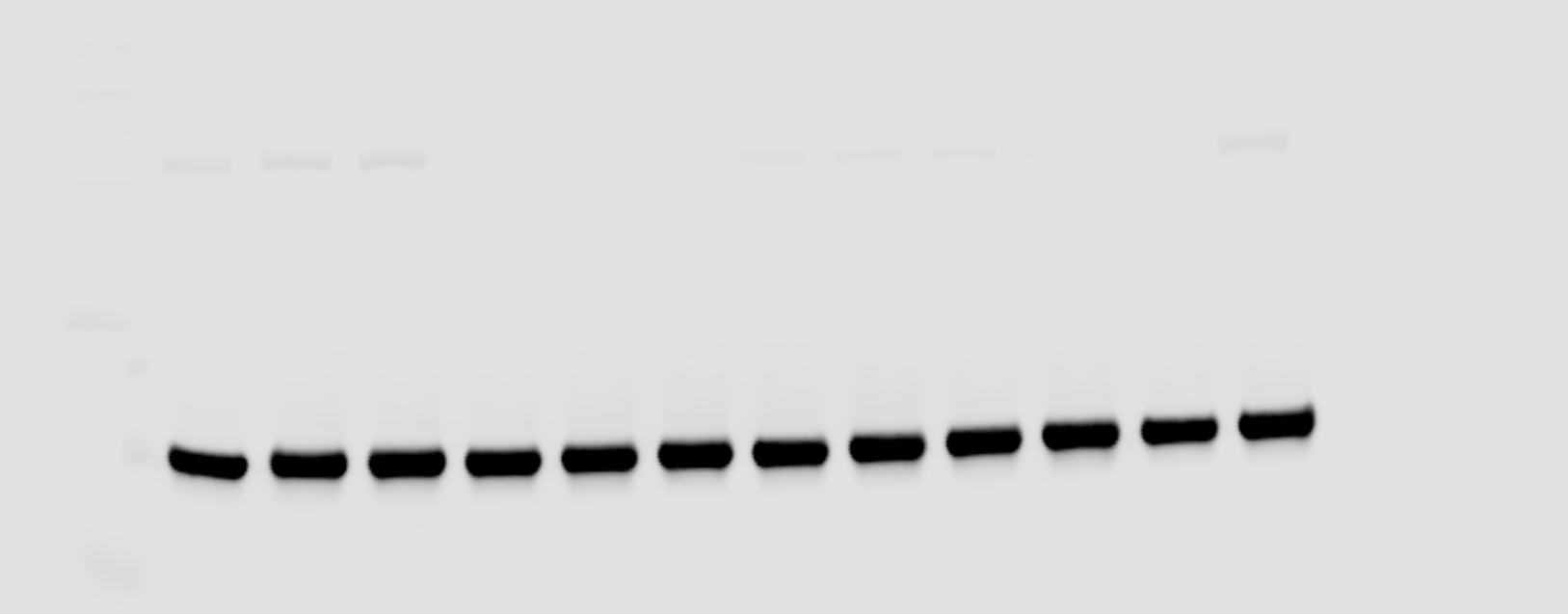

Supplement: Figure 3—figure supplement 1—source data 3. — The dashed boxes indicate the areas of blots presented in the figure. [file elife-81892-fig3-figsupp1-data3.zip › Figure 3-figure supplement 1-source data 3/244-1.tif]

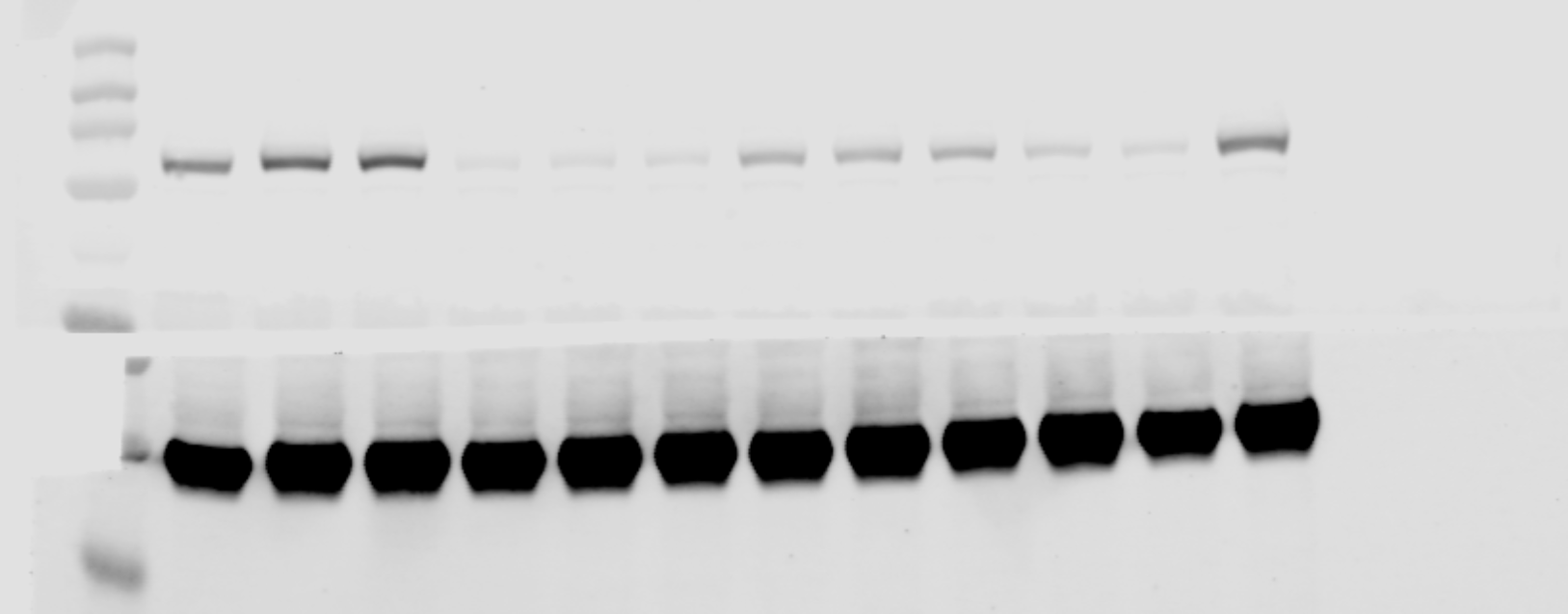

Supplement: Figure 3—figure supplement 1—source data 3. — The dashed boxes indicate the areas of blots presented in the figure. [file elife-81892-fig3-figsupp1-data3.zip › Figure 3-figure supplement 1-source data 3/244-2.tif]

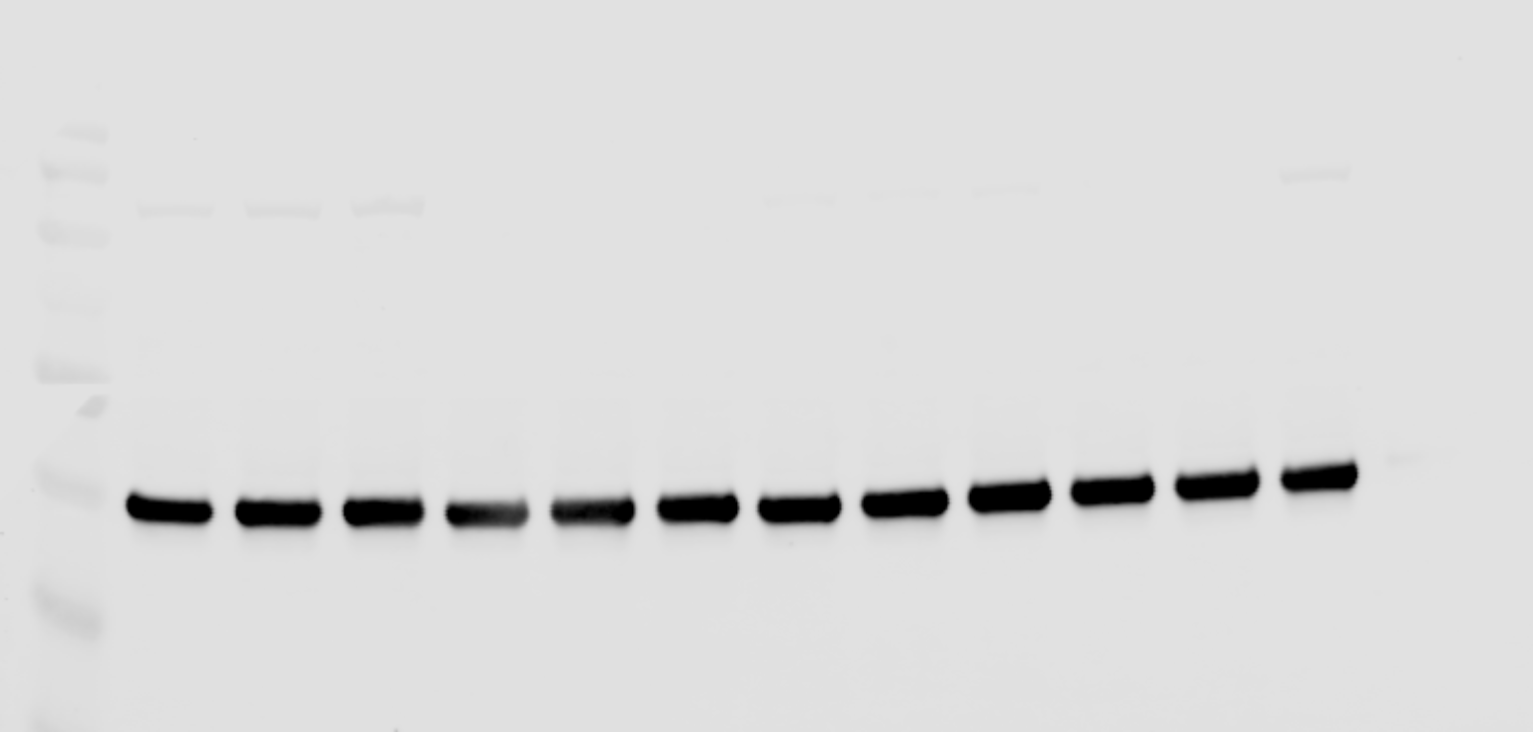

Supplement: Figure 3—figure supplement 1—source data 3. — The dashed boxes indicate the areas of blots presented in the figure. [file elife-81892-fig3-figsupp1-data3.zip › Figure 3-figure supplement 1-source data 3/246-1.tif]

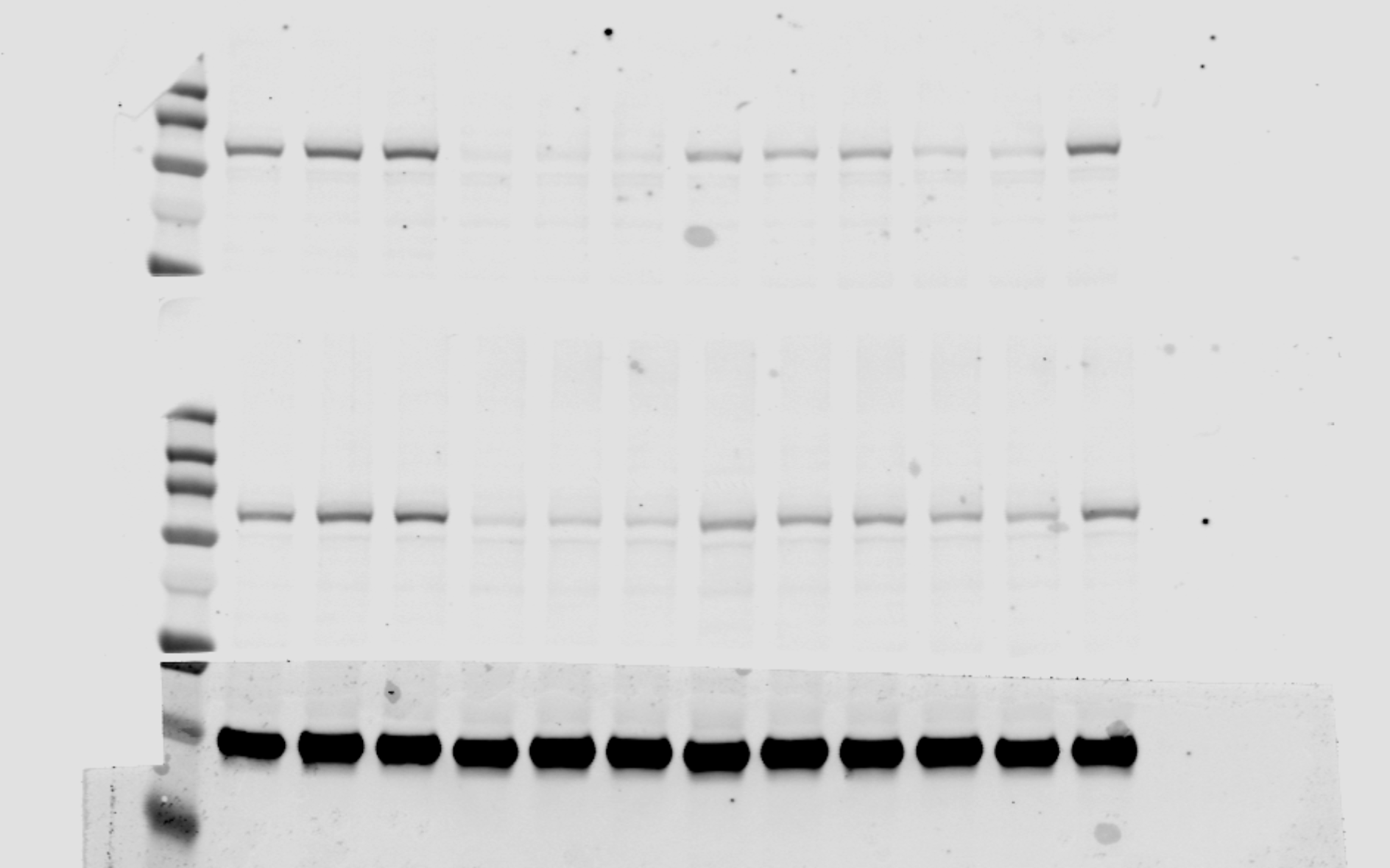

Supplement: Figure 3—figure supplement 1—source data 3. — The dashed boxes indicate the areas of blots presented in the figure. [file elife-81892-fig3-figsupp1-data3.zip › Figure 3-figure supplement 1-source data 3/248-1.tif]

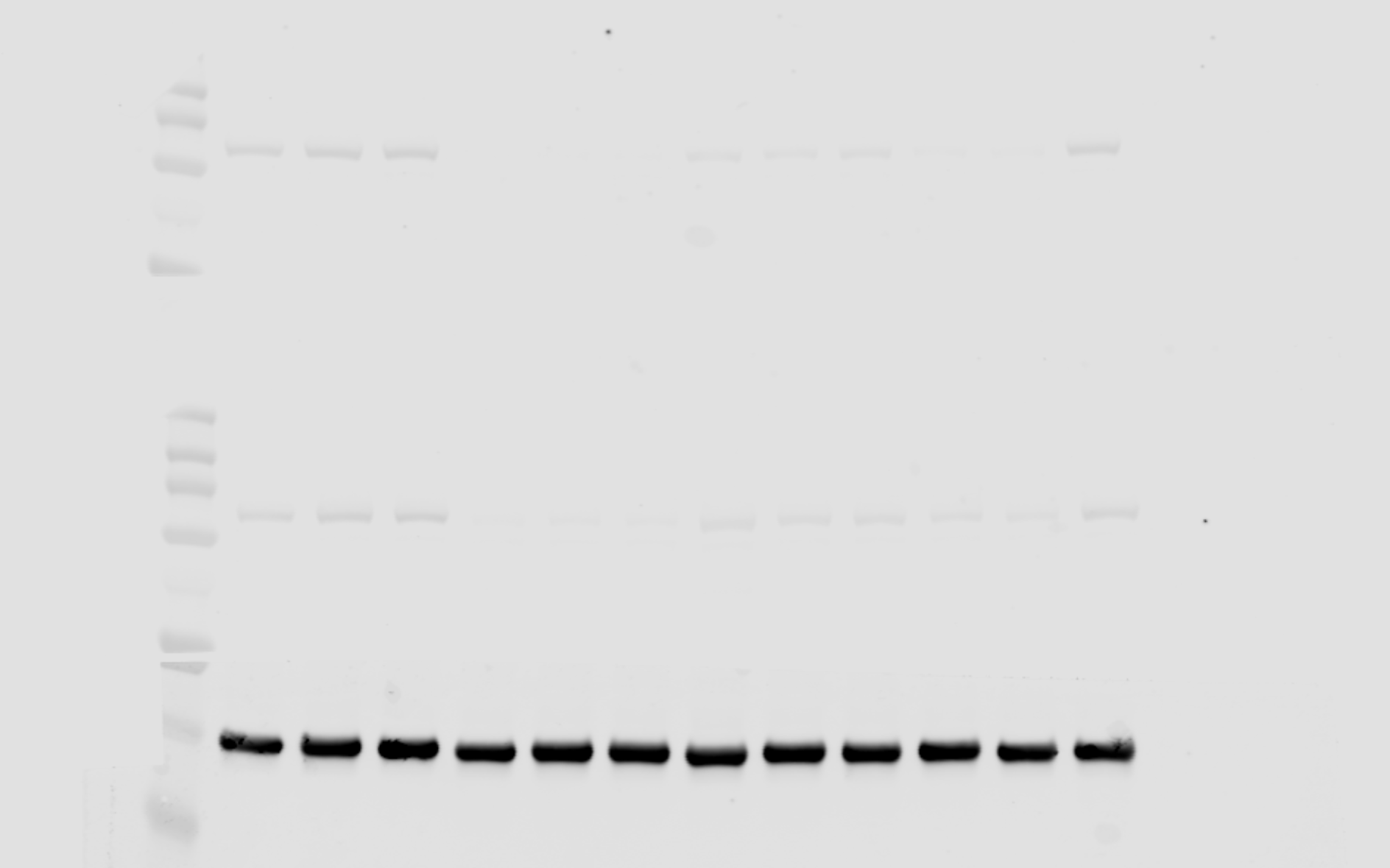

Supplement: Figure 3—figure supplement 1—source data 3. — The dashed boxes indicate the areas of blots presented in the figure. [file elife-81892-fig3-figsupp1-data3.zip › Figure 3-figure supplement 1-source data 3/248-2.tif]

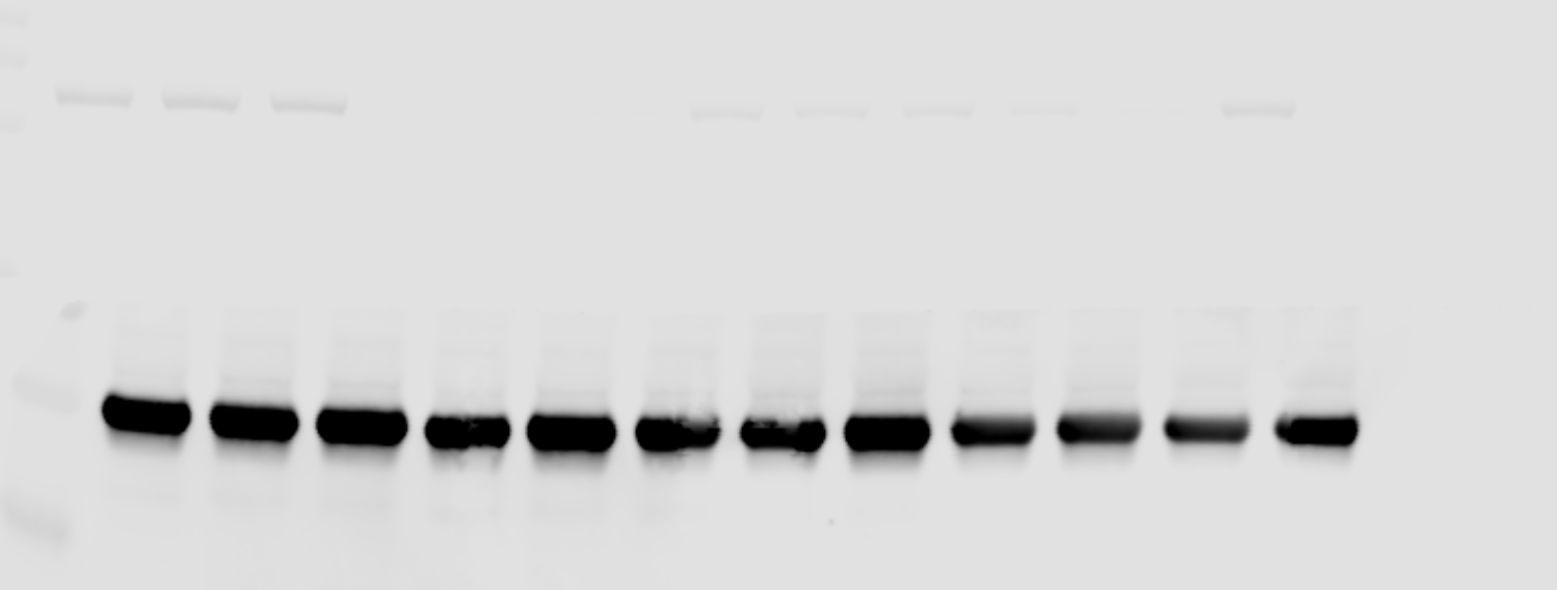

Supplement: Figure 3—figure supplement 1—source data 3. — The dashed boxes indicate the areas of blots presented in the figure. [file elife-81892-fig3-figsupp1-data3.zip › Figure 3-figure supplement 1-source data 3/254-1.tif]

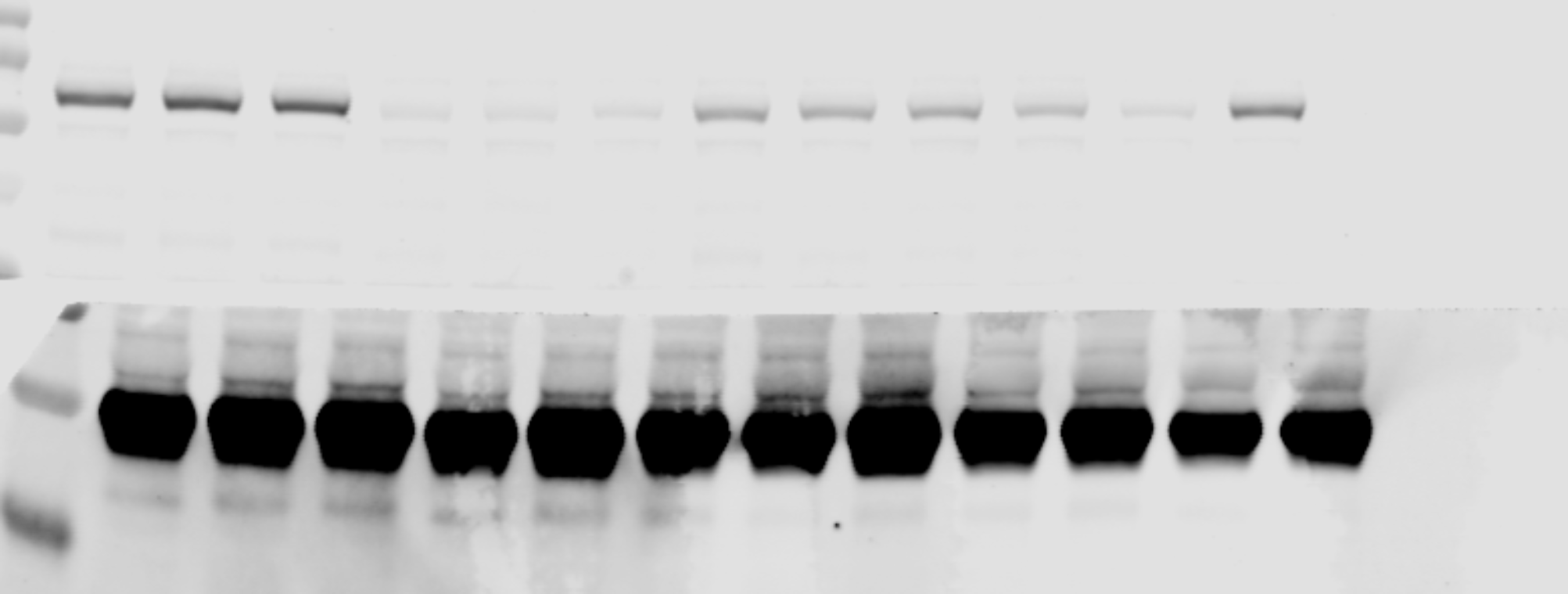

Supplement: Figure 3—figure supplement 1—source data 3. — The dashed boxes indicate the areas of blots presented in the figure. [file elife-81892-fig3-figsupp1-data3.zip › Figure 3-figure supplement 1-source data 3/254-2.tif]

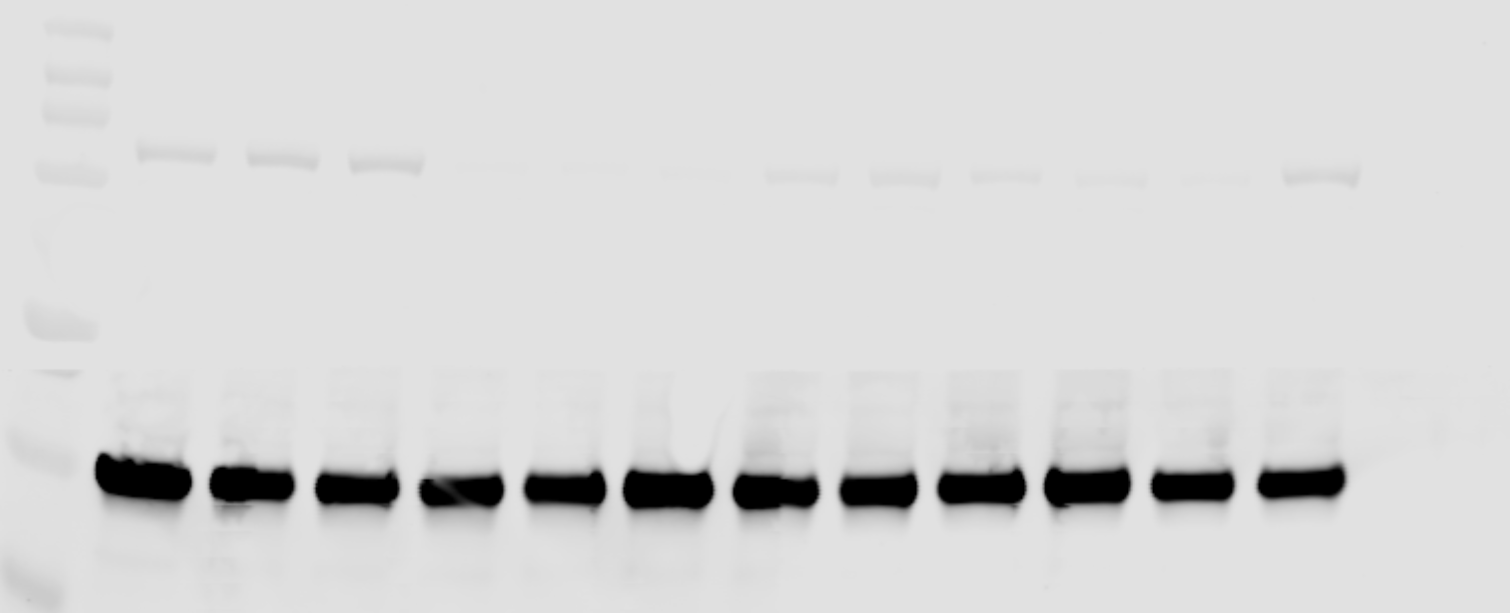

Supplement: Figure 3—figure supplement 1—source data 3. — The dashed boxes indicate the areas of blots presented in the figure. [file elife-81892-fig3-figsupp1-data3.zip › Figure 3-figure supplement 1-source data 3/256-1.tif]

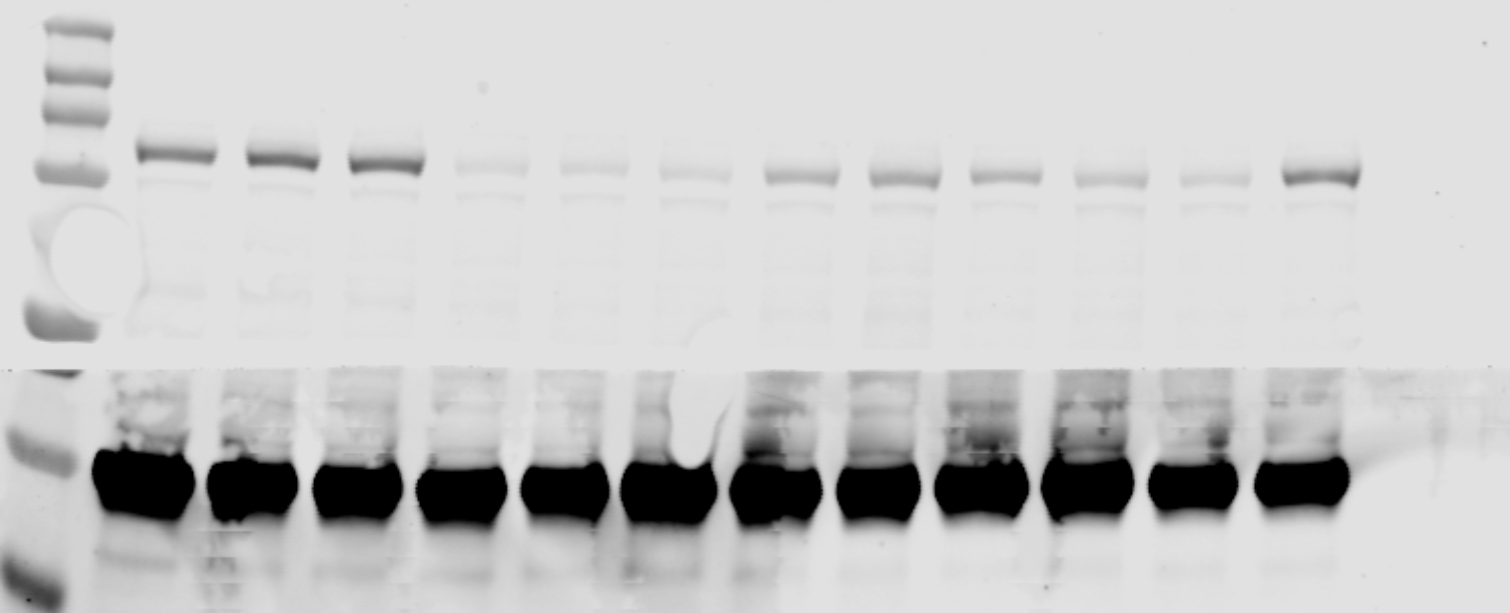

Supplement: Figure 3—figure supplement 1—source data 3. — The dashed boxes indicate the areas of blots presented in the figure. [file elife-81892-fig3-figsupp1-data3.zip › Figure 3-figure supplement 1-source data 3/256-2.tif]

Figure 3-supplement 1B. Olfactory bulb

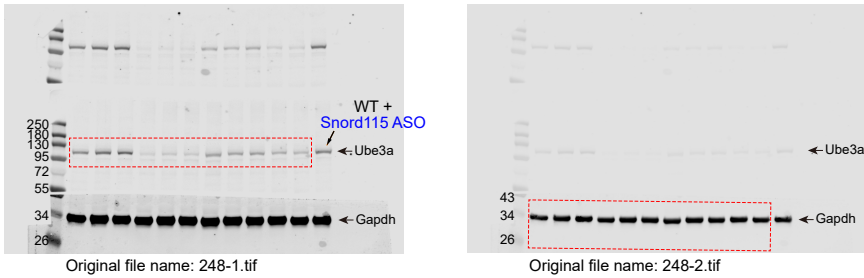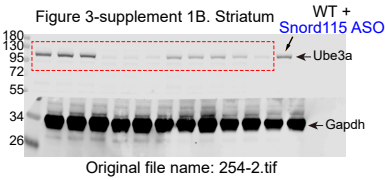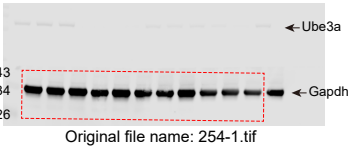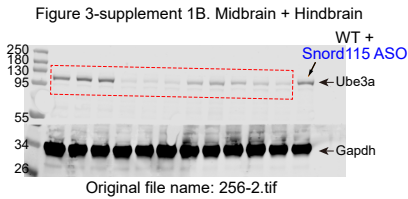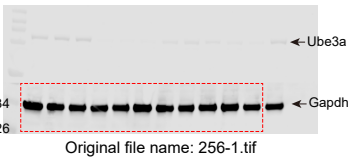

Supplement: Figure 3—figure supplement 1—source data 3. — The dashed boxes indicate the areas of blots presented in the figure. [file elife-81892-fig3-figsupp1-data3.zip › Figure 3-figure supplement 1-source data 3/Figure 3-figure supplement 1-source data 3b WB adult p3wks 221223.pdf]

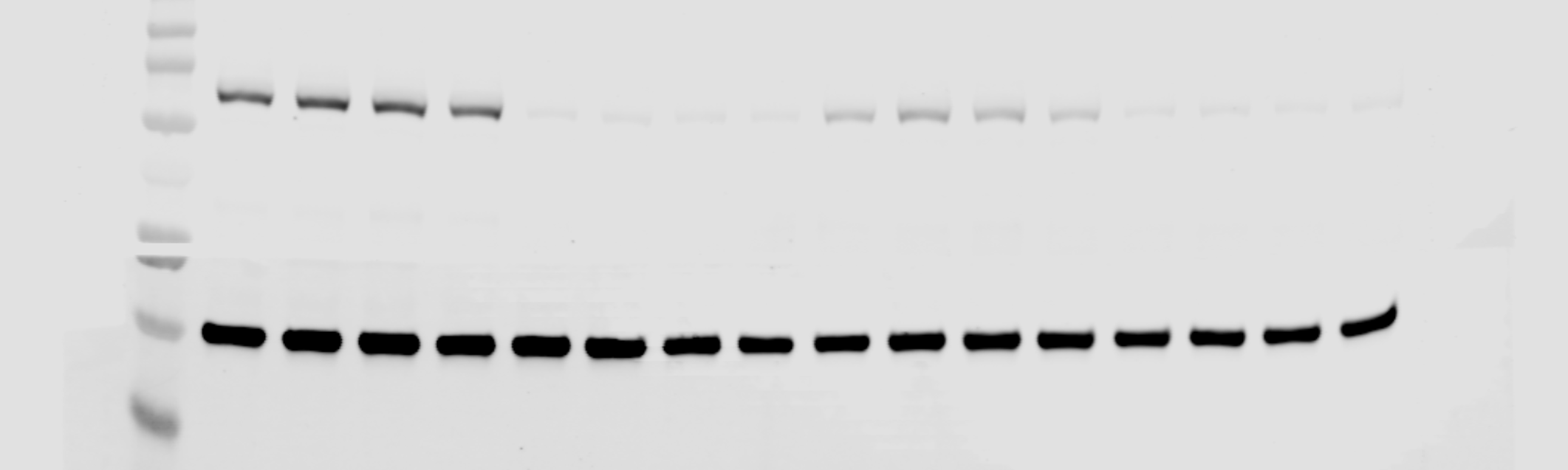

Supplement: Figure 3—figure supplement 1—source data 4. — The dashed boxes indicate the areas of blots presented in the figure. [file elife-81892-fig3-figsupp1-data4.zip › Figure 3-figure supplement 1-source data 4/217-1.tif]

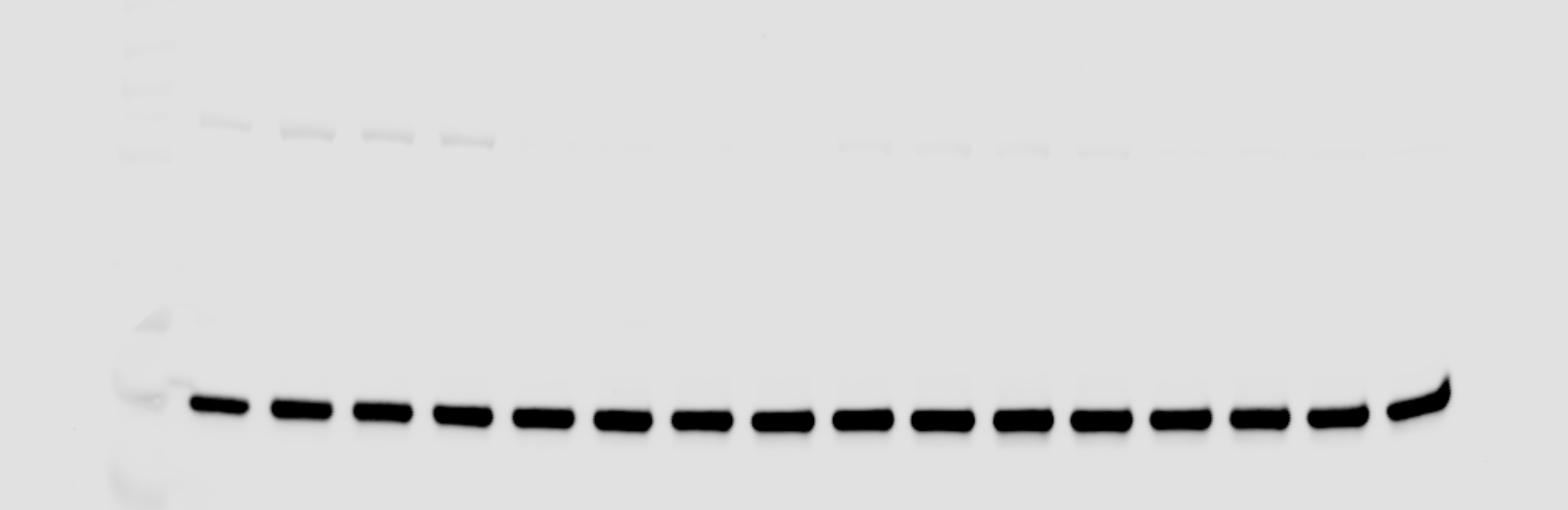

Supplement: Figure 3—figure supplement 1—source data 4. — The dashed boxes indicate the areas of blots presented in the figure. [file elife-81892-fig3-figsupp1-data4.zip › Figure 3-figure supplement 1-source data 4/222-1.tif]

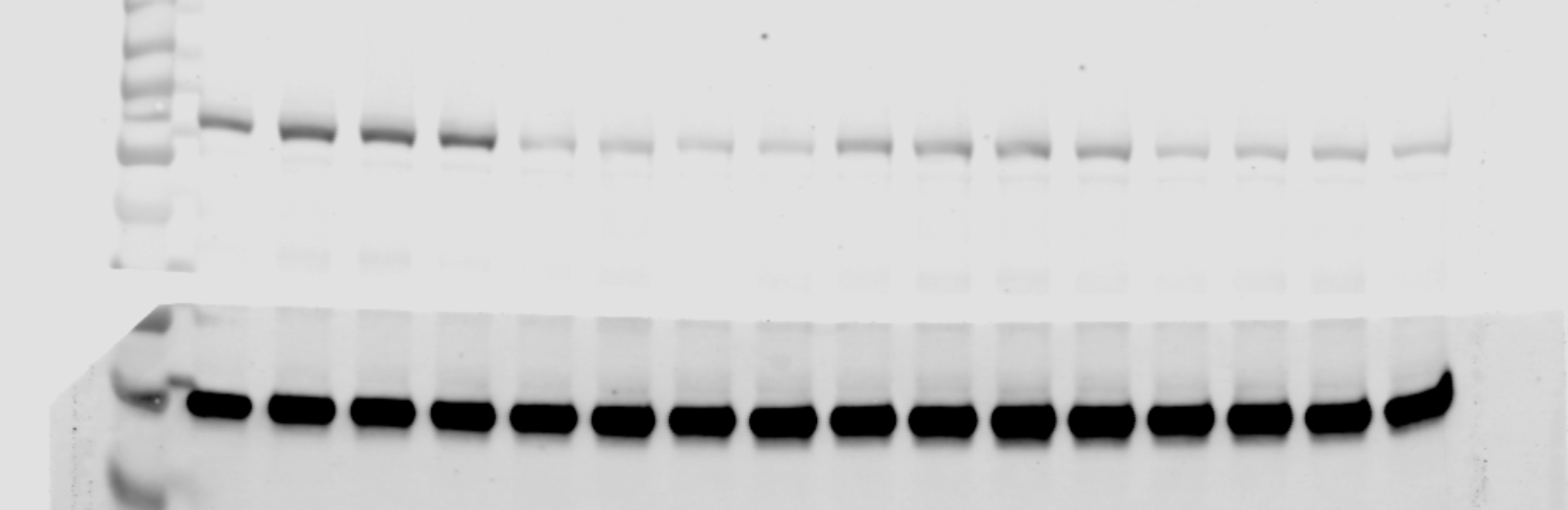

Supplement: Figure 3—figure supplement 1—source data 4. — The dashed boxes indicate the areas of blots presented in the figure. [file elife-81892-fig3-figsupp1-data4.zip › Figure 3-figure supplement 1-source data 4/222-2.tif]

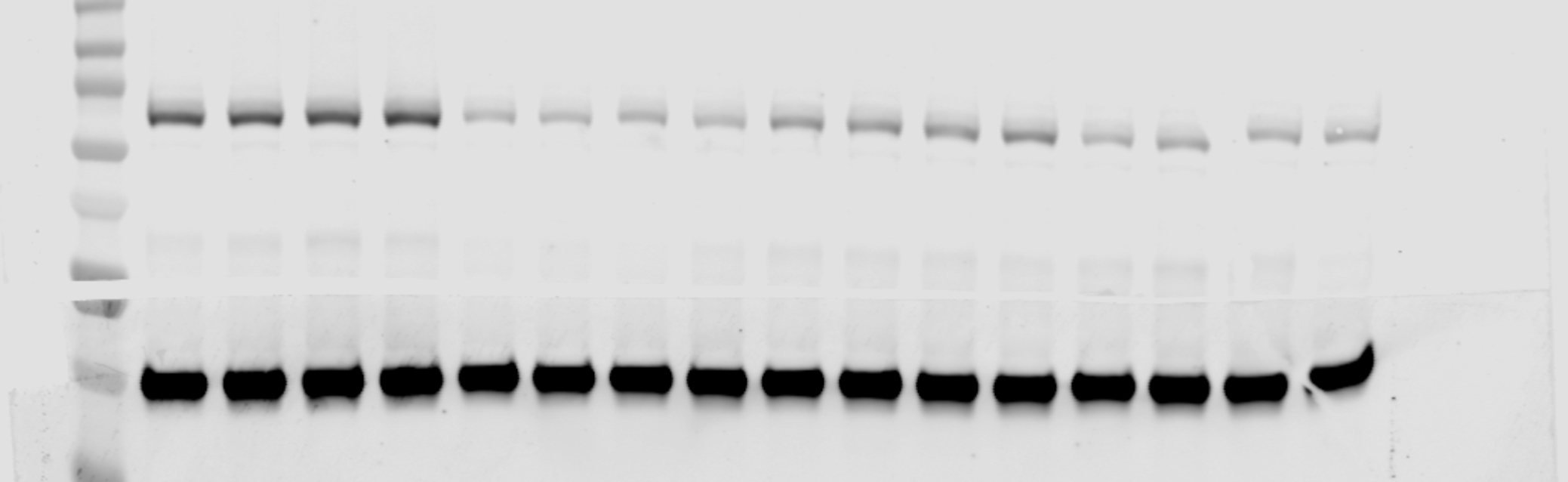

Supplement: Figure 3—figure supplement 1—source data 4. — The dashed boxes indicate the areas of blots presented in the figure. [file elife-81892-fig3-figsupp1-data4.zip › Figure 3-figure supplement 1-source data 4/226-1.tif]

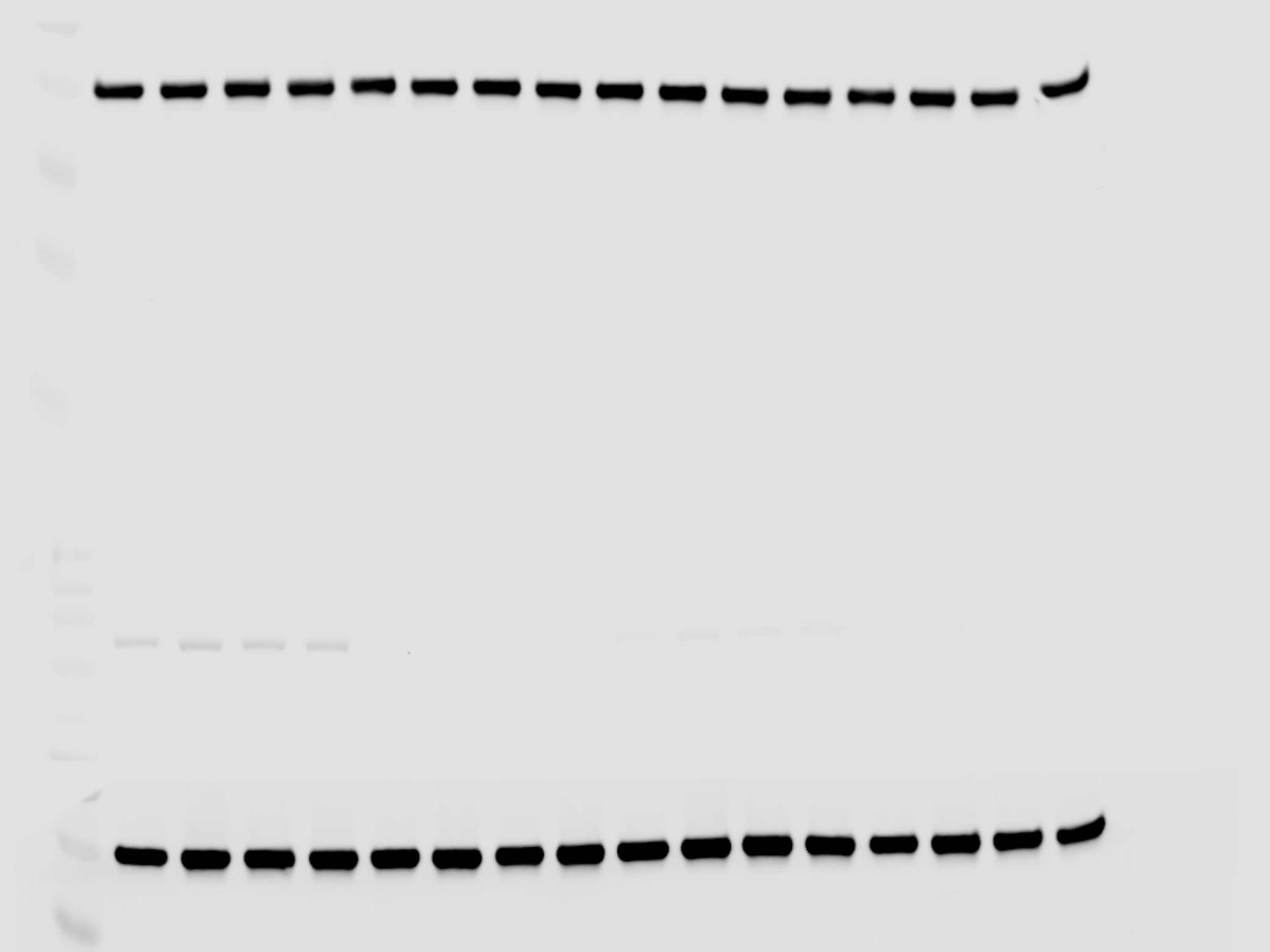

Supplement: Figure 3—figure supplement 1—source data 4. — The dashed boxes indicate the areas of blots presented in the figure. [file elife-81892-fig3-figsupp1-data4.zip › Figure 3-figure supplement 1-source data 4/228-1.tif]

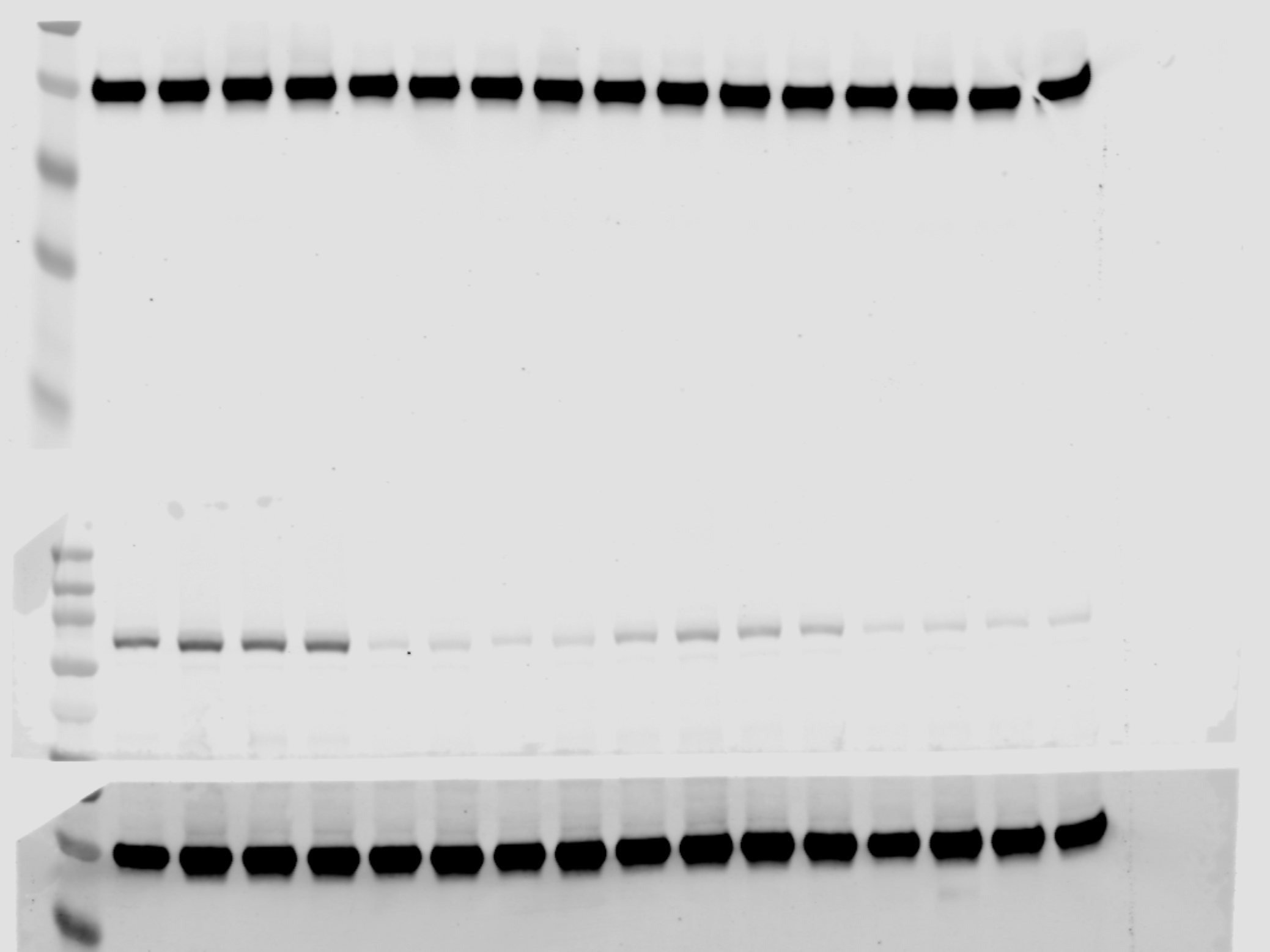

Supplement: Figure 3—figure supplement 1—source data 4. — The dashed boxes indicate the areas of blots presented in the figure. [file elife-81892-fig3-figsupp1-data4.zip › Figure 3-figure supplement 1-source data 4/228-2.tif]

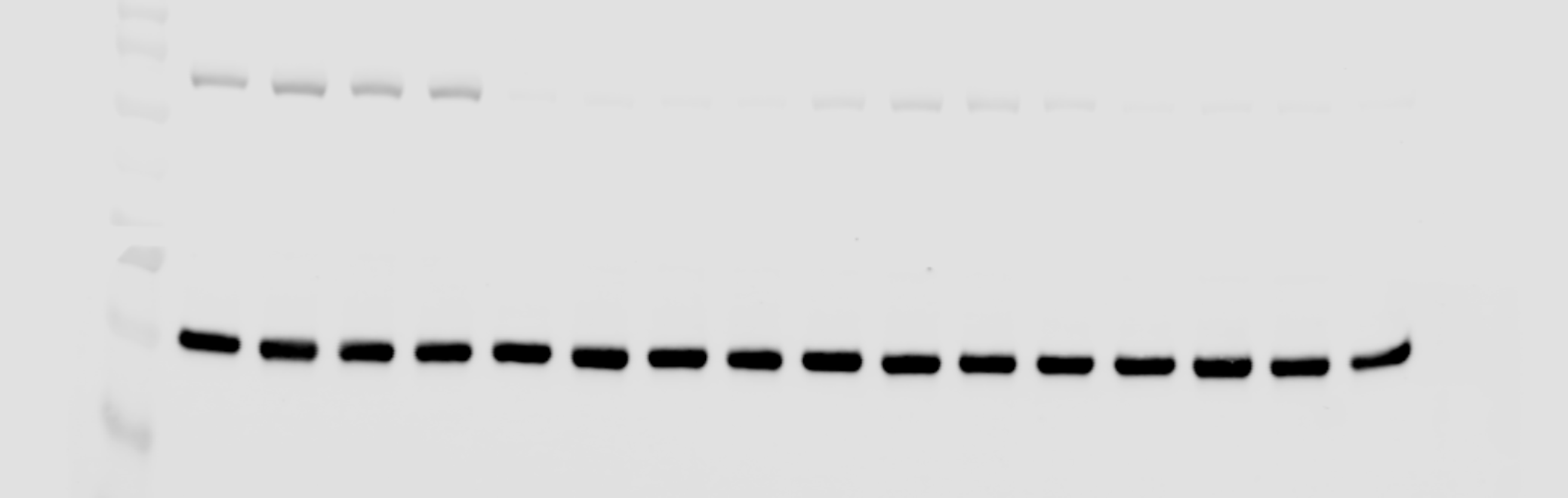

Supplement: Figure 3—figure supplement 1—source data 4. — The dashed boxes indicate the areas of blots presented in the figure. [file elife-81892-fig3-figsupp1-data4.zip › Figure 3-figure supplement 1-source data 4/230-1.tif]

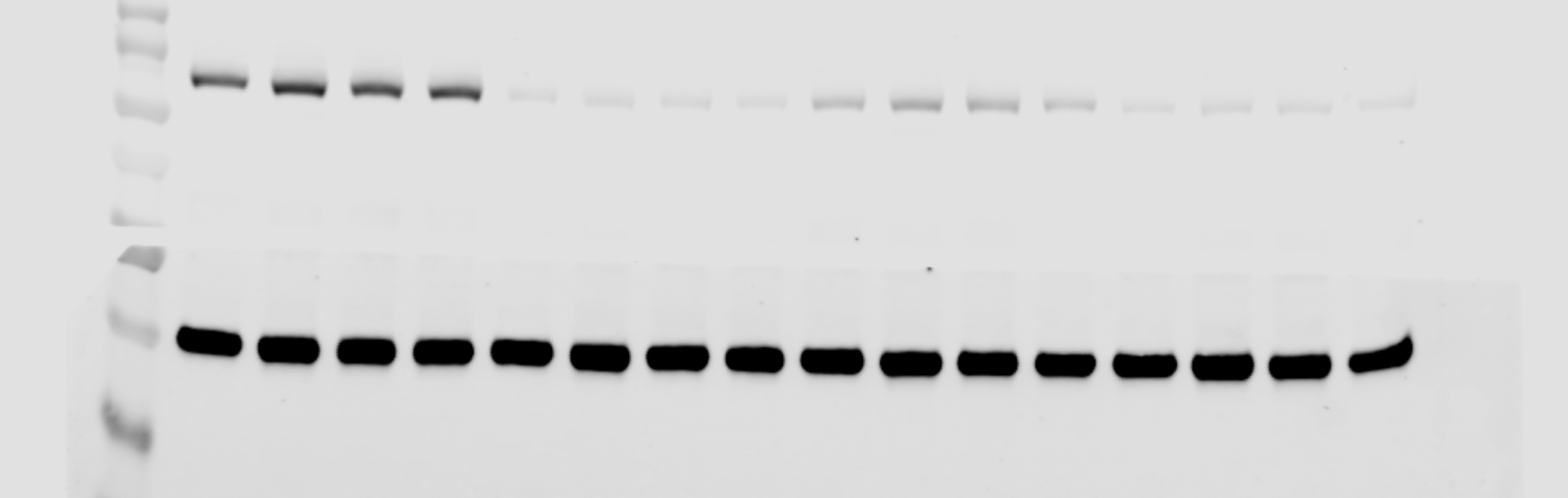

Supplement: Figure 3—figure supplement 1—source data 4. — The dashed boxes indicate the areas of blots presented in the figure. [file elife-81892-fig3-figsupp1-data4.zip › Figure 3-figure supplement 1-source data 4/230-2.tif]

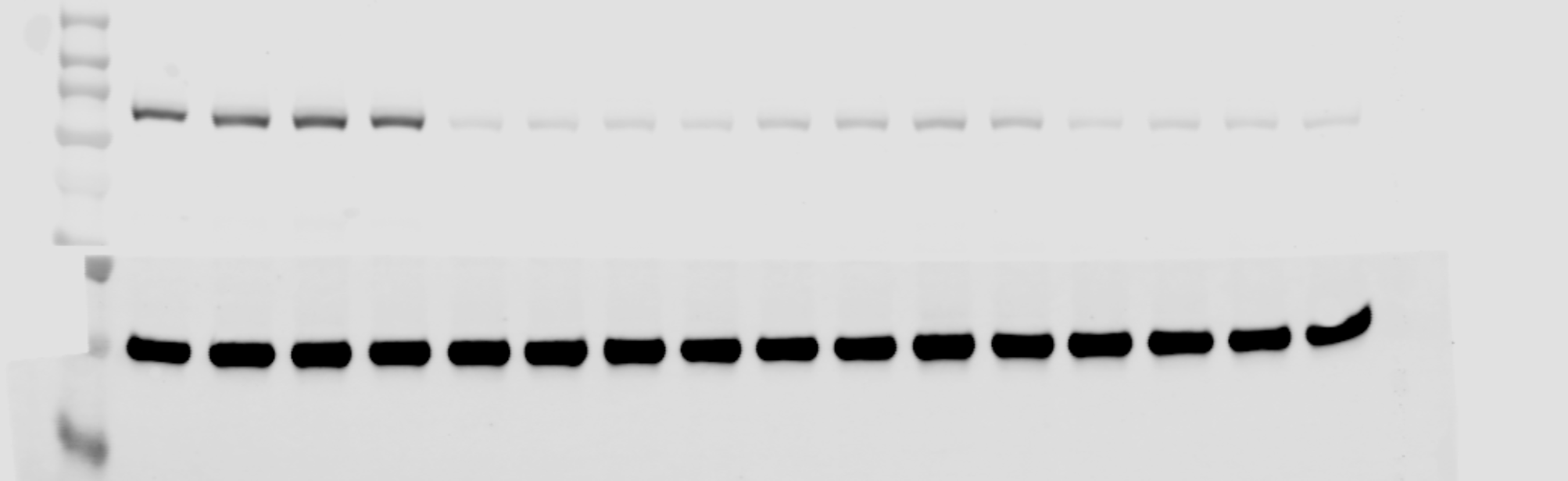

Supplement: Figure 3—figure supplement 1—source data 4. — The dashed boxes indicate the areas of blots presented in the figure. [file elife-81892-fig3-figsupp1-data4.zip › Figure 3-figure supplement 1-source data 4/232-1.tif]

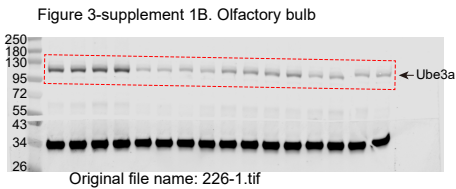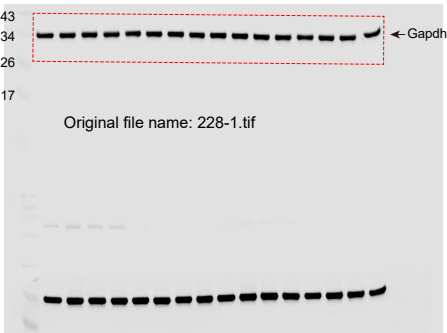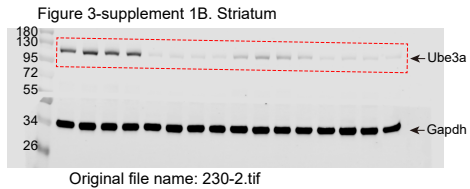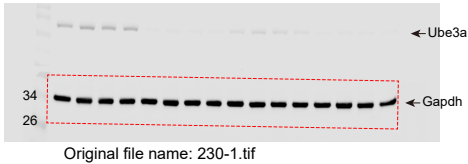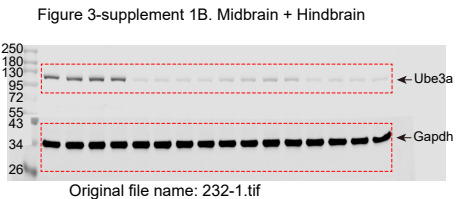

Supplement: Figure 3—figure supplement 1—source data 4. — The dashed boxes indicate the areas of blots presented in the figure. [file elife-81892-fig3-figsupp1-data4.zip › Figure 3-figure supplement 1-source data 4/Figure 3-figure supplement 1-source data 4b WB adult p10wks 221223.pdf]

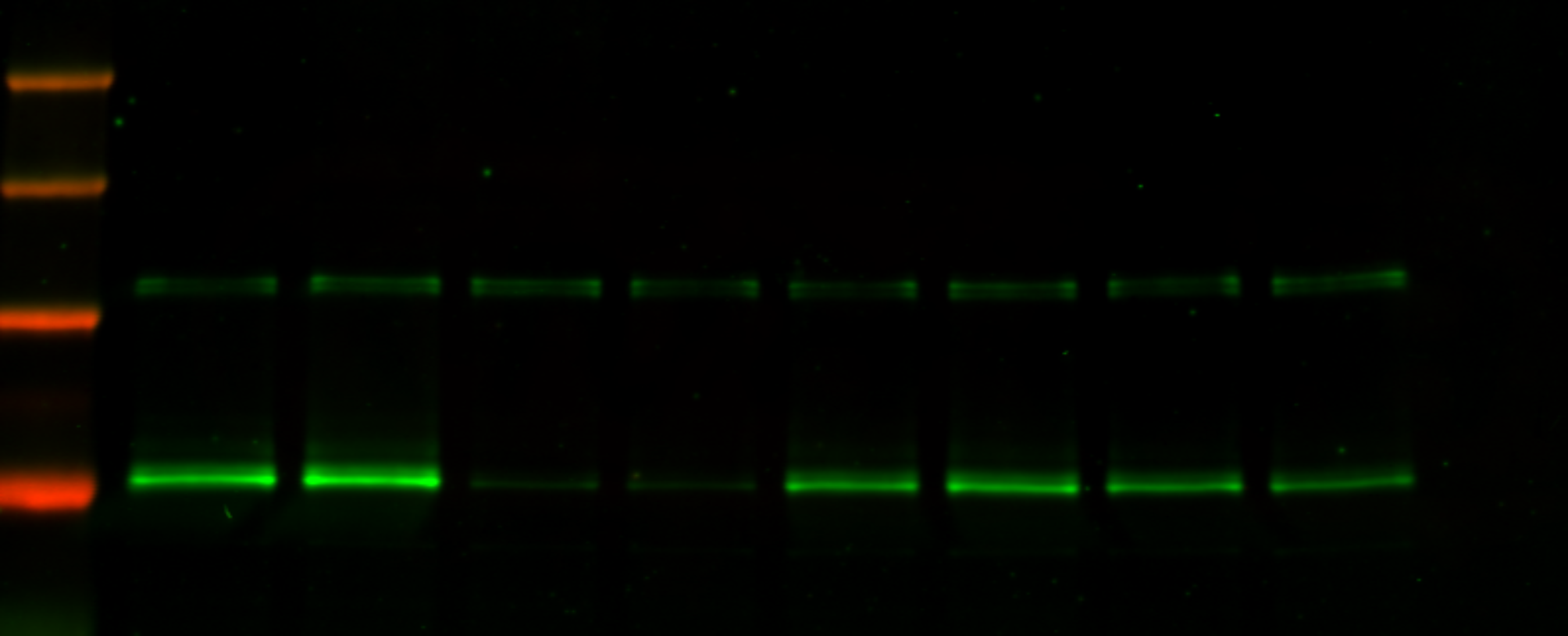

Supplement: Figure 3—figure supplement 3—source data 1. — The dashed boxes indicate the areas of blots presented in the figure. [file elife-81892-fig3-figsupp3-data1.zip › Figure 3-figure supplement 3-source data 1/7998-1.tif]

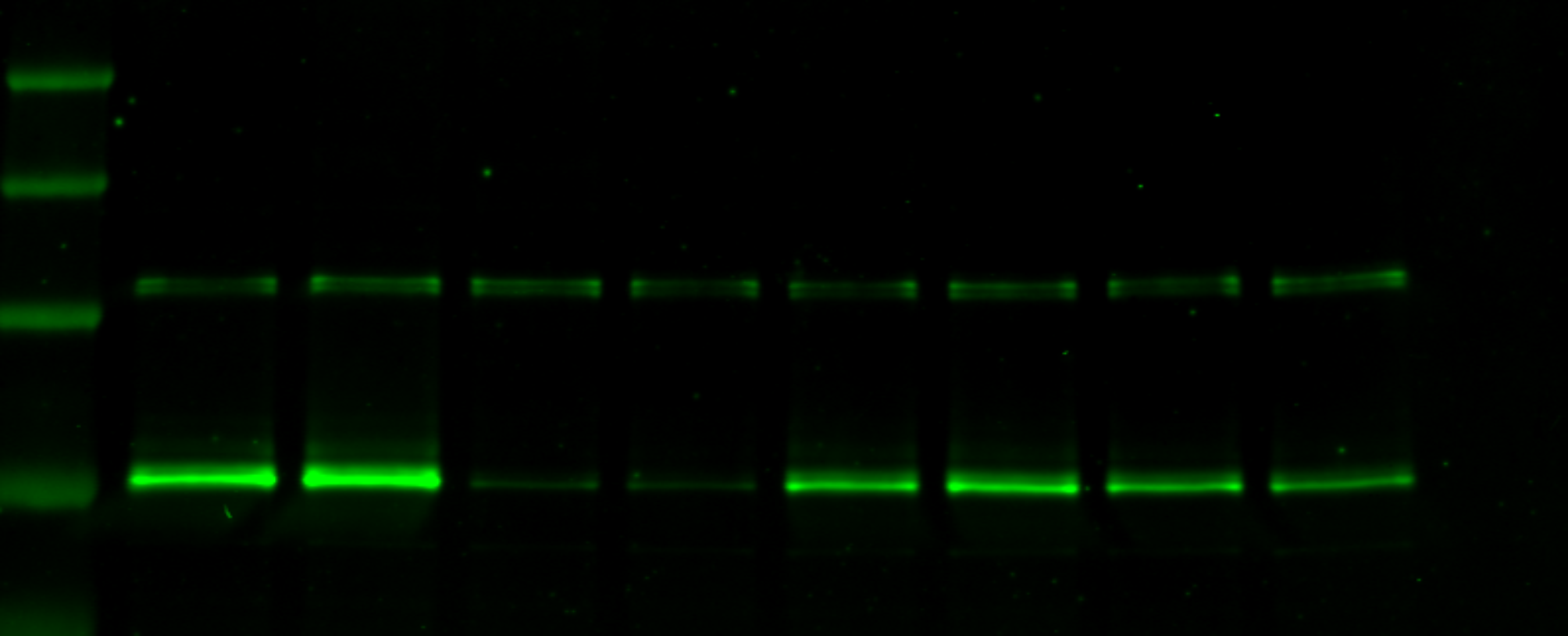

Supplement: Figure 3—figure supplement 3—source data 1. — The dashed boxes indicate the areas of blots presented in the figure. [file elife-81892-fig3-figsupp3-data1.zip › Figure 3-figure supplement 3-source data 1/7998-2.tif]

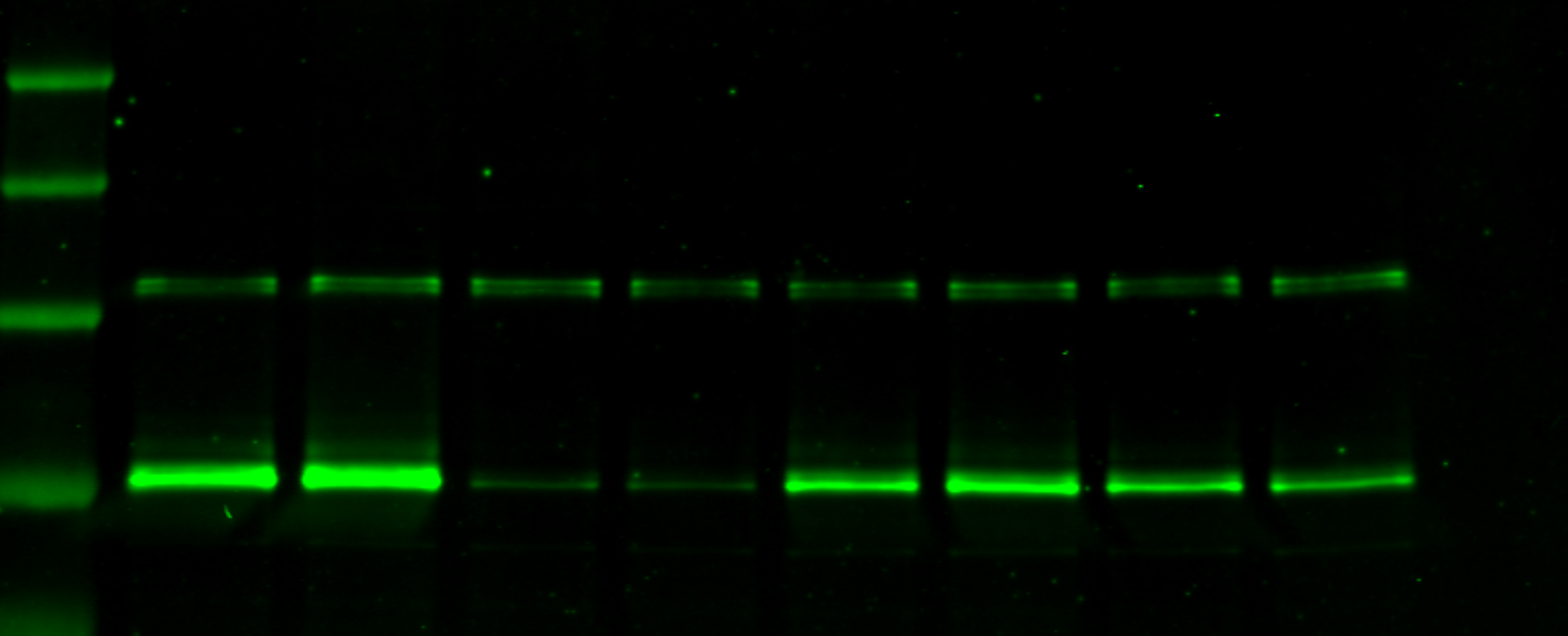

Supplement: Figure 3—figure supplement 3—source data 1. — The dashed boxes indicate the areas of blots presented in the figure. [file elife-81892-fig3-figsupp3-data1.zip › Figure 3-figure supplement 3-source data 1/7998-3.tif]

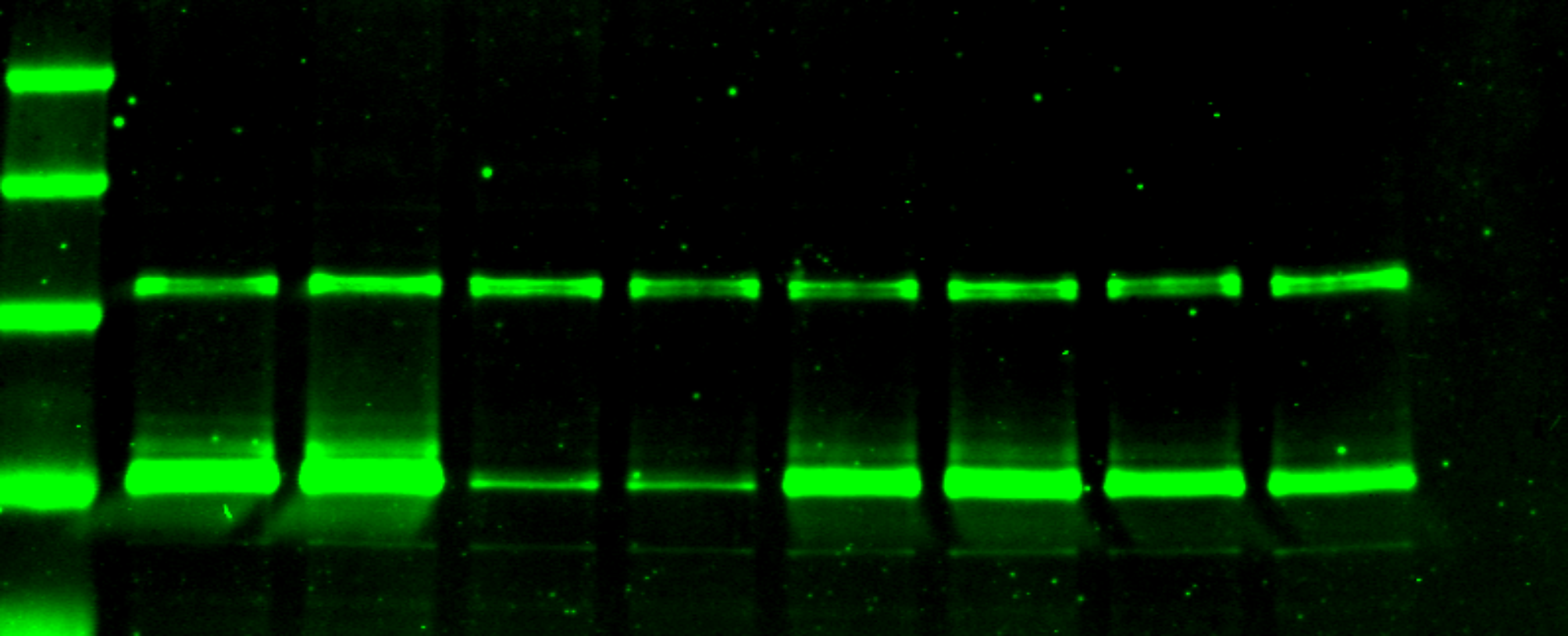

Supplement: Figure 3—figure supplement 3—source data 1. — The dashed boxes indicate the areas of blots presented in the figure. [file elife-81892-fig3-figsupp3-data1.zip › Figure 3-figure supplement 3-source data 1/7998-4.tif]

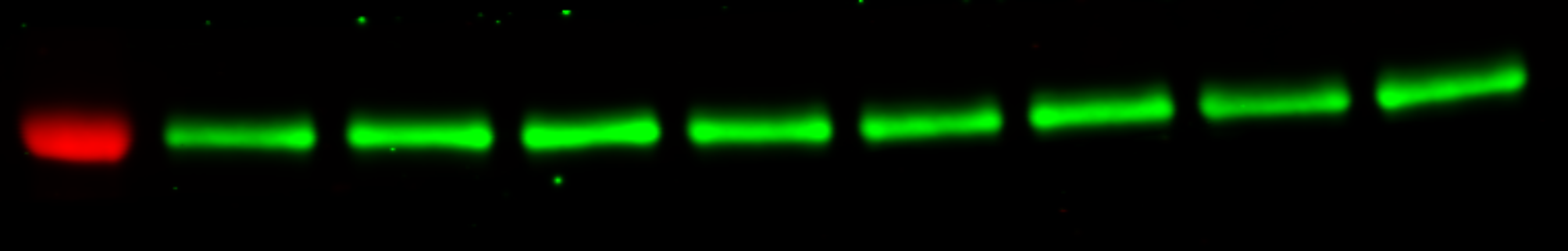

Supplement: Figure 3—figure supplement 3—source data 1. — The dashed boxes indicate the areas of blots presented in the figure. [file elife-81892-fig3-figsupp3-data1.zip › Figure 3-figure supplement 3-source data 1/8000-1.tif]

3 weeks post ASO injection to juvenile mice

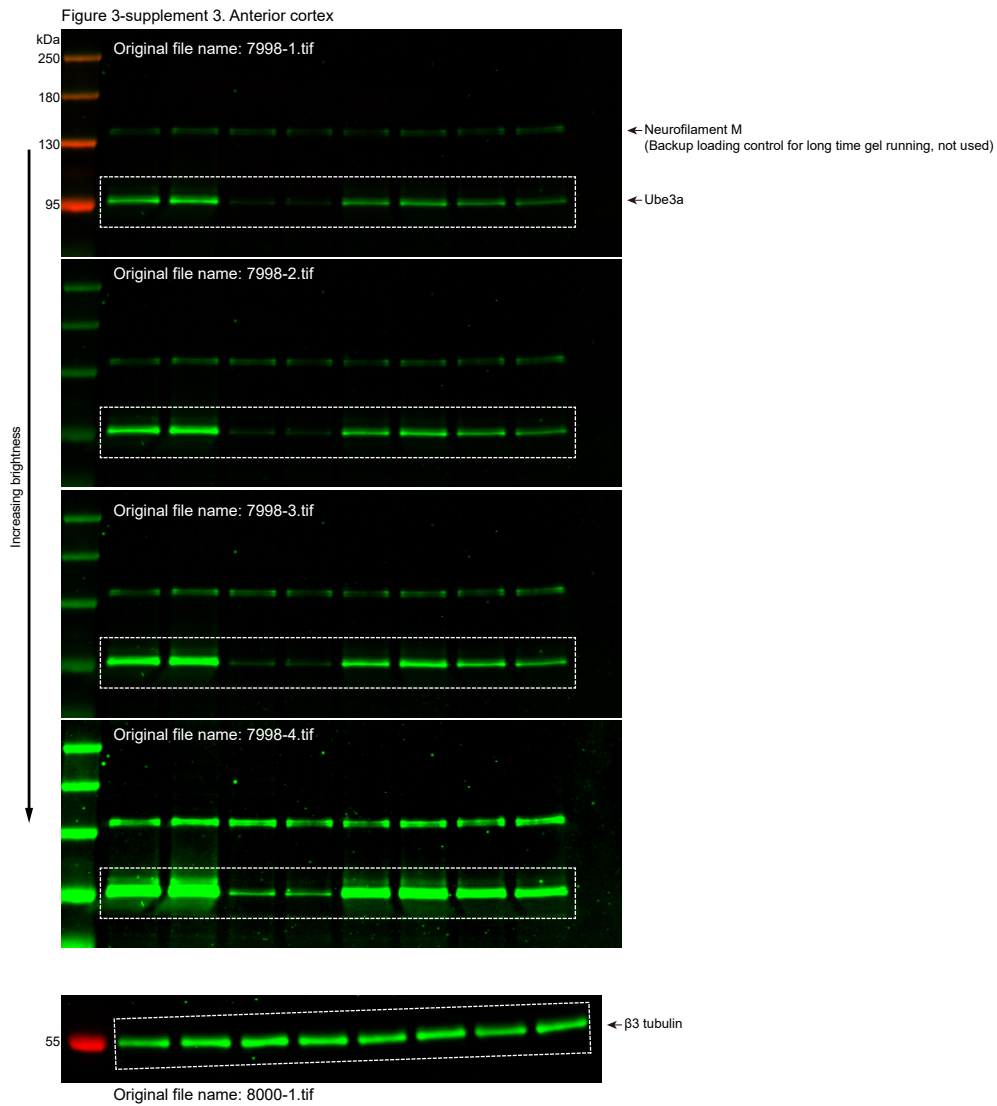

Supplement: Figure 3—figure supplement 3—source data 1. — The dashed boxes indicate the areas of blots presented in the figure. [file elife-81892-fig3-figsupp3-data1.zip › Figure 3-figure supplement 3-source data 1/Figure 3-figure supplement 3-source data 1 Ube3a isoforms WB juvenile p3wks ANT 221223.pdf]

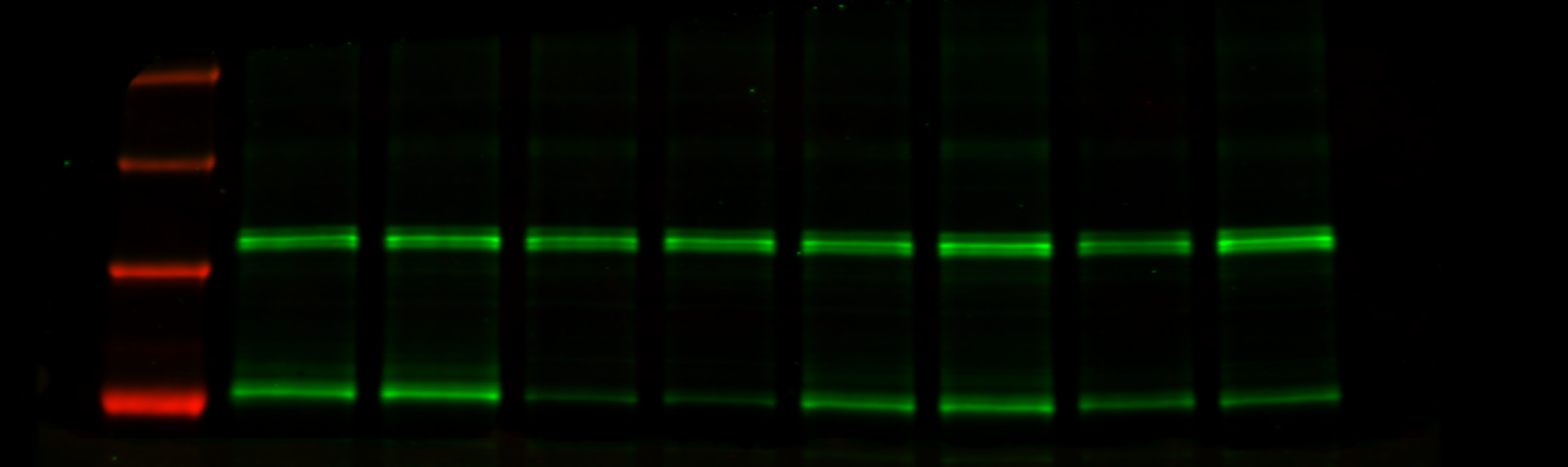

Supplement: Figure 3—figure supplement 3—source data 2. — The dashed boxes indicate the areas of blots presented in the figure. [file elife-81892-fig3-figsupp3-data2.zip › Figure 3-figure supplement 3-source data 2/8096-1.tif]

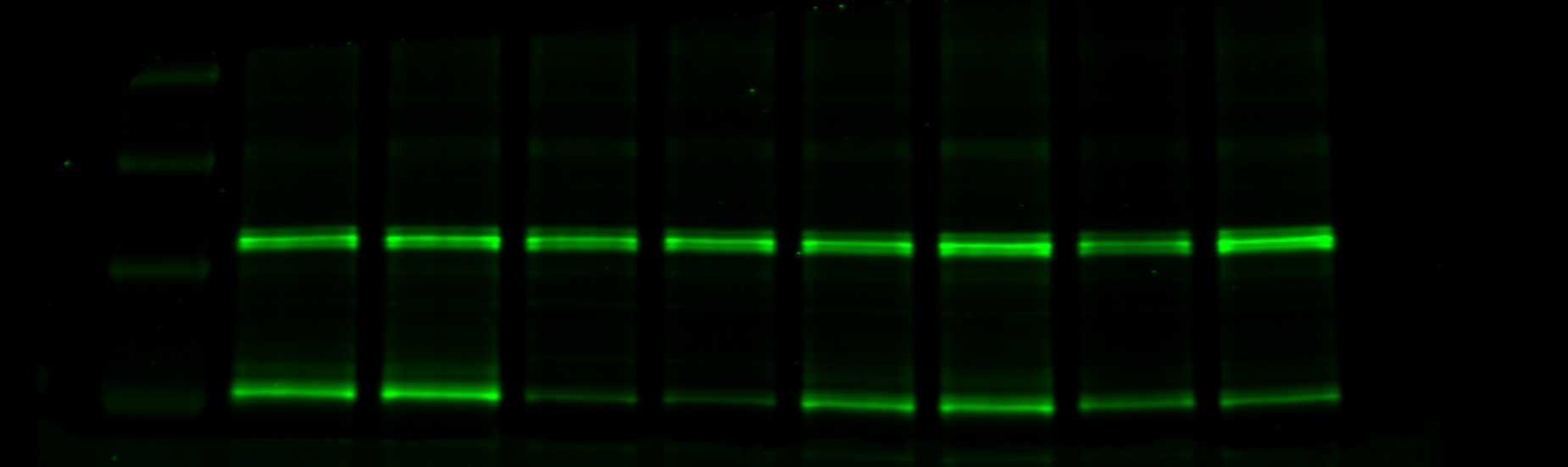

Supplement: Figure 3—figure supplement 3—source data 2. — The dashed boxes indicate the areas of blots presented in the figure. [file elife-81892-fig3-figsupp3-data2.zip › Figure 3-figure supplement 3-source data 2/8096-2.tif]

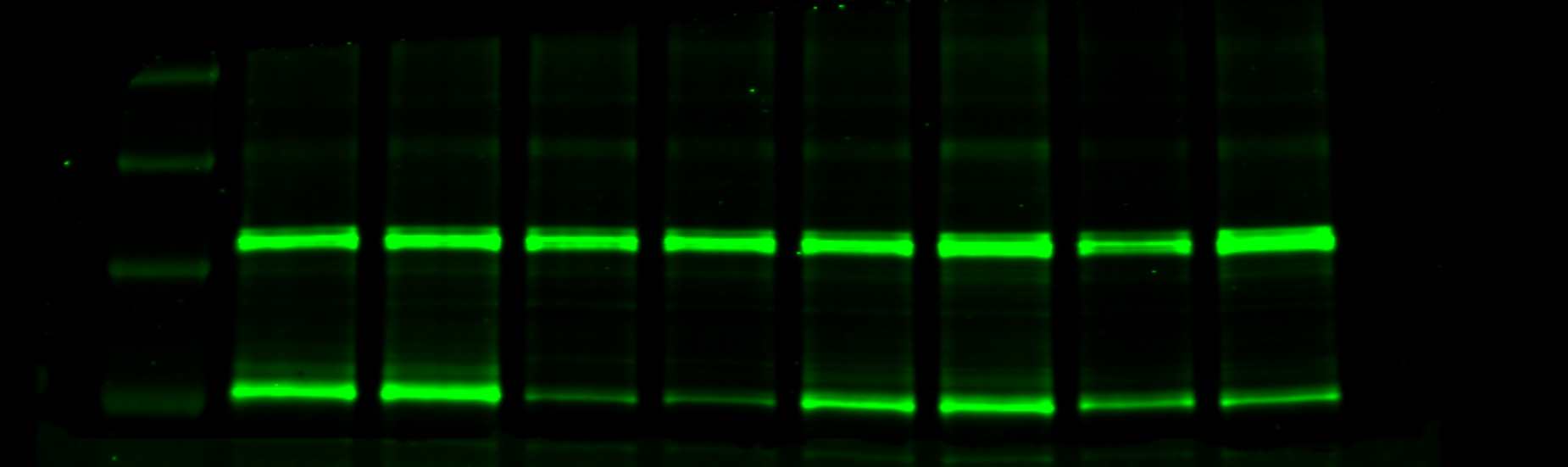

Supplement: Figure 3—figure supplement 3—source data 2. — The dashed boxes indicate the areas of blots presented in the figure. [file elife-81892-fig3-figsupp3-data2.zip › Figure 3-figure supplement 3-source data 2/8096-3.tif]

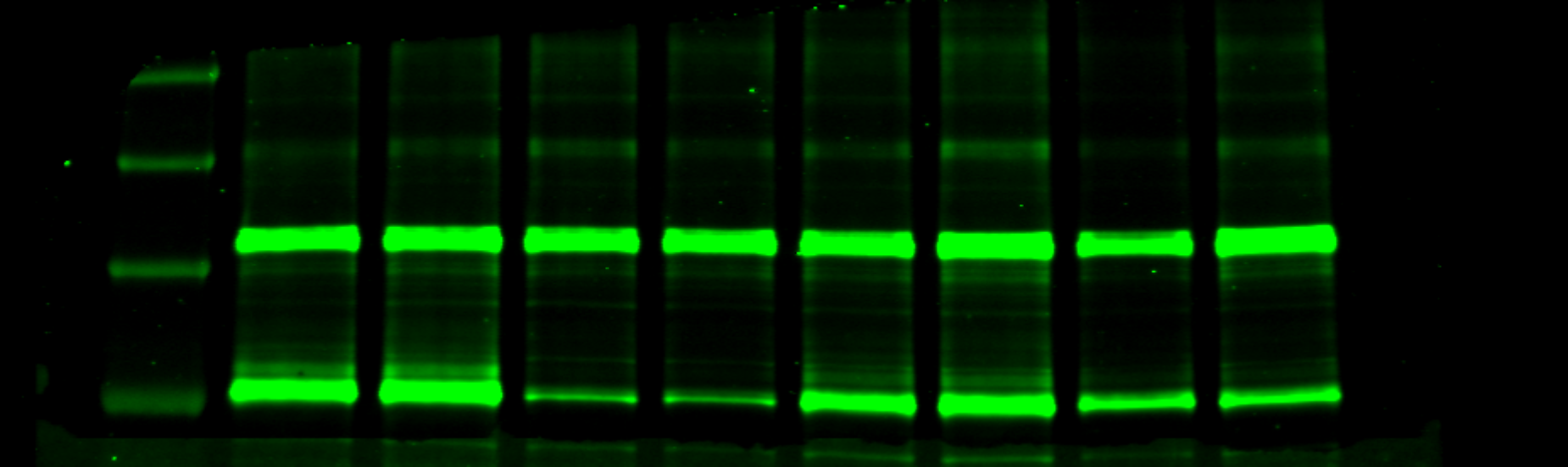

Supplement: Figure 3—figure supplement 3—source data 2. — The dashed boxes indicate the areas of blots presented in the figure. [file elife-81892-fig3-figsupp3-data2.zip › Figure 3-figure supplement 3-source data 2/8096-4.tif]

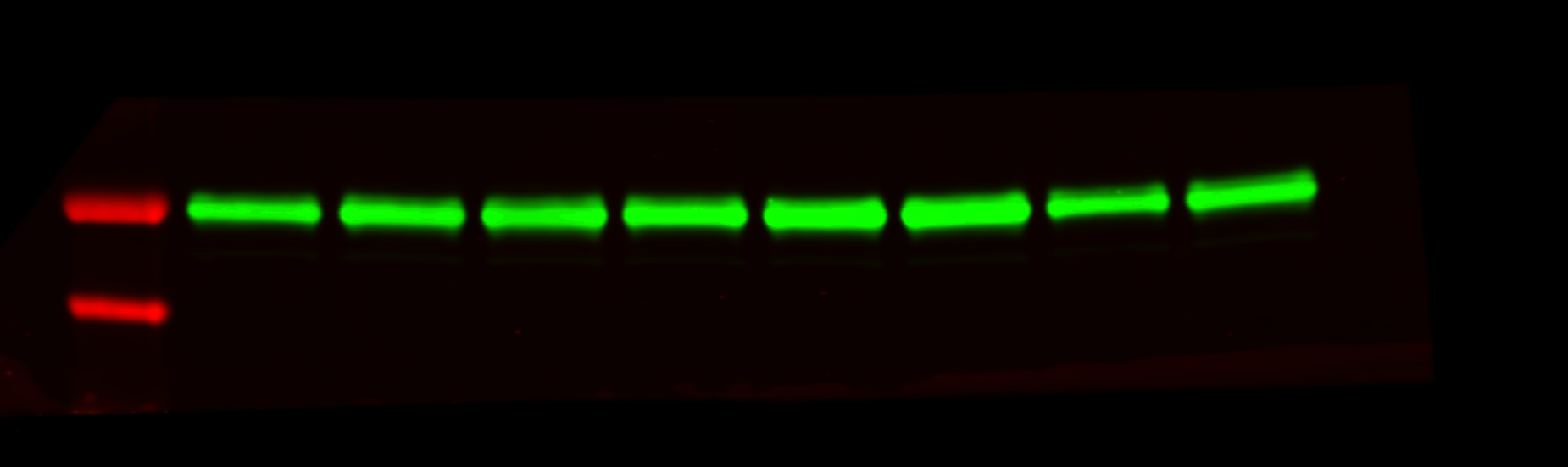

Supplement: Figure 3—figure supplement 3—source data 2. — The dashed boxes indicate the areas of blots presented in the figure. [file elife-81892-fig3-figsupp3-data2.zip › Figure 3-figure supplement 3-source data 2/8102-1.tif]

3 weeks post ASO injection to juvenile mice

Figure 3-supplement 3. Striatum

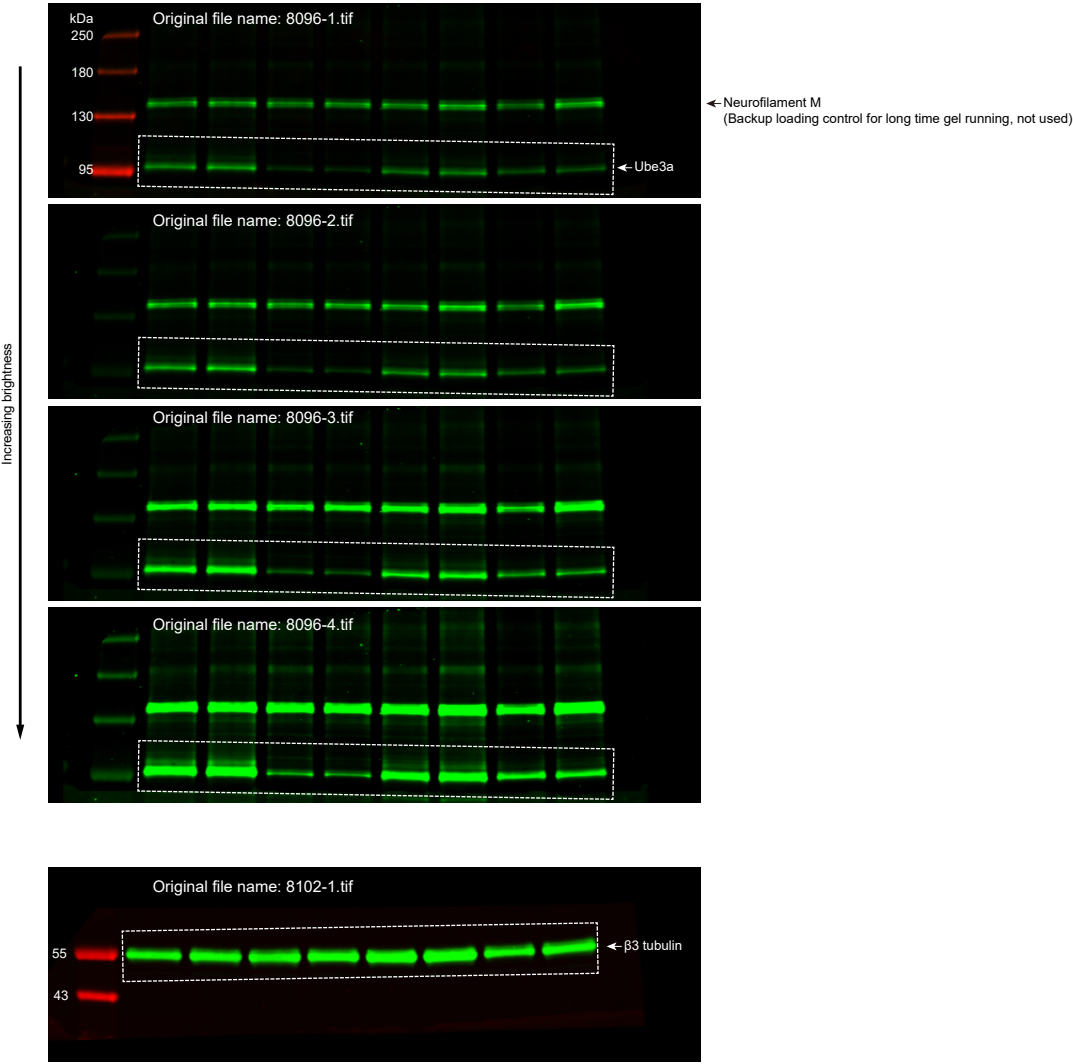

Supplement: Figure 3—figure supplement 3—source data 2. — The dashed boxes indicate the areas of blots presented in the figure. [file elife-81892-fig3-figsupp3-data2.zip › Figure 3-figure supplement 3-source data 2/Figure 3-figure supplement 3-source data 2 Ube3a isoforms WB juvenile p3wks STR 221223.pdf]

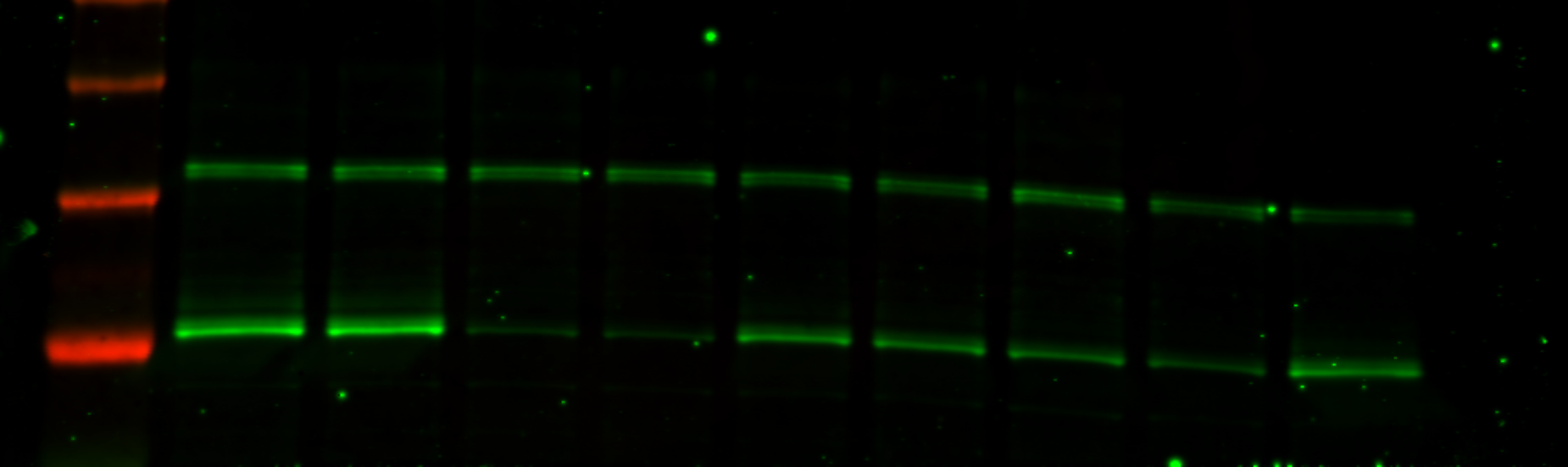

Supplement: Figure 3—figure supplement 4—source data 1. — The dashed boxes indicate the areas of blots presented in the figure. [file elife-81892-fig3-figsupp4-data1.zip › Figure 3-figure supplement 4-source data 1/8279-1.tif]

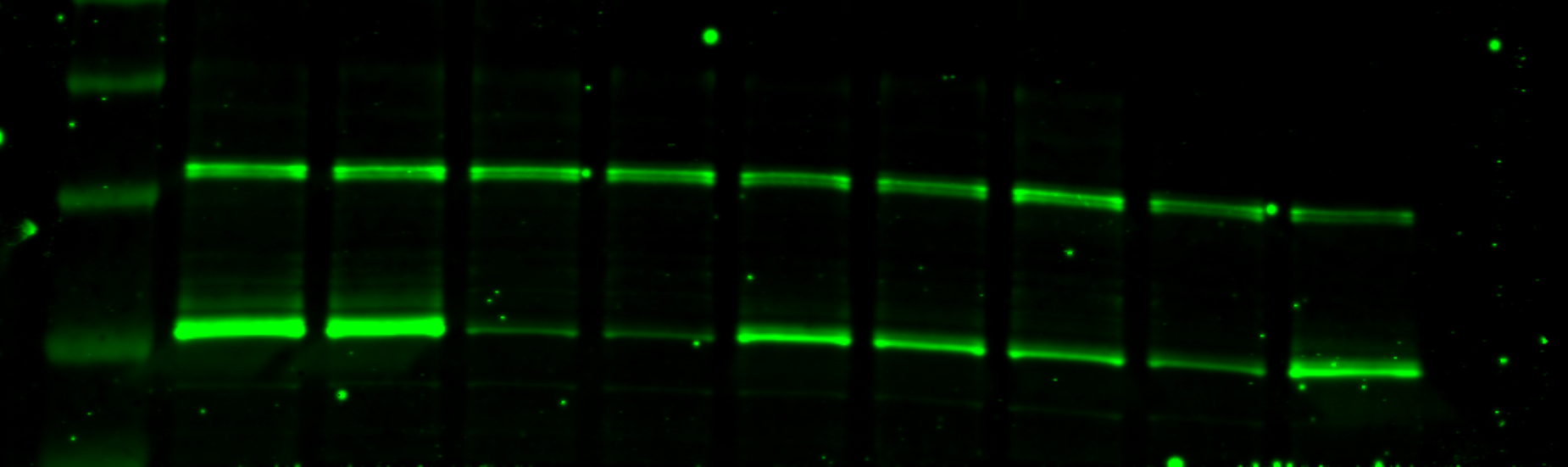

Supplement: Figure 3—figure supplement 4—source data 1. — The dashed boxes indicate the areas of blots presented in the figure. [file elife-81892-fig3-figsupp4-data1.zip › Figure 3-figure supplement 4-source data 1/8279-2.tif]

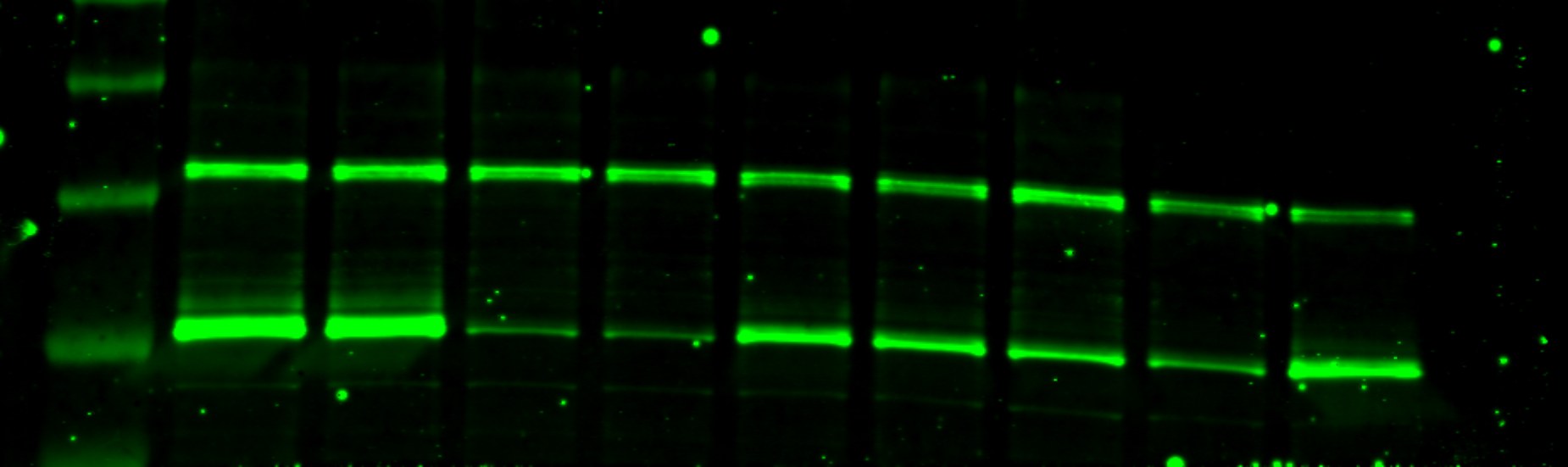

Supplement: Figure 3—figure supplement 4—source data 1. — The dashed boxes indicate the areas of blots presented in the figure. [file elife-81892-fig3-figsupp4-data1.zip › Figure 3-figure supplement 4-source data 1/8279-3.tif]

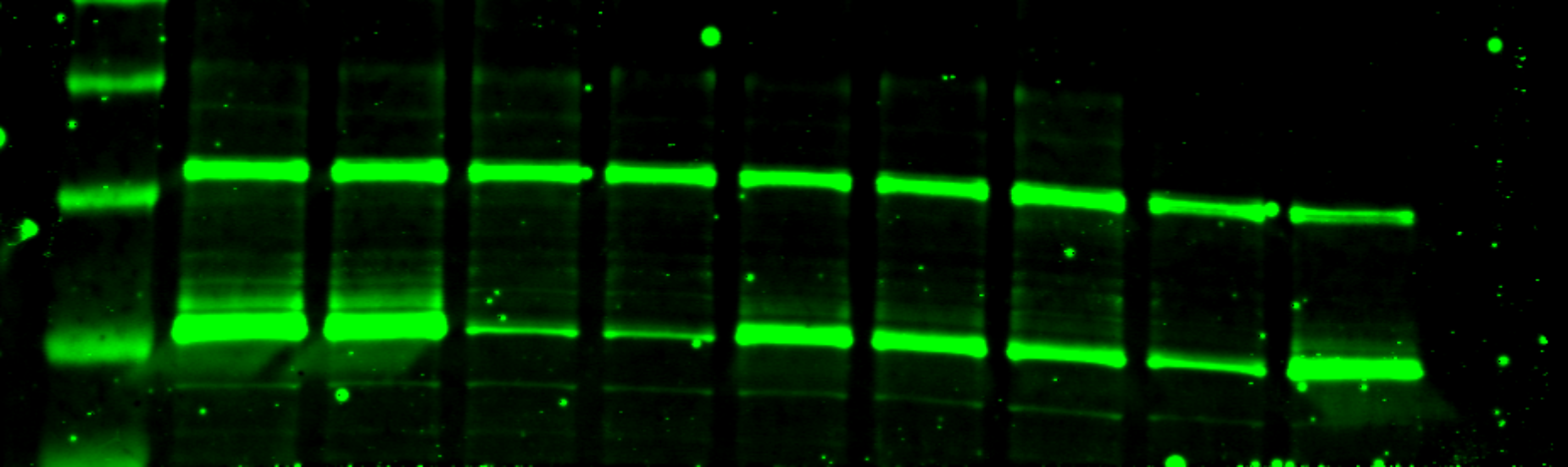

Supplement: Figure 3—figure supplement 4—source data 1. — The dashed boxes indicate the areas of blots presented in the figure. [file elife-81892-fig3-figsupp4-data1.zip › Figure 3-figure supplement 4-source data 1/8279-4.tif]

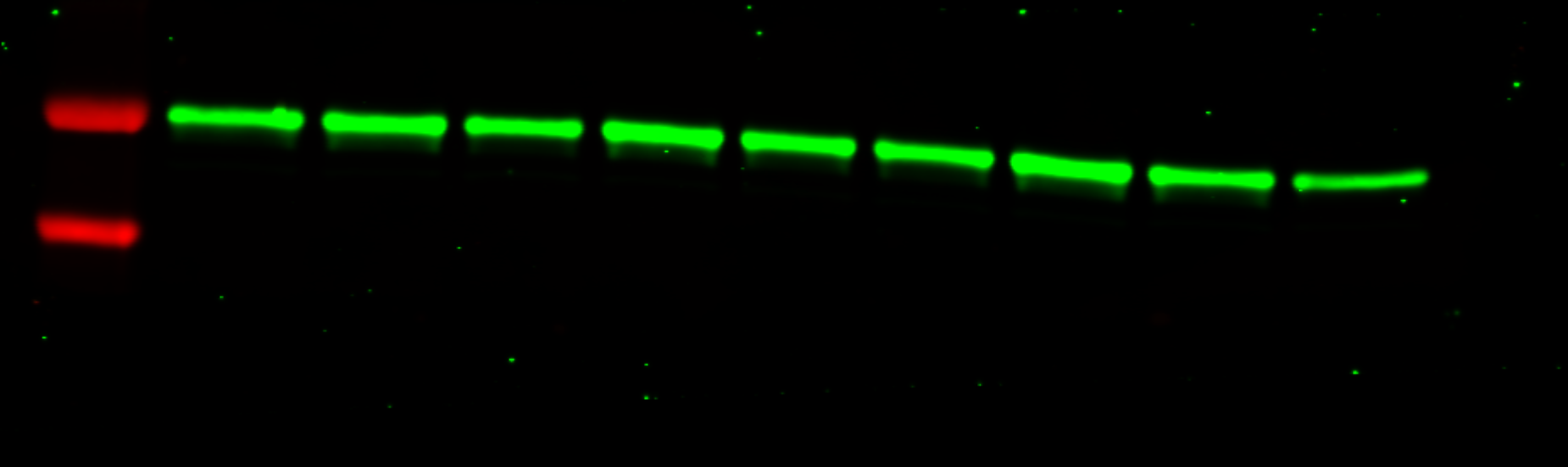

Supplement: Figure 3—figure supplement 4—source data 1. — The dashed boxes indicate the areas of blots presented in the figure. [file elife-81892-fig3-figsupp4-data1.zip › Figure 3-figure supplement 4-source data 1/8283-1.tif]

3 weeks post ASO injection to adult mice

Figure 3-supplement 4. Anterior cortex

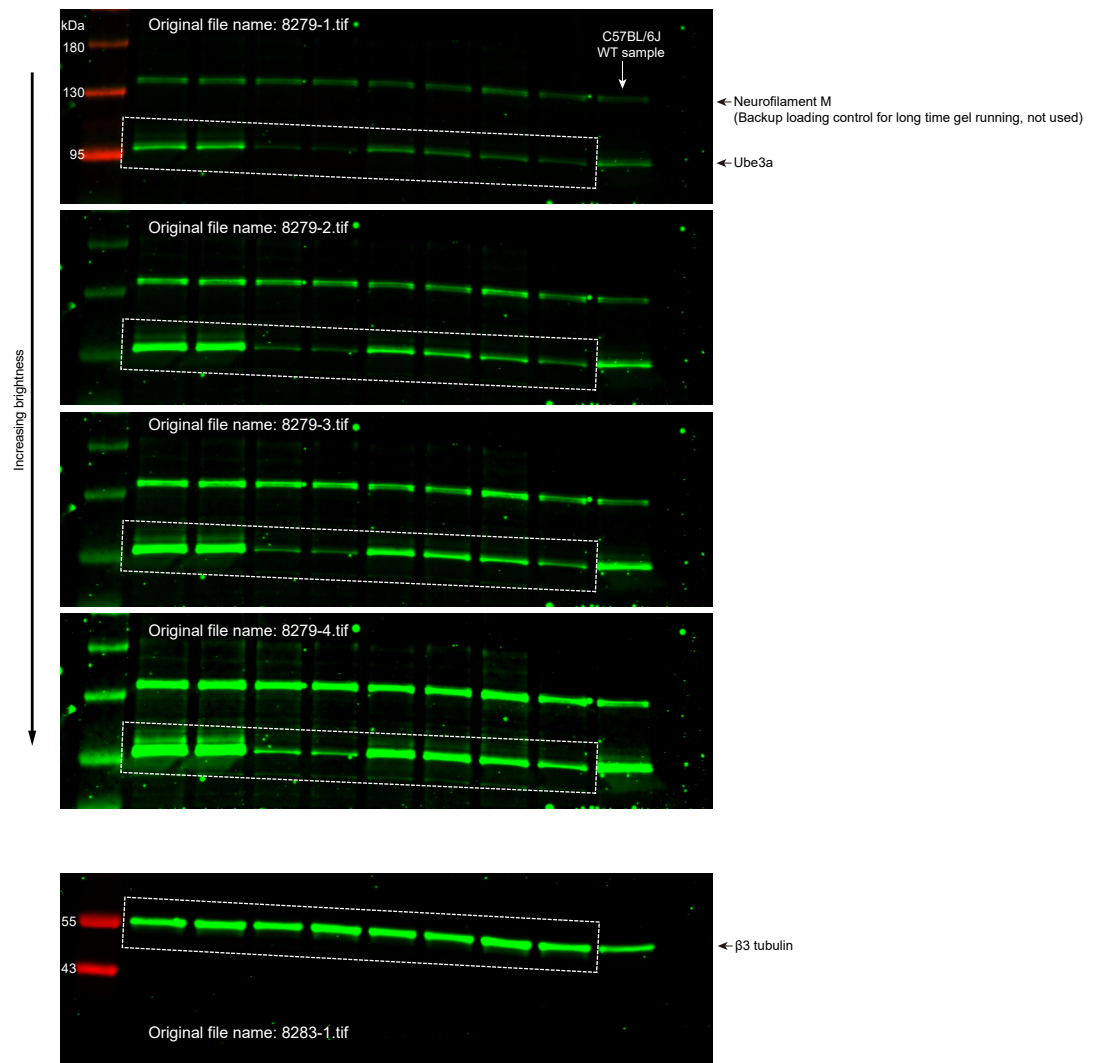

Supplement: Figure 3—figure supplement 4—source data 1. — The dashed boxes indicate the areas of blots presented in the figure. [file elife-81892-fig3-figsupp4-data1.zip › Figure 3-figure supplement 4-source data 1/Figure 3-figure supplement 4-source data 1 Ube3a isoforms WB adult p3wks ANT 221223.pdf]

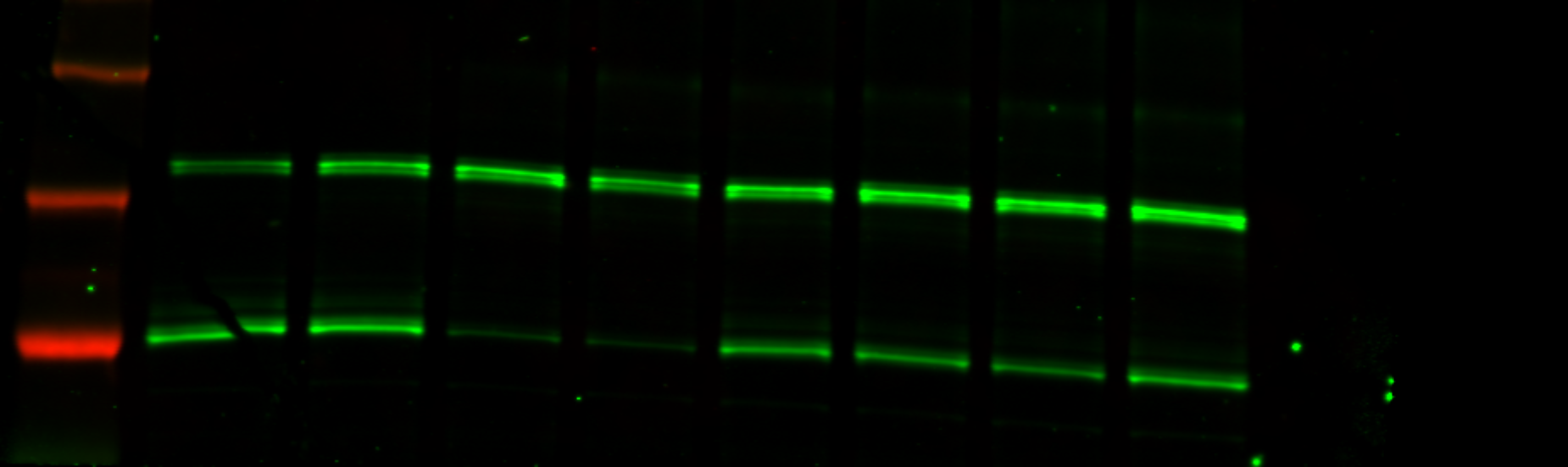

Supplement: Figure 3—figure supplement 4—source data 2. — The dashed boxes indicate the areas of blots presented in the figure. [file elife-81892-fig3-figsupp4-data2.zip › Figure 3-figure supplement 4-source data 2/8294-1.tif]

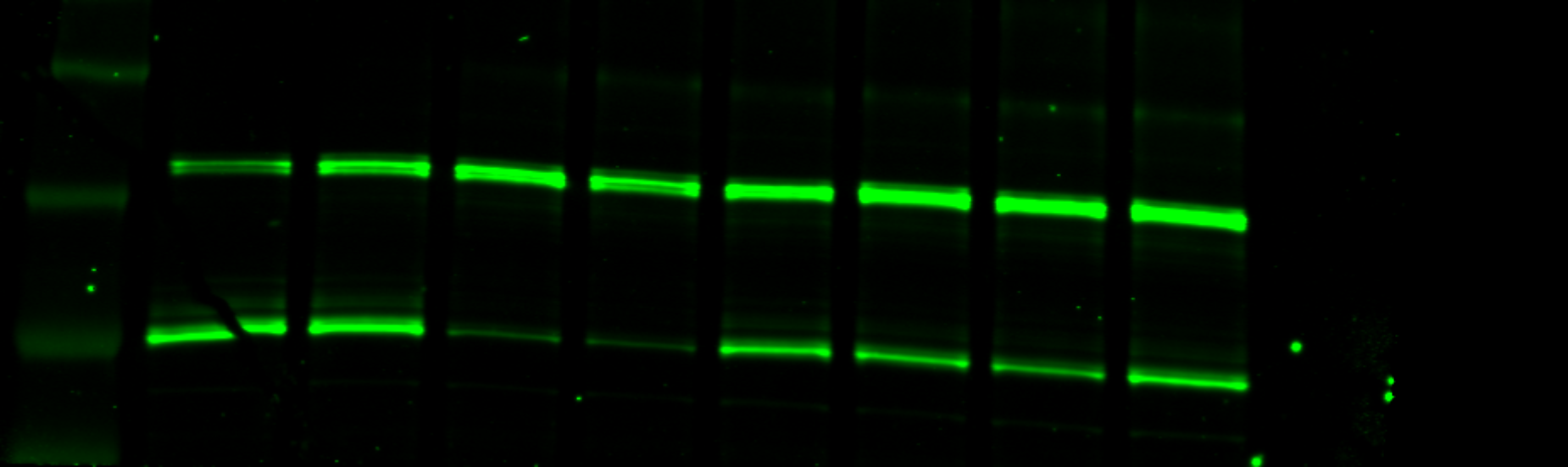

Supplement: Figure 3—figure supplement 4—source data 2. — The dashed boxes indicate the areas of blots presented in the figure. [file elife-81892-fig3-figsupp4-data2.zip › Figure 3-figure supplement 4-source data 2/8294-2.tif]

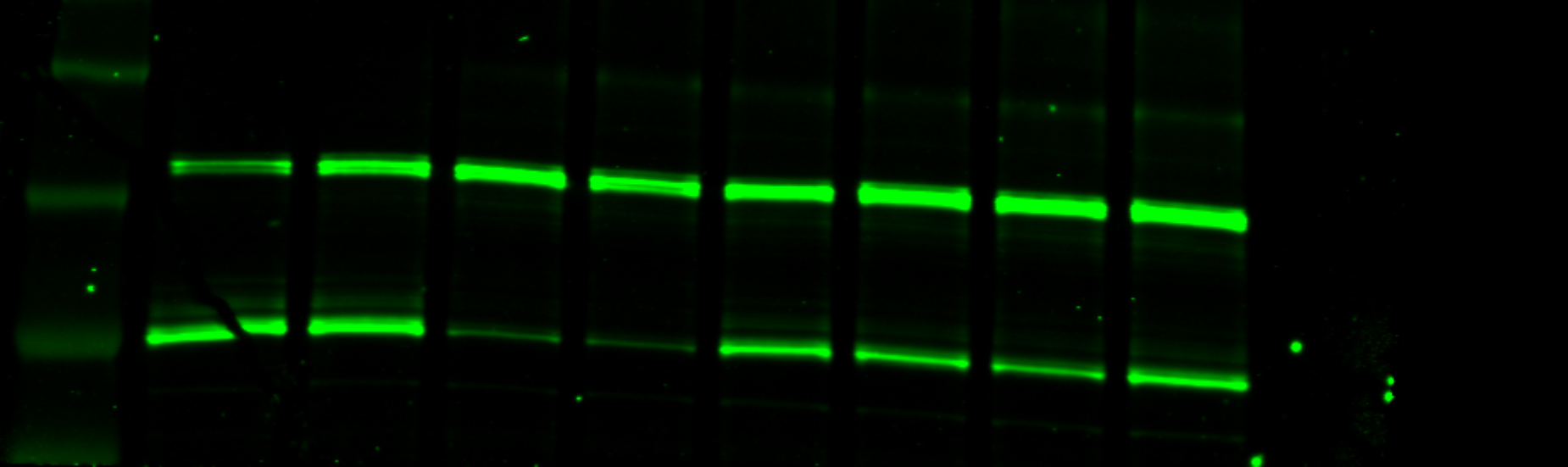

Supplement: Figure 3—figure supplement 4—source data 2. — The dashed boxes indicate the areas of blots presented in the figure. [file elife-81892-fig3-figsupp4-data2.zip › Figure 3-figure supplement 4-source data 2/8294-3.tif]

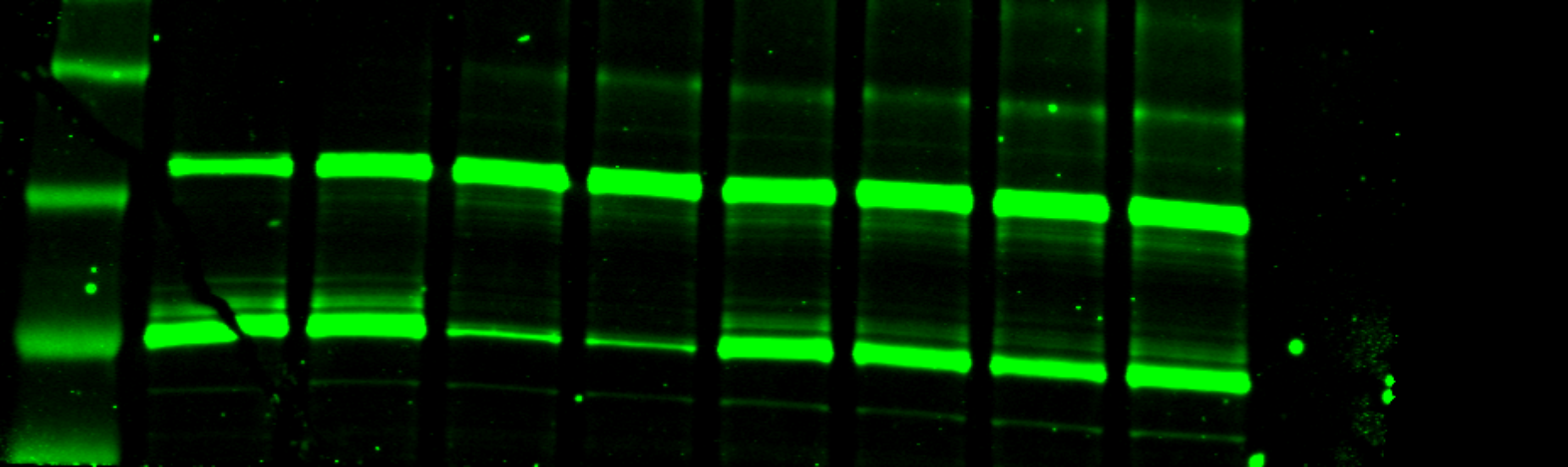

Supplement: Figure 3—figure supplement 4—source data 2. — The dashed boxes indicate the areas of blots presented in the figure. [file elife-81892-fig3-figsupp4-data2.zip › Figure 3-figure supplement 4-source data 2/8294-4.tif]

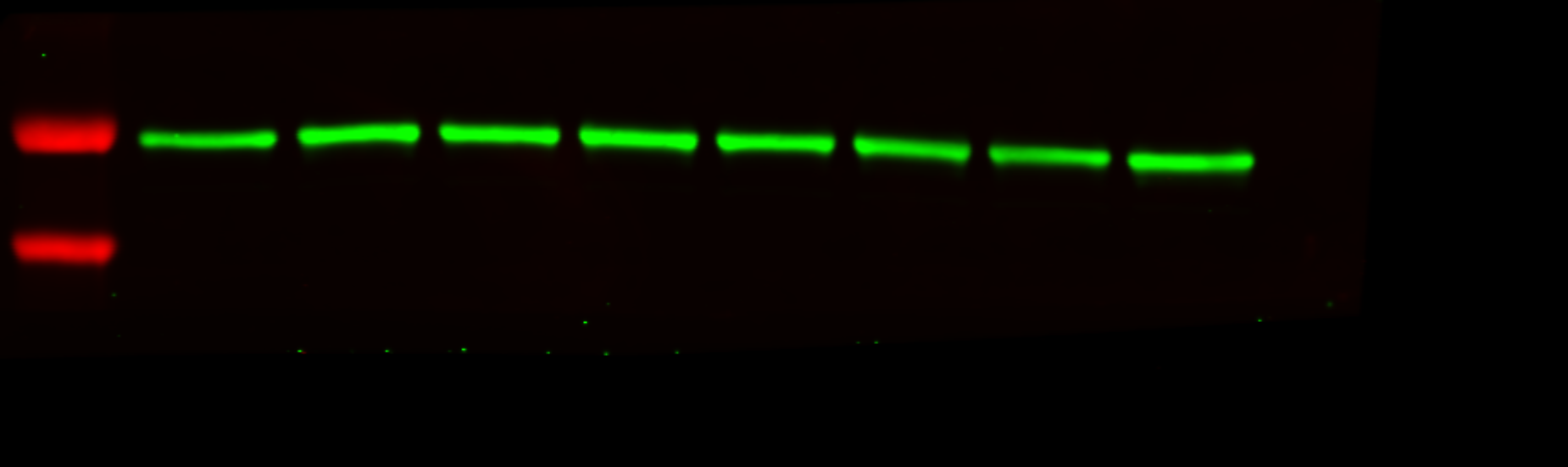

Supplement: Figure 3—figure supplement 4—source data 2. — The dashed boxes indicate the areas of blots presented in the figure. [file elife-81892-fig3-figsupp4-data2.zip › Figure 3-figure supplement 4-source data 2/8298-1.tif]

3 weeks post ASO injection to adult mice

Figure 3-supplement 4. Hippocampus

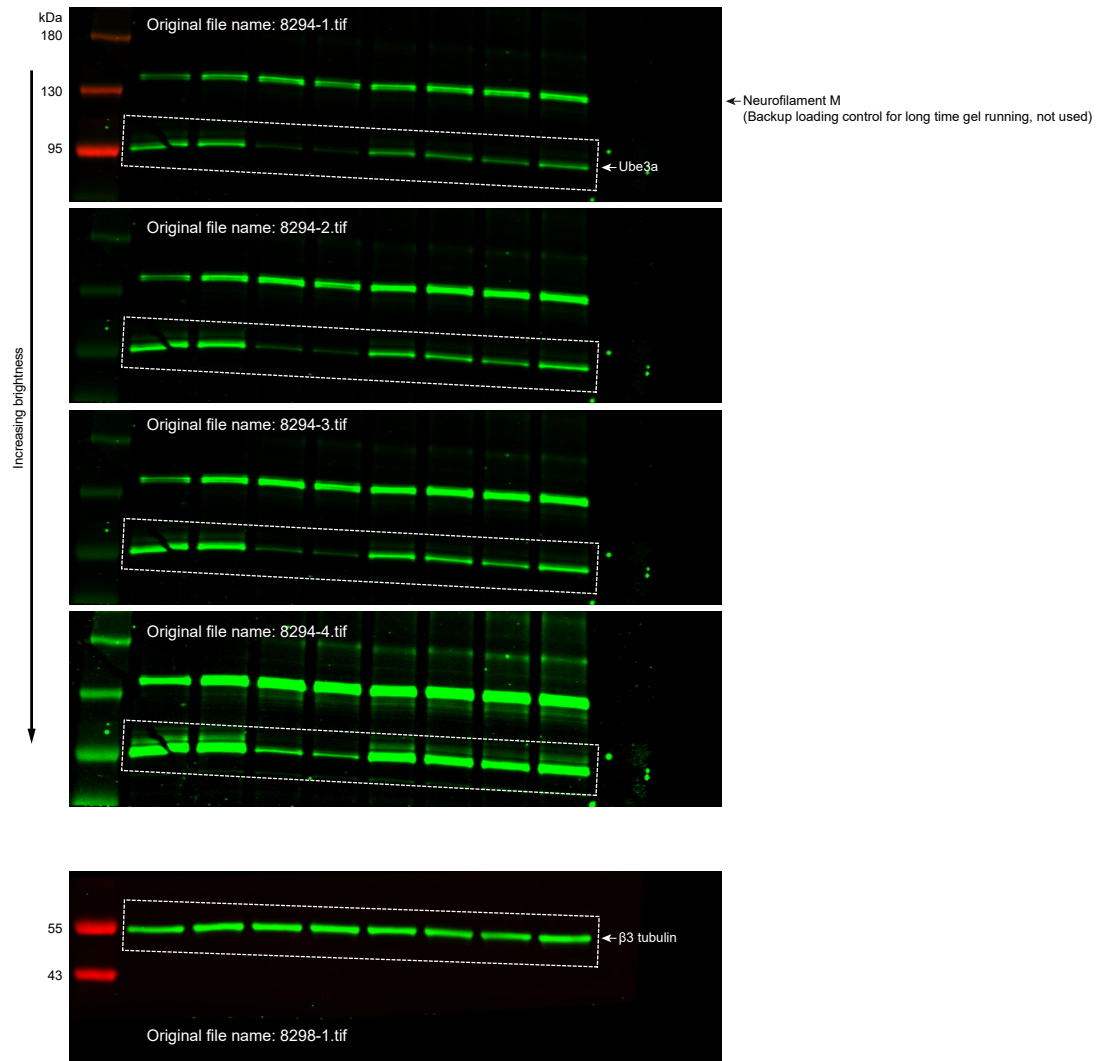

Supplement: Figure 3—figure supplement 4—source data 2. — The dashed boxes indicate the areas of blots presented in the figure. [file elife-81892-fig3-figsupp4-data2.zip › Figure 3-figure supplement 4-source data 2/Figure 3-figure supplement 4-source data 2 Ube3a isoforms WB adult p3wks HIP 221223.pdf]
