# Supplementary material for: Antisense oligonucleotide therapy rescues disturbed brain rhythms and sleep in juvenile and adult mouse models of Angelman syndrome
Source: eLife. 2023 Jan 3;12:e81892. doi: 10.7554/eLife.81892 (PMC9904759; doi:10.7554/eLife.81892)
Supplement: Figure 3—source data 2. — The dashed boxes indicate the areas of blots presented in the figure. [file elife-81892-fig3-data2.zip › Figure 3-source data 2/Figure 3-source data 2 WB adult p3wks p10wks 221223.pdf]

3 weeks post ASO injection to adult mice

Figure 3C. Anterior cortex

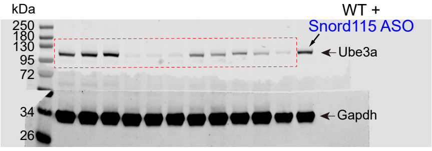

Original file name: 238-2.tif

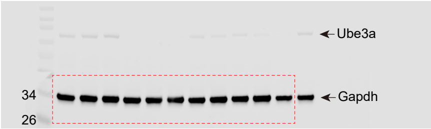

Original file name: 238-1.tif

Figure 3C. Hippocampus

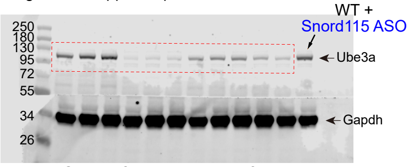

Original file name: 242-2.tif

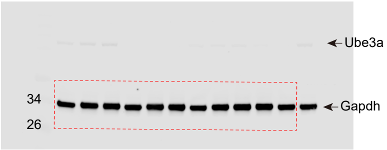

Original file name: 242-1.tif

10 weeks post ASO injection to adult mice

Figure 3C. Anterior cortex

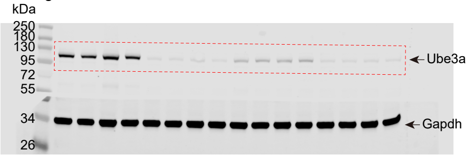

Original file name: 215-2.tif

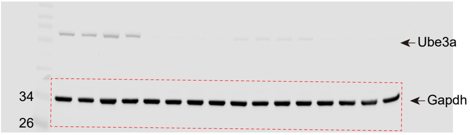

Original file name: 215-1.tif

Figure 3C. Hippocampus

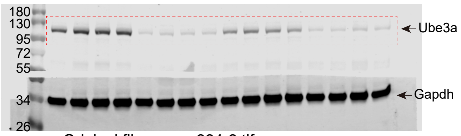

Original file name: 224-2.tif

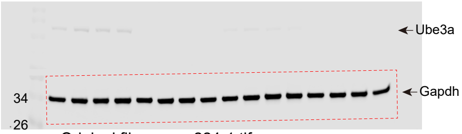

Original file name: 224-1.tif
