# Supplementary material for: Antisense oligonucleotide therapy rescues disturbed brain rhythms and sleep in juvenile and adult mouse models of Angelman syndrome
Source: eLife. 2023 Jan 3;12:e81892. doi: 10.7554/eLife.81892 (PMC9904759; doi:10.7554/eLife.81892)

3 weeks post ASO injection to juvenile mice

Figure 3-supplement 1A. Olfactory bulb

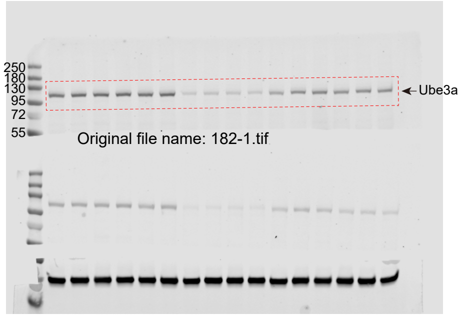

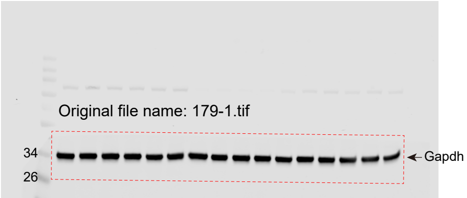

Figure 3-supplement 1A. Striatum

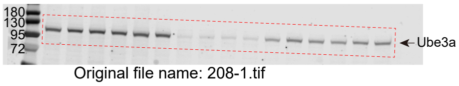

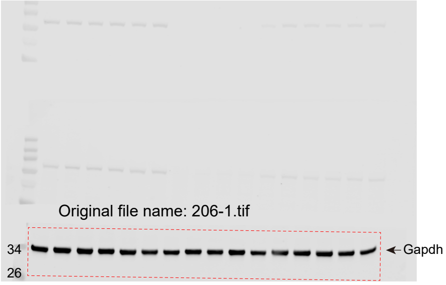

Figure 3-supplement 1A. Midbrain + Hindbrain

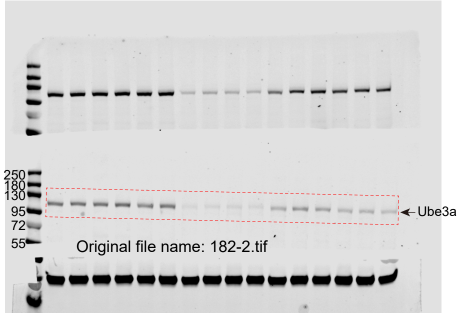

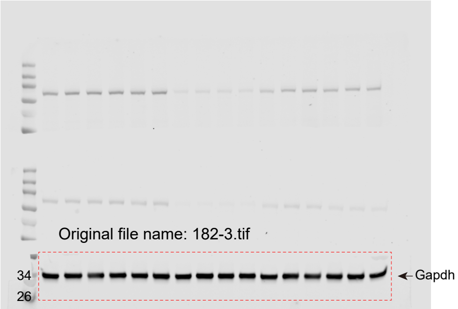

Supplement: Figure 3—figure supplement 1—source data 1. — The dashed boxes indicate the areas of blots presented in the figure. [file elife-81892-fig3-figsupp1-data1.zip › Figure 3-figure supplement 1-source data 1/Figure 3-figure supplement 1-source data 1b WB juvenile p3wks 221223.pdf]
