# Supplementary material for: Antisense oligonucleotide therapy rescues disturbed brain rhythms and sleep in juvenile and adult mouse models of Angelman syndrome
Source: eLife. 2023 Jan 3;12:e81892. doi: 10.7554/eLife.81892 (PMC9904759; doi:10.7554/eLife.81892)

10 weeks post ASO injection to juvenile mice

Figure 3-supplement 1A. Posterior cortex

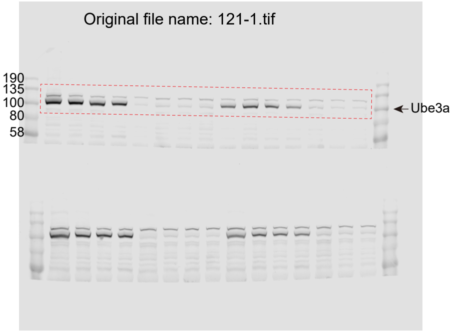

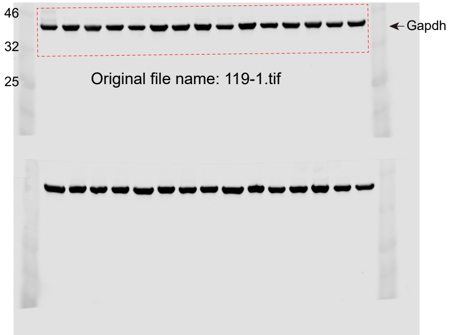

Figure 3-supplement 1A. Cerebellum

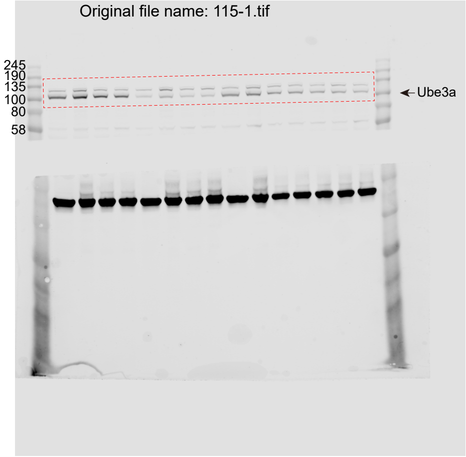

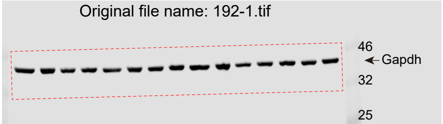

Figure 3-supplement 1A. Thalamus + Hypothalamus

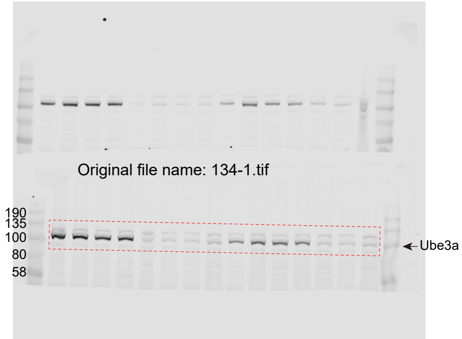

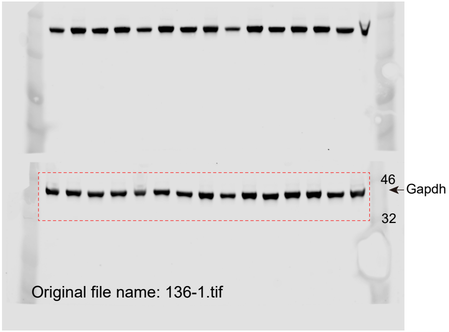

Supplement: Figure 3—figure supplement 1—source data 2. — The dashed boxes indicate the areas of blots presented in the figure. [file elife-81892-fig3-figsupp1-data2.zip › Figure 3-figure supplement 1-source data 2/Figure 3-figure supplement 1-source data 2a WB juvenile p10wks 221223.pdf]
