# Supplementary material for: Antisense oligonucleotide therapy rescues disturbed brain rhythms and sleep in juvenile and adult mouse models of Angelman syndrome
Source: eLife. 2023 Jan 3;12:e81892. doi: 10.7554/eLife.81892 (PMC9904759; doi:10.7554/eLife.81892)
Supplement: Figure 3—figure supplement 1—source data 4. — The dashed boxes indicate the areas of blots presented in the figure. [file elife-81892-fig3-figsupp1-data4.zip › Figure 3-figure supplement 1-source data 4/Figure 3-figure supplement 1-source data 4a WB adult p10wks 221223.pdf]

10 weeks post ASO injection to adult mice

Figure 3-supplement 1B. Posterior cortex

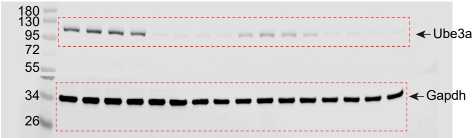

Original file name: 217-1.tif

Figure 3-supplement 1B. Cerebellum

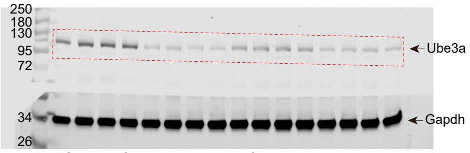

Original file name: 222-2.tif

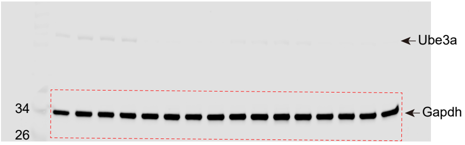

Original file name: 222-1.tif

Figure 3-supplement 1B. Thalamus + Hypothalamus

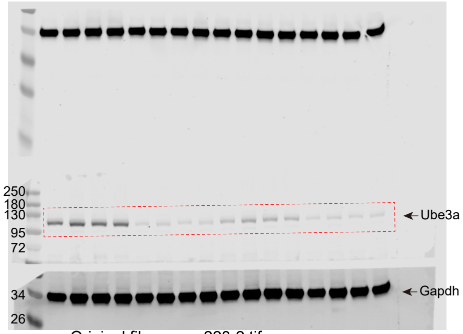

Original file name: 228-2.tif

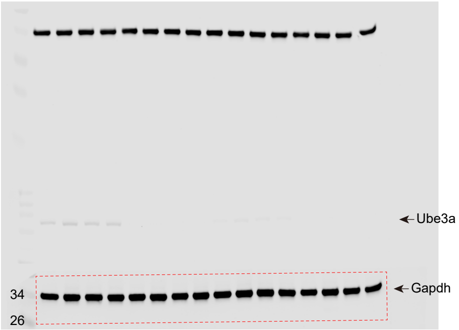

Original file name: 228-1.tif
